# Relationship between free-time physical activity and sleep quality in Brazilian university students

**DOI:** 10.1038/s41598-023-33851-3

**Published:** 2023-04-24

**Authors:** Mayara Santos, Rafaela Sirtoli, Renne Rodrigues, José Francisco López-Gil, Vicente Martínez-Vizcaíno, Camilo Molino Guidoni, Arthur Eumann Mesas

**Affiliations:** 1 Postgraduate Program in Public Health, Universidade Estadual de Londrina, Londrina, Parana Brazil; 2 Health and Social Research Center, Universidad de Castilla-La Mancha, Cuenca, Spain; 3 Facultad de Ciencias de la Salud, Universidad Autónoma de Chile, Talca, Chile; 4 Navarrabiomed, Hospital Universitario de Navarra, IdiSNA, Universidad Pública de Navarra, Pamplona, Spain; 5 Department of Environmental Health, Harvard T.H. Chan School of Public Health, Harvard University, Boston, United States of America; 6 One Health Research Group, Universidad de Las Américas, Quito, Ecuador

**Keywords:** Epidemiology, Risk factors, Statistics, Sleep disorders

## Abstract

Poor sleep quality and low or no free-time physical activity (FTPA) practice are highly prevalent among university students, but the association between these conditions is still unclear. This cross-sectional study analyzed the relationship between FTPA and sleep quality. An online questionnaire was conducted with university students from a public university in southern Brazil in 2019. The weekly frequency of FTPA was self-reported, and sleep quality was assessed using the Pittsburgh Sleep Quality Index (PSQI). Logistic regression and ANCOVA models were performed and adjusted for confounders. Among the 2,626 students analyzed, 52.2% did not practice the FTPA, and 75.6% had poor sleep quality (PSQI > 5). In the adjusted analysis, practicing FTPA 4–7 times/week was associated with poor sleep quality (odds ratio = 0.71; 95% confidence interval = 0.52, 0.97) compared with not practicing FTPA. In addition, those who practiced FTPA had significantly lower means of the global PSQI, subjective sleep quality and duration, sleep disturbances, and daytime dysfunction scores than those who did not practice FTPA. In conclusion, the FTPA may contribute to better sleep quality among university students.

## Introduction

The lifestyle changes that occur at the beginning of and throughout university studies, added to the demands of higher education, require a young adult's ability to adapt to the challenges of academic life^1^. In this period, students are exposed to high pressure, lack of time^2^, the need to adapt to the rules established by the institution^3^, low social support^2^, change from a familiar environment to another often unknown one, and adaptation to new social circles^3^. The consequences of these daily demands are intense and strenuous, influencing the academic performance and health of university students^3^.

In this population group, the proportion of individuals with poor sleep quality, identified according to a Pittsburgh Sleep Quality Index (PSQI) score greater than 5 points, is particularly high, reaching proportions between 62.0% and 87.1%^4–6^. Importantly, because the PSQI is a subjective sleep assessment method, these high proportions might be affected, at least in part, by reporting bias^7^. However, thus far, it is not possible to consider that these proportions are overestimated because the agreement between subjective and objective (i.e., actigraphy or polysomnography) sleep measures in this population is still unclear^8^, and in addition, no large-scale epidemiological study in university students from various fields of knowledge has used objective sleep measurements. In addition to the harmful implications of sleep disorders on other health conditions, such as excess weight^9^, chronic musculoskeletal pain^10^ and high blood pressure^9^, their potential relationship with mental health conditions deserves special attention in university students, such as depression and anxiety^4^, stress, low quality of life^11^, suicidal ideation and attempt^12^, and lower academic performance^13,14^. Among the possible strategies to reduce the prevalence of sleep disorders and attenuate their consequences, free-time physical activity (FTPA), i.e., any form of physical exercise or movement performed during leisure time, outside of work or other obligations, is a nonpharmacological alternative that has shown beneficial effects^5^. Consistently, it has been observed that physically inactive students are more likely to have poor sleep quality^5,15^.

Given the changes in students’ lifestyles during academic life, it is necessary to understand the relationship between physical activity and sleep at this stage of life. Although physical activity, regardless of the domain, is particularly relevant^16^, the practice performed in free time has shown substantial benefits^17^. In addition, understanding this relationship may help establish behavioral interventions within the university environment, providing a better quality of sleep and, consequently, a better quality of life and general health. Therefore, this study aimed to analyze the relationship between the practice of FTPA and sleep quality in university students. To expand the available evidence on this association^18^, the role of FTPA on each of the PSQI sleep quality dimensions was investigated. Furthermore, major confounding factors of the FTPA-sleep association, such as sociodemographic, anthropometric, lifestyle, and mental health symptoms, were adjusted in the analysis.

## Results

### Sociodemographic and lifestyle characterization

The study population (2,626) was predominantly female (68.1%), with a mean age of 21.9 ± 4.5 years, ranging from 18 to 54 years (11.8% had > 25 years). The mean BMI was 23.7 ± 4.7 kg/m^2^, and 73.3% of the university students reported depressive symptoms. More than half (52.2%) of the participants did not practice FTPA at least once a week. The proportion of poor sleep quality was 79.6% among those who did not practice FTPA, 72.3% in those who practiced FTPA 1–3 times/week, and 67.8% in those who practiced FTPA 4–7 times/week (Table 1).

**Table 1:** Sociodemographic, lifestyle and health characterization of Brazilian university students according to sleep quality.

Variables	TOTAL	PSQI global score, mean (SD)	Poor sleep quality (PSQI global score > 5)	Good sleep quality (PSQI global score ≤ 5)	p-value*
University students, n (%)	2,626 (100.0)	7.8 (3.1)	1986 (100.0)	640 (100.0)	
Sex, n (%)					< 0.001
Male	837 (31.9)	7.2 (2.9)	577 (29.1)	260 (40.6)	
Female	1,789 (68.1)	8.1 (3.2)	1,409 (70.9)	380 (59.4)	
Age (years), mean (SD)	21.9 (4.5)		22.0 (4.4)	21.8 (4.7)	0.585
BMI (kg/m^2^), mean (SD)	23.7 (4.7)		23.8 (4.8)	23.2 (4.3)	0.004
Alcohol intake, n (%)					0.011
No consumption or up to 1 time/month	1,729 (65.8)	7.7 (3.2)	1,281 (64.5)	448 (70.0)	
More than 1 time/month	897 (35.5)	8.1 (3.1)	705 (35.5)	192 (30.0)	
Tobacco smoking, n (%)					< 0.001
No	1,939 (73.8)	7.6 (3.1)	1,421 (71.6)	518 (80.9)	
Yes	687 (26.2)	8.5 (3.2)	565 (28.4)	122 (19.1)	
Fruit consumption, n (%)					0.040
5 to 7 days/week	2,136 (81.3)	7.9 (3.1)	1,633 (82.2)	503 (78.6)	
< 5 days/week	490 (18.7)	7.6 (3.3)	353 (17.8)	137 (21.4)	
Social support, n (%)					< 0.001
High	1,258 (47.9)	7.4 (3.0)	895 (45.1)	363 (56.7)	
Intermediate	1,061 (40.4)	8.1 (3.1)	823 (41.4)	238 (37.2)	
Low	307 (11.7)	9.0 (3.3)	268 (13.5)	39 (6.1)	
Depressive symptoms, n (%)					< 0.001
No	701 (26.7)	5.5 (2.2)	315 (15.9)	386 (60.3)	
Yes	1,925 (73.3)	8.7 (3.0)	1,671 (84.1)	254 (39.7)	
Free-time physical activity, n (%)					< 0.001
4–7 times/week	311 (11.8)	7.3 (3.0)	211 (10.6)	100 (15.6)	
1–3 times/week	944 (36.0)	7.4 (2.9)	683 (34.4)	261 (40.8)	
No practice	1,371 (52.2)	8.3 (3.2)	1,092 (55.0)	279 (43.6)	

*PSQI* Pittsburgh Sleep Quality Index, *SD* standard deviation, *BMI* body mass index.

### Free-time physical activity and poor sleep

In the crude analysis, a lower likelihood of reporting poor sleep quality was observed in those who practiced the FTPA 1–3 times during the week (OR 0.66; 95% CI 0.55, 0.80) or 4–7 times/week (OR 0.54; 95% CI 0.42, 0.71) than in those who did not practice the FTPA. This association remained statistically significant for those practicing FTPA with both 1–3 and 4–7 times/week frequencies when the models were adjusted for age and sex (Model 1) and weight status and other lifestyle variables (Model 2). When social support and depressive symptoms were included in the adjustment, only practicing FTPA 4–7 times/week remained associated with a lower likelihood of poor sleep quality (OR 0.71; 95% CI 0.52, 0.97) (Table 2).

**Table 2:** Association* between free-time physical activity and poor sleep quality (PSQI > 5) in Brazilian university students.

Exposure	Crude model		Adjusted models					
			Model 1		Model 2		Model 3	
	OR	95% CI	OR	95% CI	OR	95% CI	OR	95% CI
Free-time physical activity								
4–7 times/week	0.54	0.42, 0.71	0.61	0.46, 0.80	0.64	0.48, 0.85	0.71	0.52, 0.97
1–3 times/week	0.66	0.55, 0.80	0.70	0.57, 0.84	0.70	0.57, 0.85	0.82	0.66, 1.02
None	1.00		1.00		1.00		1.00	

*PSQI* Pittsburgh Sleep Quality Index, *OR* odds ratio, *CI* confidence interval.

*Logistic regression model for estimating the OR and 95% CI of the association between free-time physical activity (independent variable) and poor sleep quality (dependent variable). “None” was selected as a reference group. Model 1 included the adjustment variables sex and age. Model 2 added weight status, alcohol intake, tobacco smoking and fruit consumption to Model 1. Model 3 added social support and the presence of depressive symptoms to Model 2.

### Estimated marginal means of the Pittsburgh Sleep Quality Index by free-time physical activity

**Figure 1:**
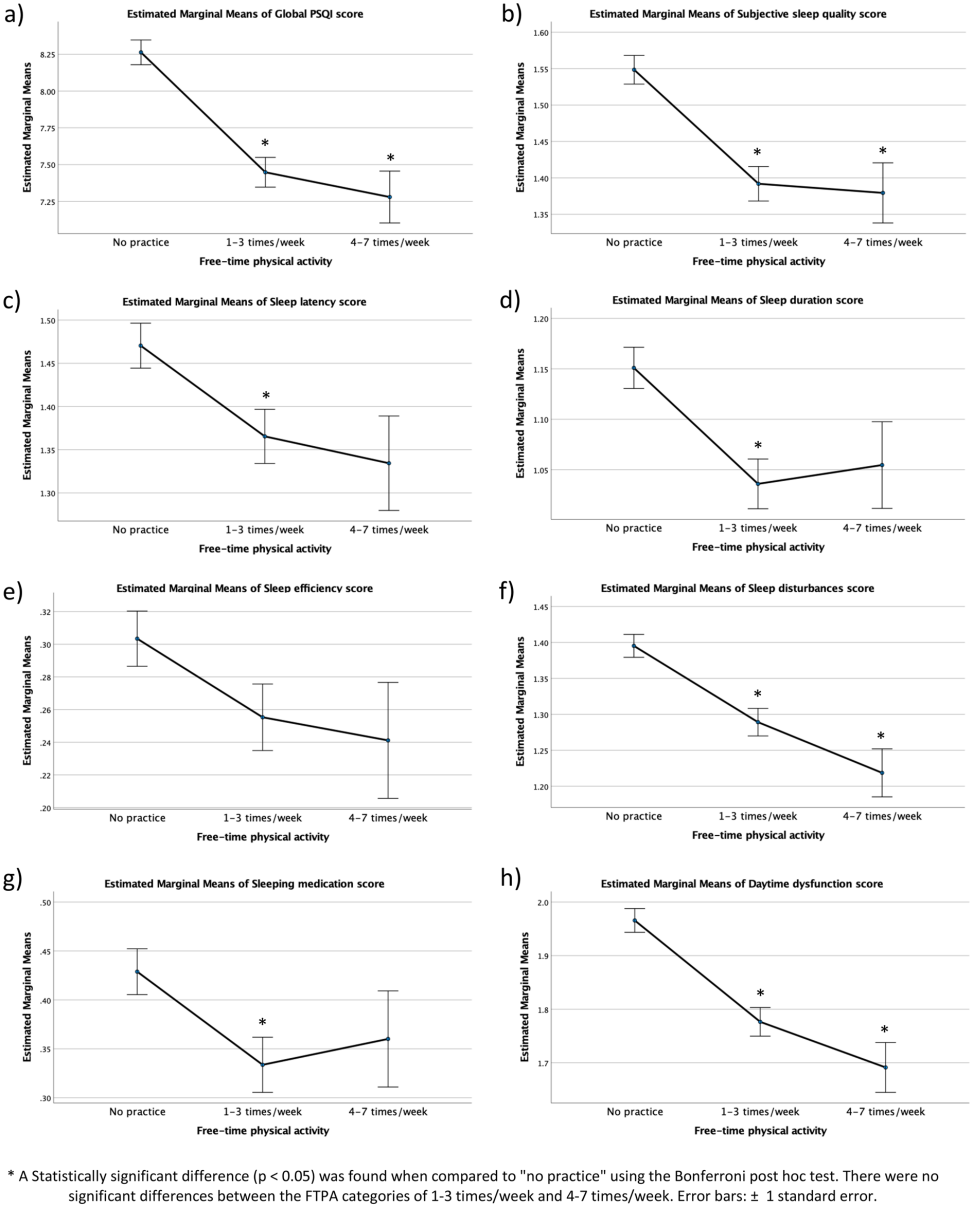
Raw estimated marginal means of the Pittsburgh Sleep Quality Index score and its components by frequency of free-time physical activity in Brazilian university students. Values were obtained with unadjusted ANCOVA models.

When considering sleep quality based on the PSQI score and its components, in the unadjusted analyses (Fig. 1), we observed lower estimated marginal means (indicating better sleep quality parameters) of the global PSQI score (Fig. 1a), subjective sleep quality score (Fig. 1b), sleep disturbances score (Fig. 1f) and daytime dysfunction score (Fig. 1h) in students who reported practicing FTPA 1–3 or 4–7 times/week than in those who did not practice FTPA. Moreover, significantly lower means were found for sleep latency (Fig. 1c), sleep duration (Fig. 1d) and sleep medication scores (Fig. 1-g) in students practicing FTPA 1–3 times/week.

**Figure 2:**
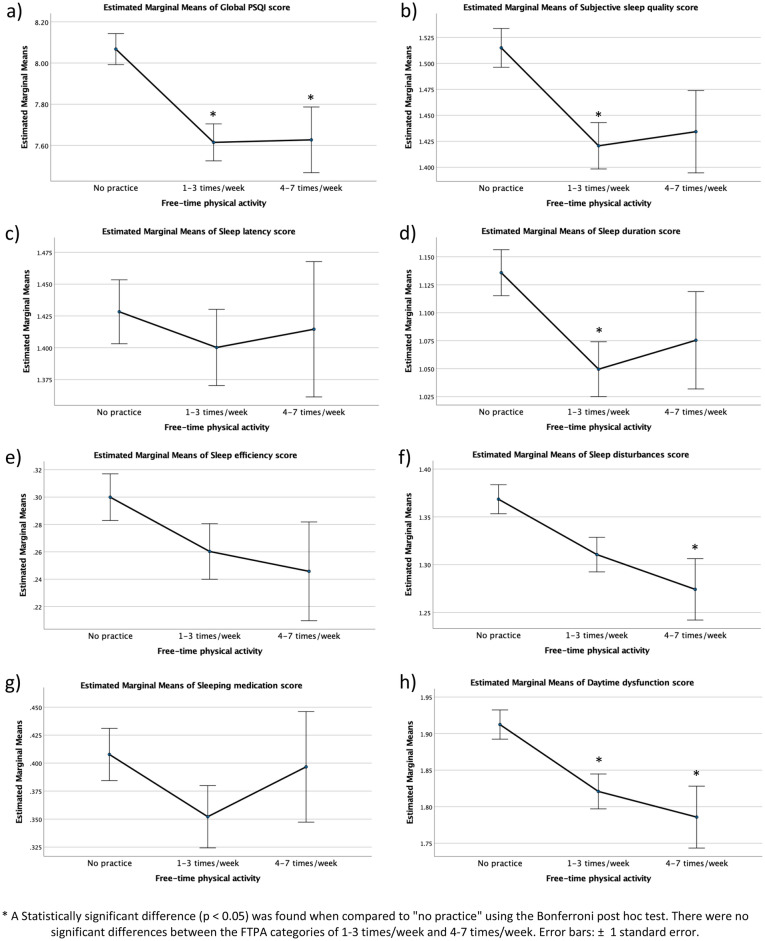
Adjusted estimated marginal means of the Pittsburgh Sleep Quality Index score and its components by frequency of free-time physical activity in Brazilian university students. Values were obtained with ANCOVA models adjusted for sex, age, weight status, alcohol intake, tobacco smoking, fruit consumption, social support and depressive symptoms.

When these analyses were adjusted for all confounders considered in this study (Fig. 2), the following associations remained statistically significant: both FTPA 1–3 or 4–7 times/week and lower scores of the global PSQI (Fig. 2a) and daytime dysfunction (Fig. 2h); only 1–3 times/week and lower scores of subjective sleep quality (Fig. 2b) and sleep duration (Fig. 2d); and only 4–7 times/week and lower scores of sleep disturbances (Fig. 2f).

## Discussion

The results of this study analyzing Brazilian university students indicated that the likelihood of reporting poor sleep quality was 28% lower among those who practiced the FTPA 4–7 times during the week than among those who did not practice the FTPA, regardless of the main confounders, including depressive symptoms. In addition, practicing FTPA 1–3 times/week was associated with significant improvements in subjective sleep quality, sleep duration and daytime dysfunction. Likewise, practicing FTPA 4–7 days/week was associated with benefits in sleep quality considering the global, sleep disturbances and daytime dysfunction dimension scores.

The proportion of poor sleep quality identified among the university students in this study (75.6%) is higher than that found in studies with similar populations in other countries. A cross-sectional study with 7,626 students from six universities in the United States found that 62.0% of the participants had poor sleep quality^4^, which is comparable to the 63.9% prevalence of poor sleep quality identified among Asian students^5^. In a cohort of 582 Chinese university students, the prevalence of poor sleepers (PSQI > 5 points) at baseline was 50.3%^19^. Despite these differences, the proportion of poor sleep quality affects more than half of the participants, confirming that this is a problem of great magnitude in the university population.

The lower propensity of having poor sleep quality among university students who practiced FTPA is consistent with the body of evidence available. A meta-analysis of predominantly cross-sectional studies with university students found that moderate- to high-intensity physical activity was associated with better quality sleep, with significant differences among countries^18^. Consistently, a study of Asian university students found that sleep duration, daytime dysfunction, and global PSQI scores differed significantly according to levels of physical activity^5^. A similar conclusion was obtained in a longitudinal study of Canadian university students, in which the authors claimed that moderate levels of physical activity predicted sleep quality indirectly (through emotion control) over time^20^. In contrast, a cross-sectional study with US university students reported that exercise frequency was not a significant predictor of sleep quality (PSQI scores)^21^. This divergence regarding the present study is possibly due to the definition of the physical activity variable because while they asked about the amount of time spent in exercises^21^, we asked about the number of times per week the students practiced FTPA. Although the data available for this study do not allow classification according to the type or intensity of FTPA, our findings contribute to scientific knowledge by identifying that even a measure of physical activity based only on weekly frequency was associated with lower means on the global score of the PSQI, as well as some of its dimensions (i.e., subjective sleep quality, sleep duration, sleep disturbances and daytime dysfunction).

These findings can be partially explained because physical exercise increases total sleep time, especially NREM sleep, and improves sleep quality, which generates more intense electrical brain waves, resulting in improvements in some domains of the PSQI^18^. Evidence supports the physiological effect of physical activity as an effective intervention for individuals who do not experience adequate sleep quantity or quality^18^. Daily moderate-intensity exercise was a potential factor in improving circadian melatonin rhythm, rectal temperature during nighttime sleep, sleep stages, and heart rate variability in healthy and clinical subjects^22^. The variations occur by activation of the sympathetic and parasympathetic nervous systems in an intercalated manner according to the time of physical activity and bedtime^22^.

Some methodological considerations should be made for the correct interpretation of our results. Because of the cross-sectional design, this research does not allow us to establish a causal relationship between practicing physical activity during free time and improving sleep quality. However, previous evidence supports the direction of this association, attributing to physical activity a predictive effect for better sleep quality^18^. Although sedentary behavior is associated with both variables of interest^23,24^, information on this behavior was not available for the present study. Although accelerometers and pedometers are used as objective measures to assess this practice^25^, questionnaires are commonly employed in epidemiological studies^26^. However, another limitation is that the information on FTPA was limited to weekly frequency, and because the type, intensity, and time dedicated to each physical activity were not specified, it cannot be inferred that students who practice it 4–7 times/week are necessarily more active than those who practice it 1–3 times/week. In addition, the high proportion of poor sleep quality found in this study, for both inactive students and for those practicing FTPA, may be related to selection bias that tends to overestimate the occurrence of health problems when dealing with a convenience sample. Furthermore, information on sleep quality was obtained through the interviewee’s self-report, and thus, the accuracy of the results may be affected by recall bias. Finally, while the large sample size allowed controlling for potential confounders such as social support and depressive symptoms, residual confounding is possible due to variables not available in the analyzed dataset, such as sports practice, use of sleep-related supplements (e.g., melatonin), chronic pain, stress and other diseases and health conditions.

The results of this study suggest that the practice of FTPA may contribute to increased sleep quality in university students, independent of sociodemographic factors and mental health status. Our findings have beneficial implications in the process of encouraging the practice of FTPA since it is closely related to beneficial health outcomes, particularly sleep quality in university students. Due to the high proportion of the outcome studied, it is necessary to implement multidisciplinary intervention programs through behavioral strategies and the promotion of sleep hygiene measures in university students.

## Methods

### Design, population and study location

This is a cross-sectional study, part of a project conducted at a public university in Brazil. All university students aged ≥ 18 years who regularly enrolled during the collection period were invited. During the study period, the university had 12,462 undergraduates distributed across nine study centers and 50 undergraduate courses. The following inclusion criteria were considered: 18 years of age or older and actively enrolled in a face-to-face undergraduate course in the first half of 2019.

**Figure 3:**
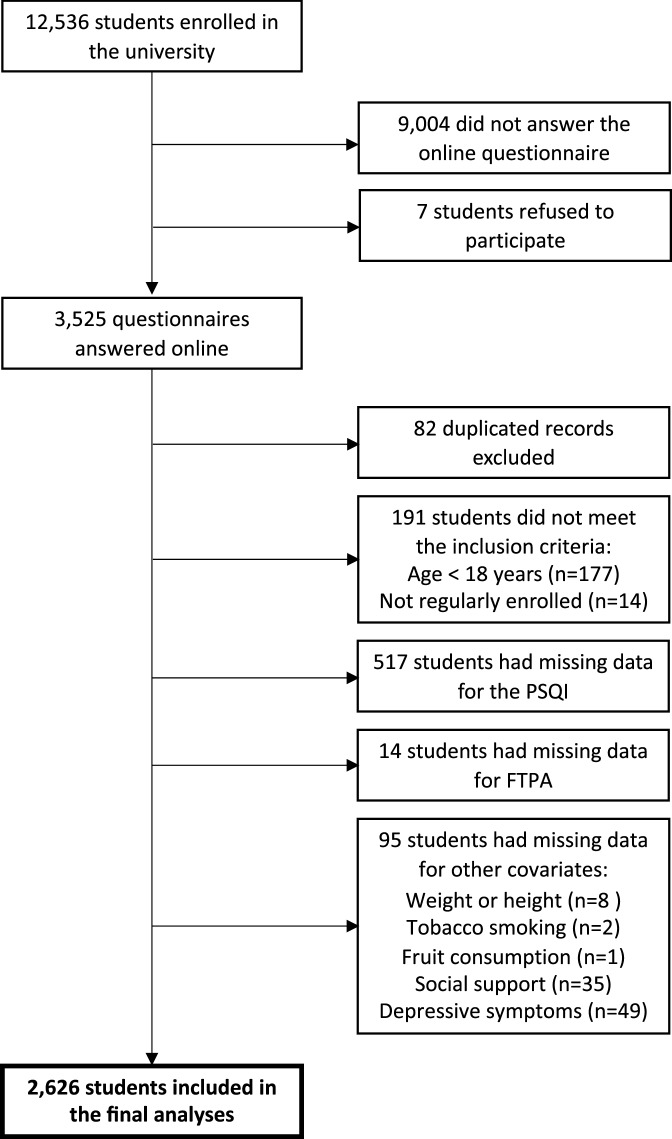
Flow diagram of the study participants.

Among the 12,536 university students eligible for the study, 3525 completed the questionnaire (initial response rate of 28.1%) (Fig. 3). From these, 82 duplicate records and 191 questionnaires from students who did not meet the inclusion criteria (177 under 18 years old and 14 not regularly enrolled) were excluded. Of the remaining 3252, 517 students for the PSQI, 14 for FTPA and 95 for other covariates considered in the analysis were excluded due to missing data, resulting in 2626 students (20.9%) being analyzed in this study (Fig. 3).

### Project dissemination and data collection

The project was widely disseminated through face-to-face visits by the research team to all 259 undergraduate classrooms of the university and through flyers, posters and content published in the university's internal communication channels (i.e., TV channel, radio, official website, social networks, etc.). All these measures were aimed at inviting as many students as possible to participate in the study.

Data collection was conducted between April 29 and June 28, 2019. For data collection, students should access an online questionnaire inserted into the Google Forms® digital platform using a link available in all the aforementioned disclosure options, which was also sent to each student's institutional email. Before having access to the questions, a message allowed students to participate only once and anonymously, with the insertion of their registration number being optional. Duplicate fill-ins were excluded.

### Study variables

Sleep quality (dependent variable) was obtained using the Brazilian Portuguese version of the PSQI^27,28^. This tool is composed of seven components (subjective sleep quality, sleep latency, sleep duration, sleep efficiency, sleep disturbances, use of sleep medications, and daytime dysfunction), classified on a scale from zero to three, which are added to generate a global score ranging from 0 to 21 points. The component scores were used continuously, and the global PSQI score was used continuously and categorically (> 5 points were considered students with poor sleep quality)^27,28^. The following cutoff points were defined: for sleep duration, values between 3 and 12 h were accepted; for sleep latency, values between 0 and 300 min were accepted; and for sleep efficiency, values above 100% were excluded.

Data on free-time physical activity (FTPA) (independent variable) were obtained through the following question: “In a typical week, how often do you practice physical activity in your free time?”. The answer options were does not practice, practice once a week, two to three times a week, and four or more times a week. Subsequently, the variable was categorized into (a) none (no practice); (b) 1–3 times/week; and (c) 4–7 times/week.

The following covariates were also included in the analysis based on the available literature^2,15,18,20–22,29^ on their potential confounding effect on the association between FTPA and sleep quality: sex (female vs. male); age (in years); body mass index (BMI) (in kg/m^2^, calculated according to self-reported weight and height); alcohol intake (no consumption or up to 1 time/month vs. more than 1 time/month); tobacco smoking (yes vs. no); frequency of fruit consumption, as an indicator of diet quality (< 5 days/week vs. 5 to 7 days/week); and social support, measured with the Social Support Scale (MOS-SSS) (high, intermediate, and low)^30^. Moreover, the depressive symptoms variable was obtained using the Patient Health Questionnaire-9 (PHQ-9)^31^, a scale that assesses the frequency of nine depressive symptoms in the last two weeks. Each item is rated on a scale from zero to three, and the total score ranges from zero to 27 points. The depressive symptoms score was classified as follows: (a) absence (< 9 points) or (b) presence (≥ 9 points)^31^.

### Statistical analysis

Descriptive analysis was performed using the absolute and relative frequencies of categorical variables, and for continuous variables, means and standard deviations were calculated. The chi-square test was used to analyze differences in the frequency of poor sleep quality according to the other categorical variables studied. Comparisons between means of continuous variables were analyzed using Student’s t test for unpaired data after confirming normal distribution using the Kolmogorov–Smirnov test.

To analyze the association between FTPA practice (independent variable) and sleep quality (dichotomous dependent variable), the crude and adjusted odds ratios (ORs) were calculated, with their respective 95% confidence intervals (CIs) through logistic regression models with progressive and cumulative introduction of groups of variables to examine the confounding effect of each group added to the effect of the variables introduced in the previous model. After performing a crude model, an adjusted Model 1 was built including the sociodemographic variables sex and age. In sequence, Model 2 included BMI in addition to sex and age. Finally, a fully adjusted Model 3 included the previous adjustment variables and the presence of depressive symptoms.

We also tested whether the estimated marginal means of the global PSQI score and its components differed according to the category of FTPA using analysis of covariance (ANCOVA) models adjusted for sex, age, BMI and the presence of depressive symptoms.

The data were analyzed using the Statistical Package for the Social Sciences software (IBM Corp. Released 2016. IBM SPSS Statistics for Windows, Version 28.0. Armonk, NY: IBM Corp.). For all analyses, a statistical significance level of p < 0.05 was considered.

### Ethical aspects

The project was approved by the Human Research Ethics Committee of the State University of Londrina, Brazil (protocol number: 04456818.0.0000.5231), and all participants were informed about the objectives of the study, guarantee of anonymity and nonuse of the data for other purposes. All participants signed an informed consent form before having access to answer the study questions. All research procedures were performed in accordance with the Declaration of Helsinki and other relevant guidelines and institutional regulations applied for studies involving human participants.

## Supplementary Information

Supplementary Information.

## Data Availability

All data generated or analyzed during this study are included in this published article and Supplementary Information.
