# Supplementary material for: Relationship between free-time physical activity and sleep quality in Brazilian university students
Source: Sci Rep. 2023 Apr 24;13:6652. doi: 10.1038/s41598-023-33851-3 (PMC10125990; doi:10.1038/s41598-023-33851-3)
Supplement: Supplementary file 1 — Supplementary Information. [file 41598_2023_33851_MOESM1_ESM.pdf]

ID	SEX	AGE	AGE25UP	IMC	IMC_3CT	ALCOHOL	TOBACCO	FRUITS	SSUPPORT_3CT	DEPRE	PA_3CT
1	1	19	1	22.7	1	0	0	0	2	2	2
2	2	20	1	26.8	2	1	0	0	3	1	1
5	1	27	2	18.8	1	1	0	0	3	2	2
6	1	21	1	27.7	2	0	0	0	3	1	2
7	1	21	1	18.3	1	0	0	1	2	1	1
8	1	22	1	33.7	3	1	1	0	2	1	1
9	1	20	1	20.2	1	0	0	0	2	2	2
10	1	23	1	34.3	3	0	0	1	3	1	1
11	1	24	1	20.3	1	1	0	1	1	1	1
12	1	18	1	29.4	2	1	1	1	2	1	1
13	2	19	1	24.4	1	0	0	0	3	2	2
14	2	22	1	23.2	1	0	0	1	3	2	3
15	1	18	1	18.2	1	0	0	0	3	1	1
16	2	27	2	27.7	2	1	0	1	1	2	3
17	1	20	1	16.5	1	1	0	0	3	1	1
18	1	18	1	23.5	1	1	0	0	2	1	2
19	1	22	1	20.3	1	1	0	0	2	2	2
20	1	21	1	28.0	2	1	1	0	2	1	2
21	1	23	1	23.1	1	1	1	0	2	1	3
24	1	19	1	23.4	1	0	0	0	2	1	1
25	1	18	1	19.3	1	0	0	1	3	1	1
26	1	23	1	22.9	1	0	0	0	3	1	1
27	1	20	1	19.1	1	0	0	0	3	1	1
28	1	22	1	25.8	2	0	0	0	1	1	2
30	1	23	1	16.4	1	0	0	0	2	1	1
31	2	22	1	26.9	2	1	0	0	3	1	2
33	2	21	1	21.8	1	1	1	0	3	2	2
34	1	21	1	24.8	1	0	0	0	3	1	3
35	2	28	2	26.1	2	1	0	1	2	1	1
37	1	20	1	21.3	1	1	0	0	2	1	2
38	2	27	2	23.6	1	1	0	0	2	1	3
39	2	19	1	22.4	1	1	1	0	3	2	1
41	2	31	2	34.3	3	0	0	0	2	1	2
43	1	23	1	20.0	1	0	1	0	2	1	2
44	2	19	1	24.8	1	0	0	0	2	2	1
45	2	19	1	27.7	2	0	0	0	1	1	2
46	1	21	1	19.5	1	0	0	0	2	1	1
47	1	21	1	33.5	3	0	0	0	3	1	2
48	1	19	1	21.7	1	0	0	0	2	1	1
49	1	19	1	18.8	1	1	1	0	2	1	1
50	1	23	1	24.9	1	0	0	0	2	1	1
52	1	25	1	22.9	1	1	0	0	3	2	1
53	1	20	1	19.1	1	0	0	0	2	1	1
54	1	23	1	21.0	1	1	0	0	3	1	3
55	2	23	1	19.7	1	0	0	0	2	1	1
58	1	20	1	19.0	1	1	1	0	2	1	1
59	1	23	1	22.5	1	0	0	0	3	2	2
60	1	19	1	21.7	1	1	1	0	2	2	1
61	1	19	1	20.5	1	0	0	1	3	2	2
62	1	24	1	27.9	2	0	1	0	2	1	1
64	2	20	1	26.1	2	0	0	0	2	2	2
65	1	24	1	24.3	1	1	0	0	3	1	1
66	1	24	1	21.2	1	0	0	0	3	2	2
67	1	22	1	25.1	2	1	1	0	2	1	2
69	2	21	1	31.4	3	1	1	0	2	1	1
71	1	22	1	20.9	1	1	0	0	3	1	1
72	1	19	1	22.5	1	0	0	0	2	1	1
73	1	20	1	21.6	1	0	0	0	3	2	2
75	1	20	1	21.2	1	0	0	0	2	1	1
76	1	18	1	22.5	1	1	0	0	3	1	1
77	1	19	1	20.2	1	0	0	0	2	1	1
78	1	19	1	16.6	1	1	1	0	3	2	1
79	1	23	1	19.5	1	0	1	0	3	1	2
80	1	28	2	20.8	1	0	0	1	2	1	1
81	2	22	1	20.1	1	1	0	0	3	2	2

82	1	22	1	28.3	2	0	0	0	3	1	1
83	1	23	1	27.0	2	0	0	0	2	1	2
84	1	19	1	18.3	1	0	0	0	3	1	2
85	1	22	1	21.7	1	1	0	0	3	1	2
89	1	18	1	29.4	2	0	0	1	1	1	1
90	1	21	1	21.9	1	0	0	1	3	1	2
91	2	22	1	24.2	1	0	1	0	3	1	3
92	1	22	1	32.4	3	0	1	0	2	1	1
93	1	18	1	22.8	1	0	0	0	2	1	1
94	1	18	1	33.6	3	1	1	0	2	1	1
96	1	20	1	23.8	1	0	0	0	3	2	1
97	1	21	1	21.1	1	0	0	1	1	1	2
98	2	22	1	20.6	1	0	1	1	3	1	1
99	1	21	1	21.5	1	1	0	0	3	2	2
102	2	21	1	20.8	1	1	0	0	3	2	2
103	2	25	1	27.1	2	0	0	0	2	1	2
104	1	20	1	22.7	1	1	1	0	2	2	1
105	1	19	1	17.1	1	0	0	0	2	1	2
106	1	18	1	20.8	1	0	1	0	3	1	2
107	1	21	1	27.6	2	1	1	0	1	1	1
108	2	24	1	24.4	1	1	1	0	3	1	1
109	1	23	1	30.1	3	0	0	0	3	1	1
110	2	21	1	25.9	2	1	0	0	1	1	2
111	2	23	1	29.4	2	0	0	0	1	1	1
112	1	18	1	19.7	1	0	0	0	1	1	1
114	1	20	1	22.6	1	0	1	0	3	1	2
115	2	21	1	25.1	2	0	0	0	3	2	3
117	1	18	1	21.4	1	0	0	0	2	1	2
119	1	22	1	22.0	1	0	0	0	2	2	1
120	1	21	1	24.2	1	0	0	0	2	1	1
121	1	23	1	21.8	1	0	0	0	1	2	2
123	1	21	1	25.0	1	0	0	0	3	1	2
124	1	24	1	20.7	1	1	0	0	3	1	1
125	1	19	1	17.8	1	0	0	1	3	1	2
126	2	23	1	33.1	3	0	0	0	3	1	2
127	2	23	1	29.8	2	1	0	0	3	1	2
129	2	21	1	19.2	1	0	1	1	2	1	3
131	2	18	1	22.2	1	1	1	0	2	1	2
132	1	23	1	33.8	3	1	1	0	3	1	1
133	1	22	1	23.6	1	0	0	1	2	1	1
134	1	21	1	23.5	1	0	1	0	3	1	2
135	2	21	1	25.0	2	1	0	0	1	2	1
136	2	23	1	30.4	3	1	0	1	2	1	1
138	2	19	1	18.8	1	0	0	0	3	1	1
140	2	23	1	23.6	1	1	1	0	2	1	1
141	1	19	1	26.8	2	0	0	1	3	1	1
142	1	31	2	17.5	1	0	0	0	2	1	1
143	1	24	1	19.9	1	1	1	1	3	1	2
145	1	21	1	24.1	1	0	1	0	1	1	3
146	1	24	1	20.4	1	1	1	0	3	1	1
148	2	27	2	20.0	1	1	1	0	3	2	1
149	1	24	1	29.4	2	0	0	0	2	1	1
150	1	24	1	15.4	1	0	0	1	3	2	1
151	1	18	1	26.0	2	1	0	0	3	1	1
152	1	19	1	24.9	1	0	0	0	3	1	1
153	1	20	1	18.2	1	1	1	0	2	1	2
154	1	18	1	21.0	1	1	1	1	2	2	3
155	2	30	2	34.7	3	1	0	0	2	1	1
156	2	22	1	25.2	2	0	0	0	3	1	2
157	1	18	1	28.9	2	0	0	0	1	1	2
159	2	18	1	35.8	3	1	0	0	3	1	2
161	1	18	1	24.8	1	0	0	0	2	1	1
163	1	21	1	26.3	2	0	0	0	3	2	1
165	1	23	1	21.0	1	0	0	0	3	2	2
166	1	25	1	27.5	2	0	0	0	2	1	2
167	1	25	1	19.2	1	0	1	0	3	1	1

168	1	24	1	25.4	2	0	0	0	3	1	1
169	2	26	2	24.8	1	1	1	0	2	1	1
170	2	31	2	22.5	1	1	1	0	2	2	1
174	2	21	1	27.8	2	0	0	0	3	2	2
176	1	22	1	23.5	1	1	0	0	3	2	1
177	2	20	1	29.4	2	0	0	0	1	2	2
178	1	18	1	27.1	2	1	1	0	3	1	1
180	2	22	1	21.7	1	1	0	0	2	1	2
183	1	21	1	42.4	3	0	1	0	3	1	1
184	1	26	2	20.2	1	0	0	1	2	1	1
185	1	18	1	32.7	3	1	0	1	3	1	3
186	1	22	1	17.8	1	1	1	0	2	1	1
187	1	19	1	21.8	1	0	1	0	2	1	1
188	1	21	1	32.3	3	0	0	0	2	1	2
189	1	18	1	20.2	1	1	1	0	2	1	2
190	2	20	1	30.5	3	1	0	0	2	1	2
193	2	24	1	33.6	3	0	0	0	2	2	2
195	1	21	1	18.6	1	0	1	0	2	1	2
196	1	23	1	19.5	1	0	1	0	2	1	2
197	1	22	1	20.0	1	0	0	0	2	1	3
198	1	22	1	20.8	1	0	0	0	2	1	1
199	2	28	2	27.7	2	0	0	0	1	2	1
200	1	20	1	19.5	1	0	0	0	2	1	2
202	1	18	1	23.8	1	0	0	0	1	1	1
203	2	18	1	20.5	1	1	0	1	1	2	3
204	1	22	1	20.6	1	0	1	0	3	1	1
205	1	22	1	21.7	1	1	1	0	2	2	2
206	1	20	1	20.9	1	0	0	1	2	1	2
208	1	20	1	19.4	1	0	0	0	3	1	2
209	1	23	1	23.6	1	0	0	0	3	2	2
210	1	25	1	19.5	1	0	0	1	3	1	3
213	1	21	1	23.2	1	0	0	0	3	2	1
214	1	20	1	21.9	1	0	0	0	3	1	1
215	1	26	2	23.2	1	0	0	0	3	1	1
216	2	24	1	24.6	1	1	1	0	2	2	3
217	1	24	1	25.3	2	0	1	0	2	1	1
218	2	22	1	31.4	3	1	1	1	3	2	2
220	1	22	1	17.2	1	0	0	1	2	1	1
221	1	19	1	21.3	1	0	0	0	3	1	3
222	1	23	1	23.9	1	0	0	0	3	1	1
223	2	23	1	24.4	1	0	1	0	2	1	1
224	2	19	1	28.4	2	0	0	0	3	2	1
225	2	31	2	30.1	3	0	0	0	2	2	2
227	2	22	1	20.5	1	0	0	1	2	1	2
228	1	21	1	25.7	2	0	0	0	3	1	3
229	1	19	1	18.4	1	1	0	0	3	1	2
230	1	21	1	30.9	3	0	0	1	3	1	2
231	1	20	1	19.9	1	0	0	0	3	1	1
232	1	20	1	22.0	1	1	1	0	2	1	1
233	1	24	1	24.0	1	1	1	1	2	1	3
235	2	26	2	23.4	1	0	0	1	2	1	2
236	1	18	1	16.1	1	0	0	0	2	1	2
238	1	26	2	25.5	2	1	0	0	3	1	1
239	1	26	2	33.0	3	0	0	1	2	1	1
240	2	21	1	39.0	3	0	0	0	3	2	1
241	1	21	1	27.3	2	0	0	0	3	1	2
242	1	21	1	20.8	1	1	0	1	3	1	3
243	1	24	1	21.3	1	1	1	0	3	1	2
244	1	22	1	22.8	1	1	0	0	2	1	2
245	1	18	1	17.4	1	0	0	0	3	1	1
246	1	22	1	36.7	3	0	1	0	2	1	1
247	1	19	1	23.0	1	0	0	0	2	1	1
248	1	21	1	23.1	1	0	0	0	2	1	1
249	2	24	1	25.2	2	1	1	0	3	2	2
251	1	19	1	19.5	1	0	0	0	3	1	2
252	1	24	1	28.4	2	0	0	0	3	1	1

254	1	23	1	17.7	1	1	0	0	3	1	2
255	1	20	1	26.2	2	0	0	0	3	2	1
256	2	20	1	22.4	1	1	0	0	2	2	1
258	1	19	1	32.0	3	1	1	0	2	1	1
259	1	22	1	19.4	1	0	0	0	2	1	2
260	1	20	1	27.8	2	0	0	0	1	1	1
264	1	26	2	23.2	1	0	0	0	3	1	2
265	2	20	1	22.9	1	1	0	0	2	1	3
267	1	21	1	17.7	1	0	1	0	3	1	1
268	1	21	1	20.6	1	1	0	1	2	1	3
269	1	20	1	27.1	2	1	1	0	3	1	2
270	1	22	1	17.9	1	0	0	0	2	2	1
271	1	23	1	39.4	3	1	0	1	2	2	3
272	2	22	1	24.3	1	0	0	0	3	2	3
273	2	22	1	18.8	1	1	1	0	2	2	2
274	2	23	1	19.0	1	1	1	0	1	2	2
275	1	23	1	21.6	1	1	0	1	3	1	3
277	1	21	1	24.2	1	0	0	0	2	1	2
278	2	18	1	18.9	1	0	0	0	3	1	3
279	1	20	1	19.3	1	0	1	1	1	1	1
280	1	20	1	22.2	1	1	0	0	3	1	1
281	1	19	1	23.7	1	0	1	0	3	1	2
282	1	18	1	18.5	1	0	0	0	1	1	1
284	2	19	1	21.5	1	0	0	0	3	1	1
285	2	19	1	27.8	2	0	0	0	3	2	2
286	1	19	1	19.7	1	1	0	0	3	1	1
288	1	18	1	21.2	1	0	1	0	2	1	1
289	1	22	1	27.9	2	0	0	0	1	1	2
291	1	19	1	20.2	1	0	0	0	2	1	1
293	1	21	1	23.7	1	0	0	0	2	1	1
294	1	22	1	26.8	2	0	0	0	1	1	2
295	1	22	1	25.7	2	0	0	0	3	1	1
296	2	20	1	18.6	1	0	0	0	1	2	1
297	1	18	1	27.3	2	1	0	0	2	2	2
298	1	20	1	23.2	1	0	0	0	2	1	1
299	2	21	1	18.9	1	0	0	0	2	2	1
301	1	19	1	24.7	1	1	1	0	2	1	3
302	1	21	1	17.2	1	0	0	0	2	1	1
303	2	20	1	24.9	1	0	0	0	2	2	2
304	1	19	1	20.4	1	0	0	0	3	1	2
305	2	35	2	30.9	3	0	1	0	1	1	1
306	2	30	2	28.4	2	0	1	0	3	1	2
307	1	18	1	20.7	1	0	0	1	1	1	1
308	1	18	1	18.7	1	0	0	0	3	1	2
311	1	26	2	28.0	2	1	0	0	2	1	2
312	1	18	1	21.6	1	0	0	1	3	1	1
313	1	19	1	17.2	1	0	0	0	1	1	1
314	1	19	1	21.3	1	0	0	0	2	1	2
315	2	21	1	24.0	1	1	1	0	2	1	1
316	1	31	2	29.3	2	1	1	0	2	1	2
317	2	27	2	36.4	3	0	0	0	3	2	1
319	1	22	1	26.1	2	0	1	0	2	2	1
321	2	19	1	19.6	1	0	1	0	2	1	3
323	2	20	1	22.4	1	0	0	0	1	2	2
327	1	20	1	23.7	1	1	0	0	3	2	1
331	1	18	1	22.2	1	0	0	1	2	1	2
332	2	19	1	21.4	1	0	0	1	1	2	3
333	1	19	1	23.4	1	0	0	0	3	1	1
334	1	21	1	21.0	1	1	0	0	2	1	1
335	1	20	1	23.0	1	0	1	0	1	1	1
336	2	21	1	24.9	1	0	0	0	3	1	3
337	1	18	1	22.5	1	0	0	0	3	1	1
338	1	19	1	19.5	1	1	0	1	3	1	3
339	2	21	1	22.2	1	0	1	0	3	1	1
342	1	21	1	20.3	1	0	1	0	2	1	2
345	1	20	1	25.0	1	0	1	0	2	1	3

346	2	19	1	23.4	1	0	1	0	1	1	2
347	2	23	1	29.1	2	1	0	0	3	2	3
350	1	23	1	23.0	1	0	0	0	2	1	1
351	1	21	1	18.7	1	0	0	0	2	1	1
354	1	18	1	34.6	3	0	0	0	3	1	1
358	2	21	1	20.2	1	0	0	1	2	1	2
359	1	25	1	20.8	1	1	0	0	3	2	1
360	1	22	1	20.1	1	0	0	0	3	1	2
361	1	20	1	18.7	1	0	0	0	1	1	2
362	1	21	1	24.1	1	0	0	0	3	1	2
363	1	20	1	26.9	2	1	1	0	1	2	1
364	1	19	1	19.1	1	0	0	0	3	1	1
365	1	23	1	22.7	1	0	0	0	2	1	2
366	2	21	1	24.7	1	0	0	0	2	2	2
367	1	25	1	26.3	2	1	1	0	1	1	1
370	1	28	2	23.9	1	0	0	1	3	1	1
371	1	20	1	23.8	1	0	0	0	2	1	1
372	1	20	1	22.2	1	0	0	0	2	1	2
373	1	20	1	24.4	1	0	0	1	2	1	1
374	1	20	1	25.3	2	0	0	0	3	2	1
376	1	20	1	23.1	1	1	0	1	2	2	2
377	1	21	1	22.5	1	1	1	0	2	2	3
378	2	18	1	24.1	1	1	1	0	3	1	2
380	1	18	1	19.5	1	1	1	0	2	1	2
381	1	18	1	26.5	2	1	0	1	3	1	2
382	1	42	2	24.1	1	0	0	1	3	2	3
383	1	23	1	20.5	1	0	0	1	3	2	2
385	1	21	1	24.4	1	1	1	1	2	1	3
386	1	21	1	19.6	1	0	0	0	2	1	1
387	1	22	1	20.5	1	1	0	0	3	2	2
388	1	20	1	19.7	1	1	0	0	2	1	2
389	1	23	1	19.7	1	0	0	1	2	1	1
390	1	19	1	21.0	1	0	1	0	1	1	1
392	1	19	1	19.5	1	1	0	0	3	1	1
393	1	25	1	24.8	1	0	0	1	2	1	1
394	2	22	1	28.4	2	0	1	1	1	1	1
395	1	23	1	22.4	1	1	0	0	3	1	2
396	1	18	1	24.2	1	1	1	0	3	2	2
398	1	19	1	27.4	2	1	0	0	3	1	2
399	2	20	1	20.9	1	0	0	0	3	1	1
400	2	18	1	25.7	2	0	0	0	3	2	2
401	1	23	1	27.5	2	0	0	0	3	1	1
402	2	26	2	23.5	1	1	1	1	3	1	3
403	2	21	1	28.4	2	0	0	0	2	2	2
404	1	22	1	26.3	2	0	0	1	3	1	1
405	1	20	1	18.8	1	1	0	0	2	1	1
406	2	24	1	20.2	1	0	0	0	2	1	1
408	1	21	1	20.6	1	0	0	0	3	2	3
409	1	20	1	22.3	1	0	0	0	2	1	1
411	2	23	1	27.1	2	0	0	0	2	2	2
415	1	22	1	25.4	2	0	0	1	3	1	1
417	1	24	1	22.9	1	0	0	0	2	1	2
418	1	21	1	21.2	1	0	0	0	1	1	1
419	1	19	1	22.6	1	0	0	0	3	2	1
421	1	18	1	23.2	1	0	0	0	3	1	2
422	1	20	1	20.4	1	0	0	0	2	1	1
423	1	20	1	27.5	2	0	1	0	3	1	2
424	1	19	1	25.0	1	1	0	0	1	1	1
425	1	21	1	21.1	1	0	0	0	3	2	1
426	1	21	1	27.9	2	1	1	0	1	1	2
428	2	18	1	28.4	2	1	1	1	2	2	3
429	1	18	1	16.9	1	0	0	0	3	2	2
431	1	19	1	16.8	1	1	0	0	1	1	1
433	1	23	1	21.1	1	0	0	1	3	1	3
434	1	28	2	25.8	2	0	0	0	1	1	3
436	1	24	1	25.2	2	1	0	0	3	1	3

438	1	21	1	19.3	1	0	0	0	3	1	2
439	2	24	1	29.4	2	1	1	0	3	1	3
440	1	19	1	17.2	1	1	1	1	2	1	2
441	1	22	1	20.5	1	1	0	0	2	1	3
442	1	19	1	22.6	1	0	0	0	2	2	2
443	1	28	2	22.1	1	1	0	1	3	2	2
444	1	19	1	23.0	1	0	0	0	3	1	1
445	1	19	1	25.3	2	0	0	0	3	2	3
446	1	23	1	19.4	1	0	0	0	3	1	1
448	2	21	1	23.1	1	0	0	0	2	1	1
450	2	25	1	26.6	2	1	1	0	1	1	2
452	2	23	1	22.0	1	0	0	0	3	1	1
453	1	22	1	22.0	1	0	0	0	3	1	2
454	1	24	1	41.5	3	0	1	0	2	1	1
456	1	20	1	21.9	1	0	0	0	3	1	2
457	1	21	1	24.1	1	0	0	1	3	1	1
458	1	21	1	22.4	1	1	0	1	3	1	1
460	1	23	1	27.6	2	0	0	0	1	1	1
461	1	22	1	18.3	1	1	1	1	3	1	1
464	1	20	1	20.7	1	0	0	0	3	2	3
465	1	19	1	18.8	1	1	1	0	3	2	1
466	2	21	1	26.3	2	0	0	0	3	1	1
467	1	21	1	22.0	1	0	1	0	3	1	1
469	2	21	1	28.4	2	0	1	1	3	1	2
470	1	19	1	20.0	1	0	0	0	3	2	1
471	1	21	1	22.3	1	0	1	1	3	1	1
472	1	20	1	22.7	1	1	1	0	3	1	2
473	2	21	1	23.1	1	0	0	0	2	1	1
475	1	18	1	18.3	1	1	1	0	3	2	3
478	1	18	1	20.2	1	0	0	0	3	1	2
479	1	22	1	18.2	1	0	0	0	3	1	1
481	2	22	1	20.6	1	0	0	1	3	2	1
482	2	30	2	28.4	2	1	0	1	2	1	1
484	1	19	1	19.2	1	0	0	0	3	2	1
485	2	18	1	23.4	1	0	0	0	1	2	1
486	2	20	1	21.8	1	1	0	0	3	1	1
487	2	20	1	27.2	2	1	0	0	1	1	2
489	2	19	1	19.0	1	1	1	0	3	2	2
490	2	21	1	25.1	2	1	1	0	2	1	2
491	1	19	1	20.3	1	1	0	0	3	1	2
492	1	20	1	17.2	1	1	1	0	2	1	1
493	1	20	1	20.3	1	0	0	0	3	1	1
494	2	18	1	31.4	3	1	1	0	3	2	2
495	1	19	1	19.1	1	0	0	0	3	1	1
496	1	20	1	22.3	1	0	0	0	3	2	2
497	1	22	1	22.2	1	1	1	0	3	1	2
498	2	25	1	22.2	1	0	0	0	1	1	3
499	1	22	1	18.1	1	0	0	1	3	1	2
500	1	18	1	21.8	1	1	0	0	2	1	1
501	1	19	1	20.7	1	1	0	1	3	1	1
502	1	21	1	22.4	1	0	0	1	1	1	2
504	2	22	1	25.6	2	0	0	0	3	1	1
505	1	18	1	24.0	1	0	0	0	3	1	1
506	1	19	1	27.3	2	0	0	1	2	2	2
508	1	22	1	23.4	1	0	0	0	3	1	2
509	1	24	1	23.1	1	0	0	0	1	1	1
510	1	27	2	18.4	1	0	0	1	1	1	1
511	1	21	1	22.3	1	1	1	0	2	1	1
512	1	19	1	22.9	1	0	1	1	3	1	2
513	2	32	2	32.7	3	0	0	0	3	1	2
515	1	18	1	21.1	1	0	0	1	2	1	3
516	2	18	1	21.1	1	1	0	1	1	1	2
517	1	26	2	20.1	1	1	0	0	3	1	1
518	1	18	1	24.1	1	1	0	0	3	1	1
519	2	20	1	24.5	1	1	0	1	3	2	3
522	1	20	1	27.0	2	0	0	0	3	1	1

524	2	24	1	21.6	1	0	0	0	3	2	1
525	1	24	1	21.3	1	1	1	0	3	1	2
526	1	25	1	28.3	2	0	0	0	2	1	1
527	1	23	1	20.7	1	0	0	0	3	1	2
528	1	19	1	22.5	1	0	0	0	2	1	3
529	2	22	1	21.4	1	0	0	0	2	1	1
530	2	18	1	21.1	1	1	1	0	2	1	2
531	1	20	1	28.8	2	0	0	1	3	2	3
532	2	19	1	21.3	1	0	0	0	2	2	2
536	1	18	1	20.7	1	0	0	0	2	1	1
537	1	21	1	21.3	1	0	0	0	2	1	2
538	2	19	1	18.4	1	0	0	1	2	2	3
540	2	22	1	19.4	1	0	0	0	2	2	3
541	1	20	1	19.5	1	1	0	0	3	2	2
543	1	21	1	20.7	1	1	1	0	3	1	2
545	1	18	1	19.4	1	0	0	0	3	1	1
547	1	25	1	23.1	1	0	0	0	2	1	2
548	2	27	2	24.3	1	1	1	0	2	2	2
549	1	33	2	33.3	3	1	0	0	3	1	1
551	2	24	1	22.5	1	0	1	1	3	1	3
553	1	18	1	25.7	2	0	0	0	3	2	3
554	1	23	1	21.3	1	0	0	0	3	1	1
555	2	19	1	20.3	1	0	0	0	3	1	2
557	1	27	2	17.2	1	0	0	0	3	2	1
558	1	22	1	18.1	1	0	0	0	3	1	1
560	1	18	1	34.7	3	0	1	0	3	1	1
561	1	20	1	25.8	2	0	1	0	2	1	2
562	1	21	1	33.3	3	1	1	0	2	1	1
563	2	24	1	19.6	1	0	1	0	3	1	2
564	1	25	1	21.9	1	1	0	0	2	1	2
565	2	21	1	21.3	1	0	0	1	3	2	2
567	1	20	1	23.9	1	0	0	1	2	1	1
568	2	18	1	25.1	2	1	1	0	3	2	3
569	1	21	1	21.1	1	0	0	0	1	1	1
571	1	22	1	17.1	1	0	0	0	2	1	2
573	1	21	1	16.0	1	1	0	0	3	1	2
576	1	19	1	26.5	2	1	0	1	3	1	2
578	1	20	1	20.1	1	0	0	1	2	2	2
579	1	19	1	26.0	2	0	0	0	2	1	2
580	1	20	1	25.2	2	1	1	1	3	1	2
581	2	21	1	24.6	1	0	0	0	1	1	1
582	1	21	1	18.4	1	1	1	0	3	1	1
583	1	18	1	31.3	3	0	0	0	3	1	2
586	2	26	2	20.0	1	1	1	0	2	1	2
589	1	21	1	20.8	1	0	0	0	2	1	1
590	1	19	1	19.7	1	0	0	0	3	1	1
591	1	20	1	34.2	3	0	0	1	3	2	1
592	2	22	1	24.5	1	0	0	0	2	2	2
593	1	21	1	19.5	1	0	0	0	3	1	1
594	1	19	1	32.0	3	0	0	0	3	1	2
595	1	26	2	22.2	1	1	0	0	3	1	1
597	1	19	1	26.8	2	0	0	0	2	1	2
598	1	23	1	18.4	1	0	0	0	2	1	3
599	1	20	1	22.6	1	0	0	0	3	2	2
600	1	25	1	21.5	1	1	1	0	1	1	1
602	1	22	1	26.6	2	0	0	0	2	1	1
603	1	25	1	24.4	1	0	0	0	2	1	1
604	2	31	2	22.9	1	0	0	1	3	2	2
605	2	25	1	31.1	3	1	1	0	2	1	2
608	1	20	1	16.6	1	0	1	0	3	2	1
609	1	31	2	25.5	2	0	0	0	3	1	1
610	1	19	1	28.0	2	0	0	1	1	1	3
611	1	18	1	19.7	1	1	0	0	2	2	1
612	2	18	1	24.7	1	0	0	0	2	1	1
613	1	18	1	24.5	1	0	0	0	3	2	1
614	1	19	1	25.0	1	0	0	0	3	2	1

615	1	20	1	28.3	2	0	0	0	3	1	1
616	1	19	1	25.7	2	0	0	0	3	1	2
617	1	18	1	23.6	1	0	0	1	3	1	2
618	1	23	1	17.0	1	0	1	0	1	1	2
619	2	21	1	30.4	3	1	1	0	3	1	2
620	2	21	1	22.6	1	0	1	0	3	1	1
622	2	21	1	29.7	2	0	0	1	3	2	3
623	2	18	1	26.4	2	1	1	1	3	1	3
624	1	22	1	23.9	1	1	1	0	3	1	2
625	1	32	2	27.5	2	0	0	0	1	1	1
626	2	22	1	21.7	1	0	0	1	3	1	2
627	1	19	1	21.0	1	0	0	0	2	1	1
628	1	18	1	15.8	1	0	0	0	2	1	1
629	2	20	1	23.1	1	1	0	0	2	2	2
630	2	19	1	25.4	2	0	0	0	3	1	2
631	1	18	1	25.8	2	0	0	0	3	2	2
632	1	26	2	19.0	1	0	0	0	2	1	1
633	1	21	1	24.3	1	1	1	0	2	1	3
635	1	22	1	23.9	1	0	0	0	3	1	1
636	1	21	1	26.3	2	0	1	0	2	1	1
639	2	29	2	26.6	2	0	0	1	3	1	3
640	1	22	1	18.4	1	0	0	0	2	1	1
641	1	22	1	31.2	3	0	0	0	2	1	2
642	1	18	1	20.8	1	0	0	1	3	1	1
643	2	21	1	33.1	3	0	0	0	3	1	2
644	1	19	1	16.3	1	0	0	1	2	1	2
645	2	20	1	20.0	1	1	0	1	2	2	2
646	1	18	1	24.4	1	0	1	0	2	1	3
647	2	25	1	22.2	1	0	1	1	2	2	3
648	1	18	1	21.3	1	0	0	1	3	1	2
649	1	27	2	33.9	3	1	1	0	2	1	1
650	1	22	1	32.0	3	0	0	0	3	1	1
652	1	22	1	19.5	1	0	0	1	3	1	2
653	1	19	1	23.3	1	0	0	0	1	1	2
654	1	22	1	33.1	3	0	0	0	2	1	1
655	1	18	1	32.7	3	0	0	0	3	1	1
657	1	20	1	29.4	2	0	0	0	2	1	3
658	2	20	1	26.4	2	0	0	0	2	1	2
659	2	27	2	26.0	2	1	0	0	2	1	1
660	1	23	1	25.7	2	1	1	1	3	1	1
662	1	23	1	19.7	1	1	1	1	3	1	1
663	2	21	1	26.0	2	1	1	1	3	1	3
664	1	19	1	34.0	3	0	0	0	2	1	1
665	1	18	1	19.9	1	0	0	0	3	1	1
666	2	19	1	21.9	1	1	1	0	3	2	2
667	1	26	2	24.2	1	1	0	1	3	2	1
668	2	18	1	25.1	2	1	1	0	2	1	1
669	1	18	1	21.3	1	0	1	0	2	1	2
670	2	22	1	27.3	2	1	1	0	3	2	2
671	1	37	2	35.6	3	0	0	0	1	1	1
672	1	21	1	17.0	1	1	0	1	1	1	1
673	1	21	1	26.8	2	1	0	1	1	1	3
674	2	21	1	24.1	1	0	1	0	1	1	3
675	1	27	2	27.3	2	1	0	0	3	1	1
676	1	20	1	20.2	1	0	0	0	3	1	2
677	1	27	2	20.3	1	1	1	0	3	2	1
678	2	22	1	38.4	3	0	1	0	2	1	1
679	1	20	1	38.4	3	1	1	0	2	1	1
681	1	19	1	17.0	1	1	0	1	3	1	2
682	1	22	1	18.2	1	1	1	0	3	2	1
683	1	23	1	23.7	1	1	1	0	1	1	1
684	1	31	2	29.2	2	0	0	0	3	1	1
685	1	24	1	32.0	3	0	0	0	2	2	1
686	1	26	2	36.4	3	1	1	1	3	1	1
687	1	23	1	25.8	2	1	1	0	3	1	1
688	1	24	1	17.6	1	0	1	0	1	1	1

689	2	23	1	28.9	2	0	0	0	3	1	3
692	1	28	2	35.8	3	0	0	0	3	1	1
693	1	21	1	20.1	1	1	1	0	2	1	1
694	1	20	1	26.0	2	1	0	0	3	1	1
696	1	20	1	22.6	1	1	1	0	3	1	1
697	1	25	1	23.2	1	0	0	0	2	1	1
698	1	25	1	28.2	2	1	1	1	3	1	2
699	1	40	2	21.3	1	1	0	0	2	1	1
700	2	21	1	28.4	2	1	1	1	3	1	2
701	1	23	1	30.8	3	0	0	0	3	1	1
703	1	18	1	20.4	1	0	0	1	3	2	3
704	1	20	1	18.1	1	1	0	0	3	2	1
705	1	22	1	19.5	1	0	0	0	3	1	1
706	1	20	1	23.8	1	1	1	1	2	1	2
708	1	24	1	22.4	1	0	1	1	1	2	3
709	1	20	1	24.4	1	1	1	0	3	1	1
710	1	18	1	23.8	1	0	0	0	2	2	3
711	1	19	1	19.2	1	0	0	0	3	2	2
712	1	23	1	22.8	1	1	0	0	3	2	2
714	1	20	1	18.6	1	1	1	0	2	1	1
715	1	27	2	34.9	3	0	1	0	2	1	1
716	2	19	1	27.1	2	0	0	0	3	2	2
717	2	21	1	23.8	1	0	0	0	3	1	3
718	1	19	1	22.4	1	0	0	0	3	1	1
719	1	19	1	21.9	1	0	0	0	1	2	1
721	2	18	1	22.0	1	1	1	0	3	1	3
722	1	18	1	20.4	1	0	0	0	3	1	1
723	1	19	1	22.0	1	1	1	0	2	1	1
726	1	18	1	25.8	2	1	0	0	3	1	1
727	1	18	1	19.5	1	1	1	0	2	1	3
728	1	19	1	19.6	1	1	0	0	2	1	1
730	2	24	1	29.7	2	1	1	1	2	2	3
733	1	18	1	19.8	1	0	0	0	2	1	2
735	2	18	1	18.3	1	1	1	0	2	1	2
736	2	18	1	25.9	2	0	0	0	1	2	2
738	1	18	1	21.5	1	0	0	1	3	1	2
739	2	26	2	33.2	3	0	0	0	2	1	1
740	1	19	1	21.4	1	0	0	1	2	1	3
741	1	19	1	20.3	1	0	0	1	2	1	2
743	2	22	1	20.7	1	1	0	0	3	1	1
744	2	22	1	26.1	2	1	1	0	2	1	2
746	2	23	1	20.1	1	0	0	1	3	2	2
747	1	18	1	17.6	1	0	0	0	3	1	1
749	1	18	1	29.8	2	0	1	0	3	1	1
750	2	20	1	18.3	1	0	0	1	3	2	2
752	1	18	1	19.3	1	0	0	1	2	2	1
753	2	20	1	25.7	2	1	1	0	2	1	2
754	1	20	1	21.3	1	1	0	1	2	1	3
755	2	18	1	21.6	1	0	0	0	2	2	3
757	2	19	1	23.1	1	1	1	0	3	2	1
758	2	21	1	29.7	2	1	0	1	1	2	2
759	2	19	1	21.0	1	0	0	1	2	1	2
760	1	19	1	18.7	1	1	0	1	2	1	2
764	1	18	1	18.7	1	1	0	1	3	2	2
765	1	18	1	21.2	1	0	0	0	2	1	1
766	2	19	1	21.5	1	0	0	0	1	1	2
767	1	20	1	24.9	1	0	0	0	2	1	1
768	1	22	1	23.1	1	1	0	0	3	2	2
769	1	26	2	24.1	1	1	1	0	2	1	2
770	1	20	1	23.7	1	0	0	0	3	2	1
771	1	22	1	41.5	3	0	0	1	3	2	1
772	1	21	1	18.2	1	0	0	0	3	2	1
773	1	20	1	21.2	1	0	0	1	3	1	1
774	1	21	1	20.1	1	0	0	0	3	2	1
775	1	24	1	20.6	1	1	0	0	3	2	2
776	1	23	1	20.4	1	0	0	0	1	1	1

777	2	21	1	31.1	3	0	1	0	3	1	2
778	2	18	1	22.6	1	1	1	0	3	2	2
779	1	18	1	19.2	1	1	0	0	2	1	1
780	1	21	1	18.1	1	0	0	0	3	2	1
781	2	21	1	28.0	2	1	1	0	3	1	2
783	1	19	1	21.9	1	0	0	0	3	2	1
784	1	20	1	19.4	1	0	0	0	3	2	2
785	2	41	2	26.8	2	0	0	1	3	1	3
786	2	22	1	23.1	1	0	0	0	2	1	2
787	2	19	1	20.1	1	0	0	0	3	1	1
790	1	20	1	22.1	1	0	0	0	2	1	2
791	1	18	1	22.1	1	0	0	0	2	1	1
792	1	19	1	21.7	1	0	0	0	2	1	1
793	1	23	1	18.7	1	0	0	0	3	1	2
797	1	19	1	19.4	1	0	0	0	1	1	2
798	2	24	1	27.7	2	0	1	0	2	1	1
800	1	24	1	23.5	1	0	1	1	3	2	1
801	1	18	1	21.7	1	0	0	1	3	2	1
803	1	31	2	24.0	1	1	1	1	3	1	2
804	1	18	1	20.2	1	0	1	0	3	1	2
805	1	28	2	18.7	1	0	0	0	2	1	2
806	2	18	1	22.2	1	0	0	0	3	1	1
808	1	20	1	20.1	1	0	0	1	2	1	2
809	1	20	1	23.4	1	0	0	0	2	1	1
810	1	19	1	21.5	1	0	0	0	3	1	1
811	1	19	1	19.8	1	0	0	0	1	1	1
813	1	21	1	19.1	1	1	0	0	3	1	1
814	2	19	1	24.7	1	1	0	0	3	2	1
815	2	20	1	26.3	2	1	1	0	1	1	1
817	1	20	1	20.3	1	0	0	0	1	1	1
818	2	23	1	17.0	1	1	1	0	2	1	1
820	1	18	1	19.0	1	0	0	0	3	2	3
821	1	21	1	18.3	1	0	0	0	3	2	2
824	1	20	1	22.5	1	0	0	0	3	1	1
825	2	25	1	20.9	1	1	1	0	2	1	2
826	2	19	1	25.7	2	0	0	0	2	2	2
827	1	18	1	19.3	1	0	0	1	1	1	2
829	1	22	1	35.1	3	0	0	0	3	2	1
830	1	30	2	25.7	2	0	0	0	3	1	1
831	1	20	1	27.1	2	0	0	0	2	1	1
833	2	19	1	27.5	2	0	0	0	2	1	1
834	2	18	1	23.4	1	1	1	0	2	1	1
835	2	20	1	25.7	2	0	0	0	3	2	3
837	2	22	1	18.4	1	1	1	0	3	1	1
838	2	31	2	20.4	1	1	0	0	3	2	1
839	2	24	1	26.7	2	0	0	0	1	1	3
840	1	21	1	31.9	3	0	0	0	3	1	1
841	2	24	1	22.5	1	0	1	0	2	2	2
842	1	18	1	35.3	3	0	0	0	2	1	1
844	2	23	1	24.2	1	0	0	0	3	2	2
845	1	20	1	21.6	1	1	1	0	2	1	1
846	1	21	1	21.9	1	0	1	0	3	1	1
847	2	25	1	23.2	1	1	1	1	2	1	2
848	1	29	2	20.8	1	1	0	0	2	1	2
850	2	26	2	26.9	2	0	1	0	2	1	2
851	1	19	1	18.4	1	0	0	0	3	1	2
852	1	24	1	27.3	2	0	0	0	1	1	1
853	1	23	1	24.5	1	0	0	0	2	1	2
854	2	29	2	22.5	1	0	0	0	3	2	1
856	1	20	1	28.0	2	1	0	0	3	1	2
857	2	21	1	20.8	1	0	0	1	2	1	2
858	1	18	1	21.6	1	0	0	0	3	2	1
861	1	20	1	20.2	1	0	0	1	3	1	1
862	1	29	2	25.7	2	0	0	0	2	1	1
863	2	20	1	38.0	3	0	0	0	2	1	1
864	1	24	1	33.8	3	0	1	0	2	1	3

865	2	27	2	26.5	2	1	1	0	3	1	2
866	2	23	1	22.5	1	0	0	0	1	1	2
867	1	18	1	22.6	1	1	0	0	1	1	1
868	2	31	2	27.5	2	0	0	0	3	1	3
869	2	18	1	16.7	1	0	0	0	3	2	2
870	2	22	1	31.5	3	1	0	0	1	1	1
872	1	18	1	24.4	1	1	0	0	3	1	1
873	2	27	2	23.7	1	0	0	0	3	2	2
874	1	18	1	47.3	3	1	0	0	3	1	1
875	2	19	1	16.2	1	0	0	0	2	2	1
876	1	22	1	21.2	1	0	0	0	3	2	2
877	1	18	1	21.7	1	0	0	1	3	1	1
878	1	23	1	36.3	3	0	0	0	2	1	1
879	1	18	1	16.6	1	1	0	0	2	1	2
880	2	19	1	29.8	2	0	0	0	2	2	2
881	1	25	1	17.8	1	0	0	0	2	1	1
882	1	20	1	19.4	1	0	0	0	2	2	2
884	1	18	1	22.9	1	0	0	0	2	1	2
885	1	21	1	20.6	1	0	0	1	2	1	1
886	1	20	1	20.7	1	0	1	0	3	1	3
887	2	19	1	22.0	1	0	0	1	1	2	1
889	1	20	1	19.0	1	0	0	0	3	1	1
890	2	22	1	19.9	1	0	0	0	2	1	1
891	1	25	1	21.9	1	0	0	0	3	1	1
892	2	29	2	30.9	3	0	0	1	2	1	3
894	1	21	1	26.7	2	0	0	0	2	1	1
895	2	20	1	18.3	1	0	0	0	1	2	1
896	2	22	1	25.7	2	0	0	0	1	1	2
898	1	19	1	24.3	1	0	0	0	3	1	2
900	2	20	1	22.0	1	0	0	0	1	2	1
902	2	24	1	33.0	3	1	0	0	2	2	3
903	2	20	1	23.1	1	0	0	0	3	1	3
904	1	19	1	24.0	1	0	0	0	2	1	1
905	1	22	1	20.8	1	1	0	0	3	1	2
906	1	20	1	16.2	1	0	0	1	2	1	1
907	2	26	2	31.7	3	0	0	1	2	1	3
908	2	30	2	27.1	2	0	0	0	2	2	3
909	1	18	1	28.1	2	0	0	0	2	1	1
910	2	21	1	25.1	2	0	0	0	3	1	1
912	2	20	1	24.6	1	0	0	0	3	1	2
914	1	18	1	21.8	1	0	0	0	2	1	1
915	1	19	1	28.4	2	1	1	0	3	1	2
916	1	23	1	19.0	1	0	0	0	3	2	2
919	1	19	1	22.8	1	0	0	1	2	1	1
920	2	20	1	18.5	1	1	0	0	3	2	1
921	1	21	1	15.8	1	0	0	0	2	1	1
922	2	21	1	36.0	3	1	1	1	1	2	2
923	1	21	1	23.2	1	1	0	0	2	1	2
925	1	22	1	22.0	1	1	0	0	2	1	1
926	1	21	1	25.3	2	0	0	0	3	1	1
927	1	53	2	31.6	3	0	0	1	2	2	2
928	1	21	1	35.5	3	1	1	0	1	1	3
930	2	20	1	32.1	3	0	0	1	3	1	3
931	1	20	1	23.3	1	0	1	0	2	1	3
932	1	20	1	35.1	3	0	0	0	3	1	2
934	1	19	1	37.1	3	0	0	0	2	1	1
935	2	20	1	20.3	1	1	0	1	3	1	3
936	2	20	1	33.1	3	1	0	0	3	1	1
938	2	22	1	23.7	1	0	0	0	3	1	1
939	1	24	1	28.5	2	0	1	0	2	1	3
940	1	22	1	19.2	1	0	0	1	3	2	3
944	1	21	1	23.8	1	0	0	1	1	1	3
945	1	18	1	19.6	1	0	0	1	3	1	2
946	1	29	2	20.2	1	0	1	0	1	1	1
947	1	19	1	26.9	2	0	1	0	2	1	1
948	1	18	1	23.8	1	0	0	0	3	2	2

949	1	23	1	19.4	1	1	1	0	3	1	2
950	2	31	2	27.0	2	1	0	0	3	1	2
951	2	20	1	25.2	2	0	0	1	3	1	3
952	1	24	1	24.2	1	0	0	1	3	2	3
953	1	20	1	19.1	1	1	1	0	3	1	1
954	2	19	1	27.0	2	0	1	0	3	1	1
955	1	23	1	29.4	2	0	0	1	3	2	1
957	1	22	1	20.4	1	0	0	0	1	1	1
959	1	23	1	20.5	1	0	0	1	1	1	3
960	1	25	1	25.3	2	1	1	0	3	2	1
961	1	21	1	21.0	1	1	0	0	3	1	3
964	1	27	2	23.0	1	1	0	1	3	1	1
965	1	22	1	18.4	1	0	0	0	3	1	1
966	1	19	1	25.4	2	1	0	0	3	1	1
967	1	29	2	25.5	2	0	1	1	1	1	1
968	1	22	1	27.6	2	1	1	0	3	1	1
969	2	21	1	31.1	3	0	0	0	3	1	2
970	1	18	1	18.1	1	0	0	0	2	1	2
971	2	20	1	18.4	1	0	0	0	3	2	1
974	2	20	1	26.4	2	0	0	1	3	2	3
976	2	18	1	15.4	1	0	0	0	3	2	1
977	1	19	1	27.0	2	0	0	0	2	1	1
978	2	19	1	25.0	2	0	0	0	1	1	2
981	2	25	1	31.6	3	1	1	0	2	1	1
983	1	23	1	19.5	1	0	0	0	2	2	1
984	1	18	1	27.3	2	0	0	0	3	1	1
985	1	18	1	26.3	2	0	0	0	2	2	2
986	1	20	1	29.4	2	0	0	0	2	1	1
987	2	20	1	28.7	2	0	0	0	3	2	2
992	2	18	1	21.7	1	0	1	0	1	1	2
995	1	20	1	18.4	1	0	0	0	2	1	2
997	2	43	2	40.4	3	0	0	0	2	1	1
998	1	30	2	36.0	3	0	0	1	1	1	3
999	2	30	2	31.5	3	0	0	0	1	2	1
1000	1	24	1	24.2	1	1	0	1	3	1	2
1001	2	33	2	22.5	1	0	0	0	2	2	1
1002	2	30	2	24.3	1	1	1	0	2	1	2
1003	1	19	1	16.8	1	0	1	0	2	1	1
1004	2	20	1	23.6	1	0	0	0	2	2	2
1005	2	29	2	22.0	1	0	0	0	3	1	2
1006	2	32	2	32.1	3	0	0	0	2	1	1
1007	2	35	2	21.9	1	0	0	0	2	1	2
1008	1	18	1	32.7	3	0	0	0	3	1	1
1009	1	21	1	21.3	1	0	1	0	3	1	2
1011	1	28	2	20.2	1	1	0	0	3	2	3
1012	2	19	1	34.7	3	0	0	0	2	1	2
1013	2	46	2	36.6	3	0	0	0	1	2	1
1014	1	18	1	22.6	1	1	1	0	3	1	2
1015	1	27	2	17.4	1	0	0	0	3	1	1
1016	1	25	1	24.7	1	1	1	0	2	1	1
1017	2	18	1	18.5	1	0	0	0	3	1	1
1019	2	41	2	21.0	1	0	0	0	2	1	1
1020	1	27	2	28.4	2	0	0	0	3	1	1
1021	2	22	1	23.0	1	1	1	0	3	2	1
1022	1	18	1	17.3	1	0	0	1	3	2	1
1023	1	37	2	29.9	2	0	0	1	2	2	3
1024	1	31	2	20.8	1	0	0	0	2	1	2
1026	2	18	1	32.8	3	0	0	0	2	1	2
1027	1	18	1	27.7	2	0	0	0	3	1	2
1028	2	23	1	25.6	2	1	1	0	3	2	3
1029	1	33	2	22.1	1	1	1	0	1	1	2
1030	2	21	1	20.6	1	1	0	0	3	2	2
1031	1	19	1	19.5	1	0	0	0	2	1	1
1032	2	19	1	23.3	1	0	0	0	3	2	2
1033	2	34	2	27.4	2	1	1	1	3	2	2
1034	1	27	2	34.2	3	1	1	0	3	1	1

1036	2	25	1	28.4	2	1	0	0	3	2	2
1037	1	23	1	27.7	2	0	0	0	3	1	1
1038	2	26	2	20.9	1	1	0	0	3	1	1
1039	2	20	1	39.2	3	1	1	0	3	1	2
1040	2	36	2	34.3	3	0	1	1	3	2	2
1041	1	32	2	28.3	2	1	0	0	3	1	1
1042	1	25	1	25.6	2	0	0	0	3	1	1
1043	1	20	1	18.5	1	1	0	0	3	2	2
1045	2	18	1	18.5	1	0	0	0	2	1	2
1046	1	22	1	20.0	1	0	0	0	3	1	2
1048	2	40	2	29.7	2	0	0	0	2	1	2
1049	1	22	1	18.4	1	0	0	1	2	2	1
1051	2	20	1	23.7	1	1	0	0	3	1	1
1052	2	21	1	26.3	2	0	0	0	3	1	2
1053	1	19	1	33.1	3	1	1	0	2	1	1
1054	1	20	1	22.7	1	0	0	0	3	1	1
1055	1	21	1	20.6	1	0	0	0	3	1	1
1056	1	22	1	30.9	3	1	1	0	3	1	1
1061	1	20	1	17.7	1	0	0	0	3	1	3
1062	1	20	1	33.2	3	0	0	0	3	1	3
1064	1	34	2	25.4	2	1	0	0	3	1	3
1065	2	26	2	21.8	1	0	0	1	2	1	3
1067	1	22	1	19.4	1	0	0	0	2	2	1
1068	2	25	1	27.4	2	1	0	0	3	1	1
1069	1	20	1	25.6	2	0	0	0	2	1	1
1070	1	20	1	20.6	1	0	0	0	3	1	2
1071	2	20	1	27.5	2	0	0	0	1	2	2
1072	1	21	1	29.4	2	0	0	0	2	1	1
1073	2	21	1	30.2	3	1	0	0	3	1	2
1074	2	18	1	19.6	1	0	0	0	2	1	2
1077	2	18	1	24.2	1	0	0	1	3	1	1
1078	1	21	1	21.5	1	1	1	1	2	1	1
1080	1	20	1	27.9	2	0	0	0	2	1	1
1081	1	19	1	27.2	2	0	0	0	2	1	2
1082	1	21	1	18.6	1	1	1	0	3	1	1
1083	1	21	1	29.8	2	0	0	0	2	1	1
1086	1	22	1	24.0	1	1	0	0	2	1	2
1087	2	30	2	17.2	1	1	1	0	2	1	1
1088	2	21	1	24.6	1	1	1	0	3	1	2
1089	1	21	1	22.0	1	0	1	0	3	2	2
1092	1	19	1	22.0	1	1	0	0	3	2	1
1093	1	21	1	25.7	2	0	0	0	2	1	3
1094	1	21	1	21.7	1	0	0	0	3	2	1
1095	1	21	1	20.1	1	1	1	0	3	2	1
1097	2	20	1	27.2	2	0	1	0	2	1	1
1098	2	23	1	22.5	1	0	0	0	3	2	3
1099	1	18	1	21.1	1	0	0	1	2	1	2
1100	1	20	1	17.6	1	0	0	0	3	1	2
1102	1	21	1	28.3	2	0	0	0	2	1	1
1103	2	20	1	16.7	1	0	0	0	2	2	1
1104	1	19	1	18.6	1	0	0	0	3	1	1
1105	1	21	1	22.7	1	1	1	0	3	2	1
1107	1	23	1	29.1	2	0	0	1	2	1	3
1108	1	22	1	21.5	1	0	0	0	2	1	1
1109	1	22	1	22.1	1	0	0	1	2	1	2
1110	1	19	1	25.6	2	1	0	1	1	2	1
1111	2	29	2	23.9	1	1	0	1	3	2	3
1112	1	19	1	22.7	1	0	0	0	3	1	1
1113	1	22	1	25.2	2	1	0	0	2	1	1
1114	1	19	1	24.9	1	1	0	0	2	1	1
1115	2	18	1	18.2	1	0	0	0	3	2	2
1116	1	23	1	21.0	1	1	1	0	3	1	1
1118	2	19	1	22.2	1	0	0	1	2	1	1
1120	1	24	1	21.0	1	0	0	0	3	1	1
1122	1	32	2	27.6	2	0	0	0	3	1	1
1123	1	18	1	17.7	1	1	0	0	3	1	1

1124	2	25	1	28.6	2	0	0	0	1	1	1
1125	1	26	2	19.3	1	1	1	0	2	2	2
1126	1	22	1	24.1	1	1	0	0	3	1	1
1128	1	22	1	24.2	1	0	0	0	2	1	1
1132	1	24	1	32.4	3	1	0	0	1	1	3
1133	1	23	1	32.4	3	1	1	0	2	1	1
1134	2	23	1	23.0	1	1	1	0	1	1	1
1135	2	27	2	24.2	1	1	1	0	1	1	2
1136	1	18	1	26.3	2	0	0	0	2	1	1
1138	2	22	1	21.6	1	1	1	0	2	1	2
1139	1	24	1	22.0	1	1	0	0	3	1	2
1141	1	25	1	31.1	3	0	0	0	2	1	2
1142	1	20	1	21.8	1	0	0	0	2	1	1
1143	2	19	1	17.9	1	1	0	0	3	1	3
1144	1	35	2	50.4	3	0	0	0	1	1	2
1145	1	23	1	25.6	2	0	0	0	3	2	1
1146	1	22	1	19.6	1	0	0	1	2	1	1
1147	2	25	1	35.9	3	0	1	1	3	1	2
1149	1	22	1	23.0	1	1	0	0	2	1	1
1150	1	21	1	26.3	2	1	0	0	1	1	3
1151	1	26	2	29.3	2	1	0	0	3	1	1
1152	1	19	1	22.8	1	0	0	0	3	2	1
1153	1	22	1	21.8	1	0	1	0	2	1	1
1154	1	20	1	21.7	1	0	0	0	3	1	1
1155	1	20	1	26.4	2	0	0	0	3	2	1
1156	1	22	1	22.5	1	1	1	0	3	1	1
1157	1	19	1	24.3	1	0	0	1	3	1	1
1158	2	22	1	25.7	2	0	1	0	3	1	3
1159	1	22	1	20.4	1	0	0	0	3	1	2
1161	1	19	1	18.2	1	0	0	0	3	1	1
1162	1	24	1	25.7	2	1	1	1	2	1	2
1165	2	18	1	21.8	1	1	1	1	3	1	2
1166	1	18	1	24.8	1	0	0	0	3	1	1
1169	1	21	1	21.5	1	0	0	0	3	1	1
1170	2	21	1	23.6	1	0	0	1	3	2	3
1171	1	20	1	22.5	1	1	0	0	3	1	1
1173	1	18	1	18.8	1	0	0	0	2	1	2
1174	1	19	1	23.7	1	0	0	0	2	1	1
1175	1	19	1	24.4	1	1	0	0	3	1	1
1177	1	23	1	19.9	1	0	0	0	3	1	1
1178	1	21	1	17.2	1	0	0	0	3	1	1
1179	1	19	1	19.6	1	1	1	0	3	2	2
1180	1	22	1	25.0	1	1	1	0	2	1	1
1181	1	21	1	19.8	1	1	1	0	2	1	2
1182	1	26	2	24.3	1	0	0	0	2	1	1
1183	1	25	1	25.0	1	0	0	0	3	1	1
1186	1	22	1	22.0	1	0	0	0	3	1	1
1187	1	22	1	24.2	1	0	0	0	3	1	1
1188	1	20	1	17.3	1	0	0	1	3	1	2
1189	2	21	1	16.1	1	0	0	0	3	1	1
1191	2	19	1	34.1	3	1	0	0	2	1	2
1193	1	20	1	18.8	1	1	0	0	2	1	3
1194	1	22	1	23.9	1	1	0	0	3	1	2
1195	2	27	2	24.1	1	1	0	0	3	1	2
1196	1	20	1	20.2	1	0	0	0	3	2	1
1197	1	19	1	15.6	1	0	1	0	3	1	1
1198	1	24	1	23.5	1	0	0	1	3	2	1
1199	1	19	1	16.4	1	0	0	0	3	1	1
1200	2	19	1	21.6	1	0	0	1	2	1	1
1201	1	22	1	18.0	1	0	0	0	3	1	1
1202	1	19	1	23.8	1	1	1	1	3	1	2
1203	1	25	1	34.9	3	0	1	0	3	1	1
1205	1	25	1	28.4	2	1	0	0	1	1	1
1206	1	35	2	22.3	1	1	1	0	3	2	2
1207	1	24	1	24.3	1	0	0	1	2	1	1
1208	1	19	1	27.7	2	0	1	0	3	1	1

1209	1	20	1	19.7	1	0	0	0	2	1	2
1210	2	18	1	31.8	3	0	0	0	3	1	1
1211	2	19	1	26.3	2	0	0	1	3	2	3
1212	1	20	1	25.0	1	1	1	0	2	1	2
1214	2	26	2	26.4	2	1	0	0	2	1	2
1215	1	24	1	22.7	1	1	0	0	3	1	2
1216	2	25	1	29.4	2	0	1	1	3	1	1
1217	1	19	1	20.6	1	0	0	0	3	2	2
1218	2	18	1	22.8	1	0	1	0	2	1	2
1220	2	20	1	32.6	3	1	1	0	3	1	2
1221	1	23	1	35.2	3	0	0	0	2	1	1
1222	1	24	1	23.7	1	1	0	0	1	1	1
1223	1	20	1	21.0	1	0	0	0	3	1	2
1225	2	22	1	22.1	1	1	0	0	2	1	3
1226	1	20	1	18.4	1	0	0	0	2	1	2
1227	1	19	1	21.1	1	1	1	0	3	1	1
1228	2	19	1	20.2	1	1	1	0	2	1	1
1229	1	19	1	31.2	3	1	1	0	3	1	1
1230	2	31	2	26.3	2	0	1	0	2	1	2
1231	1	19	1	21.9	1	0	0	0	2	1	1
1232	2	19	1	22.4	1	1	1	1	2	1	2
1233	2	19	1	29.2	2	1	1	0	3	1	2
1235	2	21	1	24.5	1	1	1	0	3	1	1
1236	1	19	1	22.3	1	0	0	0	3	2	1
1237	1	21	1	32.7	3	1	1	1	3	1	2
1239	1	18	1	20.2	1	0	0	0	3	2	3
1241	1	18	1	26.4	2	1	1	0	3	1	3
1242	1	18	1	21.5	1	0	0	1	2	2	1
1244	1	20	1	22.8	1	0	0	0	2	1	1
1245	1	20	1	28.5	2	0	1	0	3	1	1
1247	2	21	1	21.2	1	1	1	0	2	1	1
1248	1	18	1	19.5	1	0	0	0	2	1	1
1249	1	22	1	25.0	1	0	0	0	3	1	3
1250	1	19	1	24.8	1	1	0	0	3	1	1
1251	1	18	1	27.5	2	1	0	0	2	2	1
1252	1	20	1	18.4	1	0	0	0	3	1	1
1253	1	22	1	23.2	1	0	0	0	1	1	2
1254	1	20	1	17.7	1	1	0	0	3	1	1
1255	1	21	1	24.2	1	0	1	0	3	1	1
1256	1	18	1	22.2	1	1	1	0	3	1	3
1259	1	18	1	17.6	1	1	1	0	2	1	1
1262	1	21	1	22.3	1	1	1	0	3	2	1
1264	1	22	1	18.2	1	0	0	0	2	2	1
1265	1	23	1	18.8	1	1	0	1	2	1	3
1266	2	22	1	26.9	2	0	0	0	3	1	2
1268	2	20	1	27.2	2	0	0	0	3	1	1
1269	1	19	1	27.2	2	1	0	0	2	1	1
1270	2	19	1	27.8	2	0	0	0	3	2	2
1271	2	20	1	23.2	1	1	1	0	2	1	2
1272	2	20	1	24.2	1	0	0	0	1	1	1
1273	2	21	1	21.8	1	1	1	0	2	2	3
1275	1	19	1	28.0	2	0	0	0	3	1	1
1277	1	20	1	22.6	1	0	0	0	2	1	2
1278	2	22	1	23.4	1	1	0	0	3	1	1
1280	1	19	1	22.4	1	0	0	0	2	1	2
1281	1	20	1	22.9	1	0	0	0	3	1	1
1282	1	21	1	28.3	2	1	0	0	2	1	2
1283	1	20	1	22.5	1	1	1	0	3	1	3
1285	1	23	1	35.1	3	0	0	0	2	2	1
1287	1	19	1	18.4	1	0	0	0	2	1	1
1289	1	19	1	16.6	1	0	0	1	1	1	1
1290	1	18	1	29.4	2	0	0	1	2	1	1
1291	2	21	1	21.6	1	0	0	0	1	2	2
1292	2	25	1	32.4	3	0	0	1	3	2	3
1293	2	44	2	39.7	3	0	0	0	2	1	1
1294	2	27	2	26.5	2	1	0	0	2	1	1

1295	2	26	2	37.5	3	0	1	0	3	2	1
1296	1	20	1	24.1	1	0	1	0	2	1	1
1298	2	21	1	24.6	1	1	1	0	2	1	1
1299	1	20	1	23.8	1	0	0	0	3	1	3
1300	2	23	1	28.4	2	0	0	0	2	1	2
1302	1	29	2	28.3	2	0	0	0	3	2	1
1303	1	19	1	29.1	2	0	0	0	2	2	1
1304	1	18	1	22.3	1	0	0	1	3	1	1
1305	2	18	1	25.5	2	1	0	0	3	2	2
1308	1	18	1	29.4	2	0	0	0	2	1	2
1309	2	21	1	23.5	1	0	0	0	1	1	2
1314	1	27	2	25.0	1	0	0	0	3	2	2
1315	1	31	2	36.7	3	0	0	0	1	1	1
1317	1	24	1	25.7	2	0	0	0	2	1	2
1319	2	21	1	21.9	1	1	1	0	2	1	1
1320	2	18	1	18.4	1	0	0	0	1	1	1
1321	1	20	1	19.0	1	1	1	0	1	1	1
1322	1	18	1	19.6	1	0	0	0	2	1	1
1324	1	26	2	25.3	2	1	0	1	3	1	2
1325	1	21	1	20.1	1	0	1	0	2	1	2
1326	1	28	2	20.2	1	0	0	1	1	2	1
1327	1	24	1	23.8	1	0	1	0	2	1	1
1328	1	19	1	27.5	2	0	0	1	2	1	1
1330	2	19	1	28.4	2	0	0	0	1	1	2
1331	2	25	1	24.5	1	0	0	0	1	2	2
1333	2	22	1	19.8	1	0	0	0	2	1	1
1334	2	20	1	21.6	1	0	0	1	2	2	2
1335	1	20	1	27.0	2	0	0	0	2	1	1
1338	2	19	1	20.1	1	0	0	0	2	2	2
1339	2	23	1	18.0	1	1	1	0	2	1	2
1340	2	18	1	22.4	1	0	0	0	2	2	1
1341	1	21	1	22.2	1	0	0	0	3	2	2
1342	2	18	1	21.8	1	1	0	0	1	1	2
1345	2	19	1	24.5	1	1	0	0	3	2	3
1346	1	19	1	20.7	1	1	1	0	3	1	2
1347	2	20	1	28.0	2	0	1	1	1	1	3
1348	2	20	1	18.0	1	0	0	0	1	1	1
1349	1	22	1	21.5	1	1	1	1	2	1	1
1350	2	30	2	25.1	2	0	0	0	2	1	2
1351	2	18	1	19.6	1	1	0	1	2	2	2
1352	1	20	1	23.2	1	0	0	0	3	1	1
1354	2	18	1	25.8	2	0	0	0	1	1	1
1355	2	24	1	18.6	1	0	0	0	1	1	1
1356	1	18	1	26.2	2	1	1	0	2	2	1
1359	1	19	1	23.0	1	1	0	1	2	1	1
1361	1	23	1	21.0	1	0	0	0	3	1	3
1362	1	20	1	19.5	1	0	0	0	2	1	1
1363	1	18	1	43.1	3	0	0	0	1	1	1
1366	1	22	1	19.1	1	0	1	0	2	1	2
1367	1	20	1	21.9	1	0	1	0	2	2	1
1369	2	21	1	21.2	1	0	1	1	2	1	2
1370	1	19	1	30.3	3	0	1	1	3	1	1
1371	1	20	1	27.3	2	0	0	0	2	1	1
1372	2	20	1	25.1	2	1	0	0	2	2	3
1375	2	21	1	15.8	1	0	0	0	3	2	2
1376	1	21	1	27.3	2	0	0	0	1	1	1
1377	1	22	1	19.3	1	1	0	0	3	2	1
1378	1	19	1	19.1	1	1	0	0	1	1	1
1380	2	19	1	20.5	1	0	0	0	3	2	1
1381	2	18	1	22.2	1	0	0	1	3	2	3
1382	2	18	1	21.4	1	0	0	0	3	1	1
1383	1	27	2	20.8	1	1	1	0	2	1	1
1385	2	19	1	22.0	1	1	1	0	2	1	2
1387	1	25	1	18.2	1	0	0	1	1	2	1
1389	2	23	1	18.1	1	0	0	0	1	1	1
1390	1	20	1	18.8	1	0	0	0	3	1	1

1393	2	18	1	24.0	1	0	0	0	2	2	1
1396	1	19	1	20.4	1	0	0	1	2	1	2
1398	1	23	1	22.0	1	0	0	0	1	2	1
1399	2	27	2	25.3	2	0	0	0	3	1	1
1400	1	28	2	25.4	2	0	0	0	2	2	1
1401	1	21	1	20.0	1	0	0	0	1	2	1
1402	2	23	1	26.5	2	0	0	0	1	2	2
1404	2	24	1	22.8	1	0	1	0	2	2	1
1406	2	19	1	21.8	1	0	0	0	2	2	2
1407	1	22	1	24.0	1	0	0	0	3	1	1
1408	1	21	1	22.2	1	0	1	0	2	1	1
1409	1	18	1	24.6	1	0	0	0	3	1	1
1411	1	20	1	46.1	3	0	0	0	2	1	1
1412	1	18	1	22.5	1	0	0	0	2	1	1
1414	2	18	1	35.4	3	0	0	1	2	2	1
1416	1	20	1	25.7	2	0	0	0	3	2	1
1418	1	19	1	18.9	1	1	0	0	2	1	1
1419	1	20	1	18.9	1	1	0	0	3	1	2
1420	1	22	1	21.6	1	0	1	0	3	1	1
1421	2	21	1	30.6	3	1	1	1	2	2	3
1422	2	21	1	22.5	1	1	1	0	1	1	2
1423	1	20	1	20.4	1	1	1	0	3	2	2
1424	1	20	1	22.3	1	0	1	0	3	1	1
1427	2	23	1	22.5	1	1	1	1	2	1	3
1428	1	21	1	22.0	1	0	0	0	2	1	1
1429	1	20	1	25.3	2	0	0	0	2	2	1
1430	1	20	1	28.4	2	0	1	0	3	2	2
1431	2	22	1	31.1	3	0	0	0	2	1	1
1432	1	19	1	16.2	1	0	0	0	3	1	2
1433	1	21	1	18.1	1	1	1	1	3	1	1
1434	1	20	1	24.8	1	0	0	0	3	2	2
1436	1	19	1	22.1	1	0	0	0	2	1	3
1437	1	18	1	23.9	1	1	1	0	3	1	1
1438	1	20	1	24.8	1	0	0	0	2	1	2
1440	1	26	2	23.5	1	1	0	0	3	2	3
1441	1	20	1	33.7	3	1	1	0	2	1	2
1442	1	20	1	20.4	1	1	1	1	3	1	2
1445	2	20	1	21.8	1	0	0	0	3	2	2
1447	1	22	1	21.0	1	1	1	0	3	1	2
1448	2	18	1	19.4	1	0	0	0	3	1	2
1449	1	36	2	24.5	1	0	0	0	2	2	1
1452	2	19	1	18.2	1	1	1	0	3	1	2
1453	2	18	1	19.1	1	0	0	0	3	1	3
1456	1	21	1	30.8	3	1	1	0	2	1	2
1457	2	18	1	21.6	1	1	1	1	2	1	2
1458	1	18	1	23.5	1	1	0	0	3	1	2
1459	2	21	1	27.7	2	0	0	0	3	1	3
1460	2	20	1	24.6	1	1	1	1	2	2	1
1462	2	19	1	22.2	1	1	0	0	3	2	2
1465	1	20	1	18.2	1	1	0	0	3	1	1
1466	2	27	2	34.0	3	1	1	0	3	2	1
1467	1	23	1	23.6	1	1	0	0	2	1	1
1468	1	23	1	21.8	1	1	0	1	3	2	2
1469	1	31	2	26.8	2	1	0	0	2	1	2
1470	1	19	1	17.3	1	0	1	0	3	1	2
1471	1	23	1	24.2	1	1	0	0	3	1	2
1472	1	19	1	18.1	1	0	0	1	2	1	1
1474	1	18	1	19.1	1	0	0	0	2	1	2
1476	1	24	1	21.6	1	1	0	0	2	1	1
1477	2	19	1	21.5	1	1	1	0	2	2	1
1478	2	19	1	22.9	1	1	0	0	3	1	1
1479	1	21	1	26.4	2	1	1	0	3	1	1
1483	2	20	1	18.8	1	0	0	0	3	2	1
1484	1	23	1	20.2	1	0	0	1	3	1	3
1485	2	18	1	27.4	2	0	0	0	3	2	1
1486	1	20	1	20.2	1	1	1	0	2	1	1

1487	2	29	2	32.7	3	0	0	0	2	2	1
1488	1	20	1	18.5	1	0	0	0	3	1	2
1489	2	21	1	32.5	3	0	0	0	2	1	1
1491	1	28	2	17.3	1	0	0	0	2	2	2
1492	1	24	1	24.8	1	0	0	0	2	1	2
1493	2	20	1	24.6	1	1	0	0	3	1	2
1494	2	28	2	24.1	1	1	0	0	2	1	2
1495	2	33	2	31.1	3	1	0	0	3	1	1
1496	1	20	1	19.2	1	0	0	0	3	2	2
1497	2	23	1	22.8	1	0	0	0	2	1	3
1498	1	19	1	20.8	1	1	1	0	3	1	1
1499	1	20	1	38.1	3	1	0	0	2	1	1
1500	1	19	1	21.3	1	1	0	0	2	1	1
1502	2	27	2	19.7	1	1	1	0	2	2	1
1503	1	19	1	19.9	1	0	0	0	2	1	1
1505	1	20	1	21.0	1	1	1	0	2	1	1
1506	2	19	1	23.7	1	0	0	1	2	1	3
1507	2	22	1	30.2	3	1	0	0	1	1	1
1508	1	20	1	21.4	1	1	0	0	3	1	2
1509	2	21	1	18.8	1	0	0	0	3	1	1
1511	2	18	1	25.7	2	1	1	0	3	2	2
1512	1	19	1	23.9	1	0	0	0	2	2	1
1513	2	21	1	31.4	3	0	1	0	3	1	3
1514	2	21	1	28.4	2	0	0	1	1	2	3
1515	2	28	2	25.8	2	1	0	0	3	2	3
1516	1	21	1	21.3	1	1	1	0	3	1	1
1517	2	22	1	23.4	1	1	1	1	2	1	2
1518	1	22	1	21.1	1	1	0	0	3	1	2
1519	1	21	1	21.6	1	0	0	0	3	1	3
1520	1	18	1	27.0	2	0	0	0	2	1	1
1521	1	20	1	22.9	1	0	0	0	2	1	1
1523	2	21	1	25.3	2	0	0	0	3	1	2
1525	2	19	1	18.8	1	0	0	0	1	1	3
1526	1	19	1	20.1	1	0	0	0	3	2	2
1527	2	32	2	23.4	1	1	0	1	3	2	2
1528	1	18	1	26.6	2	1	0	0	3	1	1
1530	1	20	1	20.1	1	0	0	1	1	1	2
1532	2	18	1	16.5	1	0	0	0	2	1	1
1533	1	21	1	21.8	1	0	0	0	1	1	2
1535	1	18	1	22.9	1	0	0	0	2	1	2
1536	1	18	1	19.0	1	0	0	1	3	2	2
1537	1	22	1	23.7	1	0	0	0	3	1	2
1538	1	19	1	17.8	1	0	0	0	3	1	1
1539	2	24	1	17.7	1	0	0	1	2	1	1
1542	1	18	1	19.8	1	0	0	0	3	1	1
1544	1	22	1	18.6	1	0	0	0	3	1	1
1545	1	20	1	19.4	1	0	0	0	1	1	1
1549	1	26	2	22.3	1	0	0	0	2	2	2
1550	1	20	1	20.1	1	0	1	1	3	1	2
1552	2	19	1	31.7	3	0	1	0	2	1	1
1553	2	19	1	18.3	1	0	0	0	3	2	2
1554	1	22	1	23.4	1	0	0	1	3	1	2
1555	1	20	1	19.8	1	0	0	0	1	1	1
1556	1	24	1	21.0	1	0	0	1	2	1	2
1557	2	24	1	27.2	2	1	0	0	2	1	2
1559	2	19	1	22.1	1	1	1	1	2	1	3
1560	2	23	1	25.8	2	1	1	0	3	2	2
1561	1	22	1	22.2	1	0	0	0	3	1	1
1562	2	22	1	26.9	2	0	0	0	2	1	1
1564	1	21	1	19.7	1	1	1	0	2	1	1
1565	1	22	1	18.8	1	0	0	0	3	2	1
1566	1	31	2	25.3	2	0	0	1	2	1	2
1567	2	19	1	28.1	2	1	1	0	3	2	2
1568	1	19	1	27.3	2	0	0	0	1	1	2
1570	1	28	2	22.5	1	1	0	0	3	1	2
1571	1	23	1	20.5	1	0	0	0	2	1	2

1572	1	19	1	33.1	3	1	0	0	2	1	1
1575	1	18	1	19.5	1	0	0	0	3	2	1
1576	1	22	1	21.1	1	1	0	1	2	1	3
1578	1	18	1	17.4	1	0	0	0	3	1	2
1579	2	18	1	25.4	2	1	0	1	3	2	1
1580	2	19	1	19.0	1	1	1	0	2	2	3
1581	1	22	1	21.8	1	0	0	0	3	1	3
1582	1	20	1	31.0	3	0	0	0	2	1	1
1583	2	18	1	19.8	1	0	0	0	2	1	1
1584	1	19	1	20.6	1	0	0	1	3	1	2
1585	1	25	1	25.5	2	1	1	0	3	2	2
1586	1	30	2	26.6	2	0	0	0	2	1	2
1587	2	19	1	21.5	1	0	0	0	3	1	1
1589	1	20	1	16.7	1	1	1	0	3	2	1
1590	1	18	1	24.8	1	1	0	0	3	1	1
1591	1	28	2	24.7	1	1	1	0	3	1	1
1592	1	25	1	24.2	1	1	0	0	3	1	1
1593	1	19	1	22.1	1	0	0	0	2	1	1
1594	1	20	1	19.7	1	1	0	0	3	2	2
1595	2	21	1	49.7	3	0	0	0	1	1	1
1598	1	18	1	20.3	1	0	0	0	3	1	3
1599	1	23	1	20.8	1	0	0	0	2	1	2
1602	1	24	1	38.0	3	0	0	0	2	1	1
1603	1	20	1	26.2	2	0	0	0	2	1	2
1605	2	21	1	21.1	1	1	1	0	2	2	1
1606	1	21	1	21.7	1	1	1	0	2	1	1
1607	2	21	1	24.0	1	1	1	0	2	2	2
1608	1	20	1	25.4	2	1	0	0	3	1	2
1610	2	19	1	36.3	3	0	0	0	1	1	1
1611	1	19	1	19.1	1	0	0	0	1	1	1
1612	1	22	1	20.2	1	1	0	0	3	1	3
1613	2	24	1	18.0	1	0	1	0	2	1	1
1614	1	23	1	31.1	3	0	0	0	2	1	1
1616	2	22	1	24.0	1	1	1	0	2	1	1
1617	1	19	1	21.2	1	0	0	0	2	1	3
1618	1	22	1	20.8	1	0	0	0	3	1	1
1619	1	25	1	17.2	1	0	0	0	3	1	1
1621	1	19	1	20.6	1	0	0	0	1	1	1
1622	2	35	2	29.3	2	0	0	0	3	2	3
1624	1	20	1	21.9	1	1	1	0	3	2	1
1625	1	21	1	26.6	2	0	0	0	3	1	2
1626	2	19	1	23.2	1	0	0	0	2	1	1
1627	1	20	1	26.9	2	0	0	0	3	2	1
1628	1	20	1	21.0	1	1	0	0	3	2	2
1629	1	21	1	27.4	2	1	0	0	3	2	2
1632	1	18	1	21.2	1	1	1	0	3	1	1
1633	1	20	1	18.2	1	1	0	0	2	1	2
1634	2	30	2	22.6	1	0	0	0	1	1	1
1635	2	22	1	22.9	1	0	0	0	3	2	2
1636	2	19	1	33.2	3	1	0	0	2	1	2
1637	2	22	1	22.0	1	0	0	0	3	1	1
1638	2	24	1	21.0	1	0	0	1	1	2	2
1639	2	22	1	33.6	3	1	0	0	3	1	2
1640	2	21	1	21.4	1	0	0	1	2	2	2
1641	2	19	1	22.0	1	1	0	1	3	1	3
1643	2	21	1	18.8	1	1	1	0	2	1	1
1644	1	20	1	19.2	1	0	0	0	3	1	1
1645	1	22	1	22.3	1	0	0	0	3	1	1
1646	1	24	1	26.0	2	1	1	0	2	1	1
1649	2	24	1	24.6	1	0	1	0	3	2	3
1650	2	18	1	16.4	1	0	0	1	2	1	1
1652	1	22	1	25.8	2	0	0	0	3	1	2
1653	1	22	1	22.6	1	1	1	0	3	1	1
1654	1	20	1	24.3	1	0	0	1	3	2	1
1655	1	20	1	16.2	1	0	0	0	3	1	1
1656	1	20	1	27.5	2	0	0	1	3	1	1

1657	1	23	1	20.7	1	0	0	0	3	1	3
1658	1	18	1	33.9	3	0	0	1	3	2	2
1659	2	19	1	21.9	1	1	0	0	3	2	2
1662	2	21	1	26.9	2	1	1	1	2	1	1
1665	1	19	1	22.7	1	0	0	0	3	1	1
1667	1	20	1	19.6	1	0	0	0	3	2	1
1668	2	24	1	39.9	3	0	0	0	1	1	1
1669	2	26	2	21.7	1	1	0	0	3	2	1
1670	1	23	1	31.2	3	1	0	0	3	2	2
1671	2	23	1	28.1	2	1	1	0	1	1	1
1672	2	19	1	33.3	3	0	0	0	1	1	2
1674	1	26	2	35.1	3	1	0	1	3	1	2
1675	1	22	1	22.0	1	1	0	0	2	2	3
1676	2	18	1	25.0	1	0	0	0	1	2	2
1677	1	32	2	30.1	3	0	0	0	1	1	1
1680	1	21	1	25.7	2	0	0	0	3	1	1
1681	1	21	1	29.1	2	0	0	0	3	1	1
1682	1	23	1	22.3	1	1	1	0	2	1	2
1683	1	18	1	24.2	1	0	0	0	2	2	1
1684	1	28	2	28.7	2	0	0	0	3	1	1
1687	2	25	1	27.2	2	1	1	0	2	1	2
1688	2	20	1	27.8	2	1	1	0	3	1	1
1689	2	20	1	24.2	1	0	0	0	2	1	3
1690	1	18	1	22.7	1	0	0	0	2	1	2
1691	2	22	1	23.8	1	1	0	0	2	2	3
1693	1	19	1	19.5	1	0	0	0	2	1	1
1694	1	19	1	24.6	1	1	0	0	2	1	1
1695	1	19	1	21.3	1	1	0	0	3	1	3
1696	1	19	1	19.2	1	0	0	0	3	2	2
1697	1	22	1	20.6	1	0	0	0	3	2	1
1698	2	28	2	26.8	2	0	0	0	3	1	3
1700	1	31	2	18.8	1	0	0	1	3	1	2
1702	1	21	1	21.8	1	0	0	0	2	1	1
1703	1	21	1	24.8	1	0	0	0	3	1	2
1705	1	20	1	23.4	1	1	1	0	2	1	1
1706	1	19	1	18.4	1	0	0	1	3	1	3
1707	1	24	1	31.9	3	0	0	0	2	1	1
1708	1	22	1	18.8	1	1	0	0	3	1	1
1709	1	22	1	27.5	2	0	0	0	2	1	1
1710	2	33	2	24.9	1	1	0	0	3	2	3
1711	1	19	1	23.4	1	0	0	0	2	1	1
1712	1	18	1	28.5	2	0	0	0	1	1	1
1713	1	25	1	18.7	1	0	0	0	2	2	1
1714	1	27	2	26.7	2	0	0	0	3	2	2
1715	2	20	1	23.7	1	0	0	0	2	2	2
1717	2	23	1	29.6	2	1	1	0	2	1	2
1718	1	19	1	25.4	2	0	0	0	3	2	1
1720	1	25	1	23.9	1	1	1	0	3	2	1
1721	1	20	1	21.1	1	1	0	0	2	1	1
1722	1	19	1	19.6	1	0	0	0	1	1	1
1723	2	18	1	19.2	1	0	0	0	1	1	2
1725	1	23	1	21.3	1	0	0	0	1	1	2
1726	2	21	1	18.0	1	0	0	0	3	1	1
1729	1	18	1	23.1	1	0	0	0	3	1	1
1730	1	21	1	16.7	1	0	0	0	3	1	1
1731	1	18	1	18.9	1	0	0	0	3	2	2
1732	2	21	1	19.6	1	0	0	0	3	1	1
1733	2	20	1	19.9	1	0	0	0	3	1	1
1734	2	19	1	22.3	1	0	0	0	3	2	2
1735	1	18	1	19.1	1	0	0	0	2	2	2
1736	1	21	1	17.6	1	0	0	1	1	1	1
1737	1	29	2	23.1	1	0	0	0	2	1	1
1738	1	23	1	25.9	2	1	1	1	3	1	2
1739	2	27	2	21.6	1	0	0	0	2	2	2
1741	1	22	1	20.0	1	0	0	0	3	1	2
1742	2	21	1	20.4	1	0	0	0	2	2	3

1743	1	24	1	22.5	1	0	0	0	3	1	1
1744	2	25	1	23.0	1	1	1	1	3	1	3
1746	2	30	2	26.5	2	0	0	0	2	1	2
1748	2	25	1	23.8	1	1	1	0	2	1	1
1751	1	22	1	22.1	1	0	0	0	2	1	1
1752	2	21	1	20.8	1	0	0	0	1	1	1
1756	2	20	1	18.9	1	0	0	0	1	1	1
1757	1	28	2	24.8	1	0	0	0	2	1	1
1758	1	19	1	32.0	3	0	1	0	2	1	2
1759	1	23	1	18.8	1	0	1	0	3	1	1
1760	1	22	1	22.5	1	0	0	0	3	1	1
1761	1	20	1	23.0	1	0	0	0	2	2	2
1764	2	20	1	23.2	1	1	0	0	1	2	3
1765	1	18	1	22.4	1	1	1	1	1	1	2
1768	1	18	1	26.0	2	0	0	1	2	2	2
1770	1	19	1	24.5	1	0	1	1	2	1	2
1771	1	21	1	30.8	3	1	0	1	3	2	2
1773	1	22	1	22.7	1	0	0	0	3	1	1
1774	1	27	2	29.4	2	0	0	0	3	1	1
1775	1	24	1	26.3	2	1	0	1	2	1	1
1777	1	21	1	19.5	1	0	0	0	3	1	1
1779	2	22	1	19.6	1	0	0	0	3	1	2
1781	2	19	1	20.1	1	0	0	0	3	2	1
1782	1	19	1	25.8	2	1	0	0	3	1	1
1784	1	18	1	19.0	1	0	0	0	3	1	1
1785	2	19	1	23.5	1	0	0	0	2	1	1
1786	1	19	1	18.6	1	0	0	0	3	1	2
1787	1	19	1	20.1	1	0	1	0	2	1	1
1788	2	18	1	18.3	1	0	1	0	3	1	1
1789	1	19	1	30.1	3	1	1	0	1	1	1
1790	1	19	1	21.6	1	0	0	0	3	1	2
1791	2	19	1	20.8	1	0	0	0	2	2	2
1793	1	20	1	23.8	1	0	0	0	3	1	2
1795	1	19	1	24.4	1	0	0	0	3	1	2
1796	1	20	1	19.0	1	0	0	0	2	2	1
1798	1	24	1	25.4	2	0	0	0	3	1	1
1800	1	22	1	19.1	1	0	0	0	2	2	1
1801	1	19	1	19.9	1	0	0	0	2	2	2
1802	1	18	1	26.1	2	0	0	0	2	1	3
1804	1	18	1	21.9	1	0	0	0	1	1	2
1805	1	18	1	22.2	1	0	0	0	3	1	1
1806	2	19	1	25.3	2	0	1	1	3	1	3
1807	2	20	1	24.9	1	0	1	0	2	1	3
1808	1	20	1	23.0	1	1	1	0	3	1	2
1809	2	23	1	25.5	2	1	1	0	2	1	1
1811	1	20	1	21.2	1	0	0	0	3	2	1
1812	1	22	1	27.0	2	0	0	0	3	1	1
1813	1	21	1	16.9	1	0	0	0	2	1	1
1815	1	19	1	23.3	1	0	0	1	2	2	2
1816	1	25	1	37.6	3	0	0	1	2	1	2
1817	1	23	1	24.2	1	0	0	0	3	1	1
1818	1	21	1	20.2	1	0	0	0	2	1	1
1819	1	19	1	22.9	1	0	0	0	3	1	1
1820	1	21	1	33.3	3	0	0	0	3	1	1
1821	2	20	1	21.8	1	0	1	0	2	2	1
1822	2	21	1	32.8	3	0	0	0	2	1	2
1823	2	21	1	29.7	2	1	0	0	1	1	1
1825	2	23	1	26.4	2	1	1	0	3	1	1
1827	1	20	1	18.9	1	0	0	0	2	1	1
1828	2	24	1	18.3	1	1	1	0	2	1	1
1829	2	22	1	19.8	1	1	0	0	2	2	2
1830	1	18	1	17.9	1	0	0	0	3	2	1
1831	2	21	1	24.8	1	0	1	0	3	2	2
1832	1	22	1	17.4	1	1	1	0	2	1	2
1833	1	21	1	22.3	1	0	0	0	3	1	3
1834	1	19	1	20.5	1	0	0	0	3	1	1

1835	2	19	1	35.5	3	0	0	0	2	2	1
1837	1	18	1	18.8	1	0	0	0	3	1	2
1838	2	20	1	22.5	1	1	0	1	1	1	3
1841	1	18	1	20.8	1	0	0	0	3	1	1
1843	2	21	1	21.2	1	0	1	0	3	2	2
1844	1	18	1	23.9	1	1	1	0	2	1	2
1845	1	21	1	22.3	1	1	0	0	3	1	1
1846	2	18	1	23.0	1	0	0	1	2	1	2
1847	2	23	1	25.1	2	0	0	0	2	1	2
1848	2	18	1	22.7	1	1	0	0	2	1	2
1850	1	20	1	30.0	3	1	1	0	3	1	2
1851	1	19	1	21.0	1	1	0	0	1	1	1
1852	2	22	1	19.6	1	0	0	0	3	2	2
1854	1	20	1	16.6	1	0	0	1	1	1	1
1855	1	27	2	28.9	2	0	0	0	1	1	1
1856	1	20	1	22.2	1	0	0	0	3	1	1
1857	2	22	1	20.5	1	1	1	0	2	1	3
1858	2	19	1	19.6	1	0	0	0	2	1	2
1860	1	19	1	18.0	1	0	0	0	3	2	2
1861	1	25	1	25.5	2	1	0	0	2	1	1
1862	2	21	1	18.1	1	1	0	0	2	1	1
1863	2	22	1	33.5	3	0	0	0	2	2	1
1864	2	21	1	24.2	1	1	0	0	3	1	2
1865	1	19	1	22.8	1	0	0	0	3	1	1
1867	1	18	1	22.7	1	0	0	1	1	1	1
1868	2	19	1	18.7	1	1	0	0	2	1	1
1869	1	26	2	22.9	1	1	1	0	3	1	1
1870	1	18	1	21.4	1	0	0	0	3	1	1
1872	2	20	1	35.5	3	1	0	0	2	1	1
1876	2	21	1	23.4	1	0	0	0	1	1	1
1877	2	20	1	21.4	1	1	1	0	3	2	2
1878	1	18	1	28.4	2	0	0	0	1	1	1
1879	2	20	1	22.2	1	0	0	0	3	1	2
1880	2	20	1	27.2	2	0	0	0	2	2	1
1881	1	19	1	18.9	1	0	0	1	2	1	2
1883	1	24	1	22.5	1	1	0	0	3	1	2
1884	2	21	1	21.0	1	1	1	0	2	2	2
1885	2	26	2	27.7	2	0	0	0	3	2	2
1886	1	23	1	18.3	1	1	0	0	3	2	1
1890	2	24	1	19.8	1	0	0	0	3	1	1
1891	1	21	1	19.8	1	1	0	0	2	1	2
1893	1	22	1	24.8	1	0	1	0	2	1	1
1894	2	19	1	42.2	3	0	0	1	3	1	1
1896	2	20	1	21.6	1	0	0	0	2	1	1
1899	1	21	1	20.8	1	0	0	0	2	1	3
1900	2	22	1	23.4	1	0	0	0	3	1	1
1901	2	19	1	23.1	1	1	1	0	1	1	3
1902	1	24	1	18.2	1	0	0	0	2	1	2
1903	1	24	1	19.8	1	0	0	0	2	1	1
1904	1	26	2	22.6	1	1	0	0	2	1	1
1905	1	24	1	28.9	2	0	1	0	2	1	2
1908	1	22	1	25.4	2	1	1	1	3	1	2
1909	1	19	1	24.3	1	1	1	0	3	1	2
1910	1	22	1	24.2	1	0	0	0	3	1	2
1912	1	24	1	19.0	1	0	0	0	3	2	2
1913	2	21	1	23.5	1	1	1	0	3	1	2
1914	2	25	1	20.5	1	1	0	0	3	1	2
1915	1	24	1	22.1	1	0	0	1	3	2	1
1916	1	21	1	25.6	2	0	0	0	2	1	1
1917	1	22	1	25.0	1	0	0	1	3	1	2
1919	2	21	1	29.4	2	0	0	0	3	1	1
1920	1	22	1	26.0	2	1	0	1	3	1	3
1921	1	22	1	38.4	3	1	1	0	2	1	1
1922	1	22	1	22.0	1	1	0	1	3	1	3
1924	1	18	1	28.0	2	0	0	0	3	2	3
1925	2	22	1	31.0	3	1	0	0	2	1	2

1926	1	21	1	20.2	1	1	0	0	3	2	2
1929	1	19	1	26.1	2	0	0	0	3	2	1
1930	2	23	1	29.1	2	0	0	0	2	2	1
1931	1	19	1	21.1	1	0	0	0	2	1	2
1932	1	21	1	23.1	1	0	0	0	2	1	1
1933	2	21	1	21.2	1	0	0	0	3	1	1
1934	1	22	1	26.4	2	0	0	0	3	1	2
1936	1	24	1	21.5	1	1	0	0	3	1	2
1937	2	39	2	36.0	3	0	1	0	1	1	1
1938	2	32	2	24.5	1	0	0	1	1	1	2
1939	1	21	1	21.7	1	1	0	0	3	1	1
1940	2	19	1	21.9	1	0	0	0	2	2	3
1941	1	20	1	24.0	1	0	0	0	2	1	1
1942	1	21	1	22.9	1	0	0	0	2	1	1
1943	1	19	1	19.7	1	0	0	0	2	2	1
1944	1	19	1	18.8	1	0	0	0	2	1	1
1945	1	23	1	25.0	1	0	0	1	2	1	2
1946	1	18	1	17.6	1	0	0	0	2	1	1
1947	2	28	2	31.1	3	0	0	0	2	2	2
1950	1	22	1	24.1	1	0	0	1	2	2	3
1951	1	21	1	22.9	1	1	1	0	1	1	1
1952	2	19	1	19.1	1	1	1	0	2	1	1
1953	1	22	1	18.0	1	0	0	0	3	1	2
1954	1	18	1	20.7	1	1	0	1	3	2	3
1955	2	23	1	31.6	3	0	0	0	3	1	1
1956	1	24	1	25.4	2	0	1	0	2	1	2
1957	2	19	1	29.1	2	0	0	0	2	1	2
1958	2	20	1	24.2	1	1	0	0	2	1	2
1959	2	23	1	22.5	1	0	0	1	3	2	3
1961	1	33	2	22.6	1	0	0	0	1	1	1
1962	1	20	1	25.0	1	0	0	0	2	2	3
1963	1	20	1	34.6	3	0	0	0	1	1	2
1964	2	21	1	17.7	1	0	0	0	2	2	2
1965	1	20	1	19.6	1	0	0	0	3	1	2
1966	2	19	1	31.9	3	1	1	0	1	1	1
1968	2	21	1	22.5	1	0	0	0	2	1	3
1969	1	21	1	21.6	1	0	0	0	3	2	2
1970	2	21	1	23.2	1	0	0	0	3	2	3
1971	2	18	1	23.7	1	0	0	0	2	1	1
1972	1	19	1	22.2	1	1	1	1	3	1	1
1973	1	20	1	19.4	1	0	0	0	3	2	1
1974	1	28	2	16.6	1	0	0	1	3	2	3
1976	2	20	1	26.2	2	1	1	0	2	1	1
1977	2	19	1	20.6	1	1	0	0	2	1	2
1978	2	22	1	20.0	1	0	0	0	1	1	3
1979	1	18	1	22.6	1	0	0	0	2	1	1
1980	1	18	1	19.4	1	0	0	1	3	1	1
1981	1	23	1	27.1	2	0	0	0	3	1	1
1982	1	23	1	17.6	1	0	0	0	2	2	1
1984	2	20	1	25.3	2	0	0	0	2	2	2
1985	1	20	1	29.7	2	0	0	0	3	2	3
1986	2	21	1	20.2	1	0	0	0	2	2	2
1987	2	24	1	25.9	2	0	1	1	3	2	3
1988	2	22	1	22.5	1	0	0	0	1	2	3
1989	2	19	1	20.8	1	1	1	0	3	1	2
1990	2	18	1	25.4	2	0	0	0	1	1	3
1993	2	18	1	24.1	1	0	0	1	3	2	3
1994	1	19	1	22.3	1	0	0	0	2	1	1
1995	1	19	1	23.3	1	0	0	0	3	1	2
1996	1	19	1	30.9	3	0	0	1	3	1	3
1997	1	19	1	26.1	2	1	0	0	3	1	2
1998	1	19	1	22.7	1	1	0	0	3	1	2
2000	1	19	1	24.0	1	0	0	0	1	1	1
2001	1	19	1	20.8	1	1	0	0	3	1	1
2003	1	21	1	19.9	1	0	1	0	3	1	1
2004	1	19	1	27.9	2	0	0	0	3	1	1

2005	1	19	1	22.9	1	0	0	0	3	1	2
2006	1	20	1	21.5	1	1	1	0	3	1	2
2009	1	23	1	23.7	1	0	0	0	2	2	3
2010	1	21	1	16.5	1	0	0	0	2	2	1
2012	1	22	1	23.2	1	0	1	0	2	1	1
2014	1	21	1	19.5	1	0	0	0	3	2	1
2015	1	22	1	22.2	1	0	0	0	2	2	2
2016	1	20	1	18.4	1	0	0	1	3	2	1
2017	1	20	1	24.2	1	1	0	0	3	1	2
2019	1	18	1	19.5	1	0	0	0	1	1	1
2020	1	22	1	26.6	2	0	0	0	3	1	2
2022	1	19	1	26.8	2	0	0	0	2	1	1
2024	1	20	1	25.1	2	0	0	1	3	2	3
2026	1	20	1	19.5	1	0	0	0	3	1	2
2027	1	21	1	20.5	1	0	1	0	3	1	1
2028	1	20	1	21.0	1	0	1	0	3	1	1
2029	2	20	1	26.5	2	0	0	0	2	1	3
2030	2	23	1	22.9	1	0	0	0	2	1	1
2031	1	19	1	18.7	1	0	0	0	3	1	2
2032	1	21	1	26.6	2	1	0	0	1	1	2
2033	1	18	1	21.5	1	0	1	0	2	1	1
2034	2	21	1	24.4	1	0	0	1	2	1	2
2035	1	22	1	20.8	1	1	0	0	3	1	1
2037	2	24	1	29.6	2	1	0	0	3	1	1
2038	1	20	1	19.4	1	0	0	0	1	1	2
2039	1	25	1	19.5	1	0	0	1	2	1	2
2040	1	21	1	23.0	1	0	0	0	2	1	1
2041	1	19	1	19.8	1	0	0	0	3	2	1
2042	2	20	1	21.9	1	1	1	1	2	2	2
2043	1	20	1	18.3	1	0	0	0	3	2	1
2044	1	19	1	33.9	3	0	0	0	3	1	1
2045	1	18	1	22.0	1	0	0	0	3	1	1
2046	1	19	1	21.4	1	1	0	0	2	1	1
2047	2	22	1	19.6	1	1	0	1	2	1	2
2049	1	22	1	20.2	1	0	0	0	3	2	1
2050	1	19	1	20.3	1	0	0	0	3	2	2
2051	1	34	2	25.0	1	1	0	0	3	1	1
2052	1	18	1	18.4	1	0	0	0	3	1	1
2053	2	18	1	25.4	2	1	1	0	2	1	1
2056	2	48	2	29.4	2	1	0	0	2	2	2
2057	2	37	2	43.1	3	0	1	0	1	1	1
2058	1	26	2	33.7	3	0	0	0	3	1	2
2060	1	19	1	25.0	1	0	0	0	3	2	1
2061	1	28	2	27.5	2	1	0	0	2	1	3
2062	1	22	1	20.1	1	0	1	1	3	1	1
2065	1	21	1	22.1	1	0	0	0	3	1	1
2067	1	22	1	20.6	1	1	0	0	2	2	2
2070	1	23	1	14.5	1	0	0	0	3	1	1
2071	1	22	1	29.8	2	1	1	0	3	1	1
2073	1	20	1	19.1	1	1	0	0	3	2	2
2075	1	21	1	19.6	1	1	0	0	3	1	2
2076	2	21	1	23.1	1	0	0	0	2	1	1
2079	2	20	1	25.9	2	0	0	1	2	1	2
2080	2	20	1	23.5	1	0	0	0	3	2	2
2082	1	20	1	33.2	3	0	1	0	1	1	2
2083	1	19	1	19.3	1	0	0	0	3	2	1
2085	1	19	1	30.0	3	1	0	0	2	1	1
2087	2	20	1	42.4	3	0	1	0	2	1	1
2090	1	19	1	20.7	1	0	0	0	3	1	1
2091	1	20	1	20.8	1	0	1	1	3	1	1
2093	1	20	1	20.1	1	0	0	0	1	1	1
2094	1	24	1	26.6	2	1	1	0	2	2	2
2095	1	25	1	32.9	3	0	0	1	3	2	1
2096	1	23	1	26.1	2	0	0	0	1	1	2
2097	1	22	1	25.1	2	0	0	0	2	1	1
2098	2	22	1	25.3	2	1	1	1	1	1	2

2099	1	20	1	23.9	1	1	1	1	3	1	1
2100	1	18	1	21.4	1	0	0	0	2	1	1
2102	2	25	1	22.9	1	0	0	0	3	2	2
2103	1	22	1	22.6	1	0	0	1	3	1	2
2105	1	18	1	18.9	1	0	0	0	3	1	2
2106	1	18	1	18.0	1	0	0	0	2	1	1
2108	1	19	1	20.3	1	0	0	0	2	2	2
2109	2	29	2	30.2	3	0	0	0	3	2	1
2110	2	26	2	25.3	2	1	1	0	3	1	1
2112	1	18	1	22.0	1	0	0	0	3	1	2
2113	1	19	1	27.4	2	0	0	0	2	1	1
2114	1	19	1	21.2	1	1	1	0	3	2	2
2116	2	18	1	16.7	1	0	0	0	2	1	1
2117	1	21	1	15.8	1	0	0	0	3	2	1
2118	2	20	1	23.2	1	0	0	0	3	1	1
2120	1	18	1	19.7	1	1	1	0	2	1	2
2121	1	18	1	20.4	1	1	1	1	3	1	2
2122	1	21	1	25.0	1	0	0	0	2	1	1
2123	1	20	1	21.2	1	0	0	1	3	2	3
2124	1	19	1	32.4	3	0	0	0	3	2	2
2125	1	21	1	23.1	1	0	0	0	2	1	2
2126	1	20	1	19.5	1	0	0	1	3	2	2
2127	2	21	1	20.4	1	1	1	0	3	1	2
2128	1	19	1	21.5	1	0	0	1	3	1	2
2129	1	19	1	22.5	1	0	0	0	1	1	1
2132	1	21	1	24.5	1	0	0	0	3	1	1
2133	2	20	1	25.4	2	0	0	0	2	1	3
2136	1	32	2	31.4	3	0	1	0	2	1	1
2138	1	21	1	24.2	1	1	1	1	3	2	2
2139	2	21	1	25.3	2	0	1	1	3	2	3
2140	1	22	1	26.8	2	1	1	1	2	1	2
2141	2	21	1	21.0	1	0	0	0	2	2	2
2142	1	22	1	28.4	2	1	1	0	2	1	1
2143	2	22	1	24.6	1	0	1	0	2	1	1
2144	1	21	1	19.0	1	0	0	0	3	1	1
2145	1	20	1	27.3	2	1	0	0	3	1	2
2146	1	21	1	20.5	1	1	1	0	1	1	3
2147	1	25	1	18.7	1	0	0	0	2	2	3
2148	1	18	1	18.3	1	0	1	0	2	1	2
2149	1	22	1	34.2	3	1	0	0	2	1	1
2150	1	20	1	19.5	1	1	0	0	2	1	2
2151	1	22	1	19.5	1	0	0	0	2	1	3
2153	1	19	1	19.1	1	0	0	0	3	2	1
2154	1	21	1	25.9	2	0	0	0	3	1	2
2157	2	22	1	17.0	1	0	0	1	3	2	1
2158	1	31	2	20.8	1	0	0	0	1	1	1
2159	2	18	1	22.0	1	0	0	0	3	2	3
2160	1	25	1	29.2	2	1	1	0	3	1	3
2162	1	21	1	18.8	1	0	0	1	3	1	1
2163	1	20	1	14.5	1	1	0	0	2	1	2
2164	1	19	1	24.7	1	0	0	1	2	1	2
2165	1	18	1	17.5	1	0	0	0	1	1	1
2166	1	21	1	27.2	2	0	0	0	3	1	2
2169	1	20	1	21.9	1	0	0	0	3	1	1
2170	1	21	1	18.6	1	0	0	0	3	1	2
2171	2	20	1	28.1	2	1	1	0	2	1	2
2172	2	27	2	21.8	1	0	0	0	3	1	2
2173	2	19	1	25.5	2	0	0	0	1	1	1
2174	1	19	1	18.2	1	1	1	0	3	1	2
2175	1	19	1	24.1	1	0	0	0	3	1	1
2176	1	20	1	29.9	2	0	0	0	3	1	1
2177	2	21	1	22.5	1	0	0	0	2	1	1
2178	1	23	1	27.3	2	0	0	0	3	1	3
2179	1	30	2	18.8	1	0	0	0	2	1	1
2180	1	21	1	20.8	1	1	0	0	3	1	2
2181	1	30	2	22.1	1	0	0	0	1	1	1

2185	1	41	2	30.0	3	0	0	0	1	1	1
2186	2	24	1	24.2	1	0	1	0	3	1	2
2188	1	31	2	25.6	2	0	0	0	1	1	1
2190	1	20	1	17.5	1	1	0	0	2	1	1
2191	1	23	1	27.0	2	0	0	1	3	2	1
2192	1	20	1	20.0	1	0	0	0	3	2	2
2193	1	18	1	28.5	2	0	0	0	3	2	1
2194	2	22	1	24.4	1	0	0	0	3	1	2
2196	1	19	1	20.8	1	0	1	0	3	1	1
2197	1	25	1	20.8	1	0	0	0	2	1	2
2198	1	21	1	20.8	1	0	0	0	2	1	1
2199	1	24	1	34.5	3	0	0	0	1	1	2
2200	1	34	2	17.0	1	0	0	0	1	1	1
2201	2	24	1	22.5	1	0	0	0	3	1	2
2202	1	22	1	17.2	1	1	1	0	3	1	2
2205	1	20	1	17.0	1	0	0	0	3	1	1
2206	2	20	1	29.7	2	0	1	1	2	2	2
2207	2	20	1	18.7	1	0	0	0	2	1	2
2208	1	21	1	31.7	3	0	0	0	2	1	1
2210	1	23	1	23.0	1	0	0	0	3	1	2
2212	1	22	1	16.5	1	0	0	0	2	2	1
2214	1	20	1	23.0	1	0	0	0	1	1	3
2216	1	20	1	26.1	2	0	0	1	2	2	1
2217	2	21	1	27.1	2	0	1	0	3	1	1
2219	2	21	1	17.8	1	0	0	0	2	1	1
2220	1	24	1	19.5	1	0	0	0	2	2	1
2221	2	19	1	15.8	1	0	0	0	1	1	1
2222	1	22	1	19.5	1	0	0	0	2	1	1
2223	2	22	1	31.1	3	0	0	0	2	1	1
2224	1	23	1	21.2	1	1	0	0	3	1	1
2225	1	21	1	17.6	1	0	0	0	3	1	1
2226	1	21	1	22.5	1	0	0	0	3	2	1
2227	1	24	1	20.2	1	0	0	0	3	1	1
2229	1	19	1	29.4	2	0	0	0	3	1	2
2230	2	19	1	15.7	1	0	0	1	2	2	2
2231	1	18	1	17.3	1	1	0	0	3	1	1
2232	1	22	1	20.3	1	1	0	0	3	1	1
2234	2	20	1	19.4	1	0	0	0	3	1	1
2237	2	18	1	26.6	2	0	0	0	1	1	1
2238	1	22	1	21.2	1	0	0	0	3	1	1
2240	2	18	1	22.5	1	1	0	0	2	1	1
2241	1	18	1	21.5	1	0	0	1	3	1	3
2242	1	51	2	20.4	1	0	0	0	3	2	2
2243	2	20	1	18.1	1	0	1	0	2	1	3
2245	2	21	1	22.0	1	1	0	1	2	2	2
2247	1	18	1	19.9	1	0	0	0	1	1	1
2248	1	19	1	25.7	2	0	0	0	3	2	2
2249	1	30	2	20.8	1	0	0	0	2	1	1
2250	1	18	1	23.9	1	0	0	0	3	1	1
2252	2	23	1	26.9	2	0	0	1	3	1	3
2254	2	23	1	32.3	3	0	0	0	3	1	1
2255	1	20	1	24.0	1	1	1	0	2	1	1
2256	1	20	1	19.9	1	0	0	0	3	1	1
2257	1	18	1	19.4	1	0	0	0	1	1	1
2258	2	37	2	24.6	1	1	1	1	3	1	1
2259	1	21	1	23.7	1	1	0	0	2	1	2
2260	1	29	2	18.4	1	0	0	0	1	2	1
2261	1	19	1	17.4	1	0	0	0	2	1	2
2262	1	20	1	26.2	2	1	0	0	3	1	2
2263	1	22	1	24.3	1	1	1	0	3	1	2
2265	1	21	1	17.1	1	1	0	0	3	1	2
2267	1	19	1	26.7	2	1	1	0	3	1	1
2268	2	23	1	18.8	1	0	1	0	2	1	1
2270	1	22	1	22.7	1	0	0	0	2	1	2
2271	1	19	1	21.9	1	1	0	0	3	1	2
2272	1	22	1	19.4	1	0	0	1	2	1	1

2273	1	20	1	26.0	2	1	0	0	3	2	1
2274	1	18	1	26.5	2	0	0	0	3	2	2
2275	1	38	2	21.8	1	0	0	1	3	1	1
2276	1	33	2	20.8	1	0	0	0	1	1	1
2277	1	20	1	15.8	1	1	1	0	2	1	1
2279	1	22	1	17.6	1	0	0	1	3	1	1
2283	1	20	1	23.7	1	1	1	1	3	1	2
2284	2	28	2	28.4	2	0	1	0	3	1	2
2285	1	19	1	37.3	3	0	0	0	2	1	1
2286	1	23	1	30.1	3	1	1	0	3	1	1
2287	1	18	1	18.1	1	0	0	0	2	1	1
2288	1	35	2	23.5	1	0	0	0	2	2	1
2289	1	19	1	16.6	1	1	0	0	3	1	3
2290	1	21	1	21.2	1	0	0	0	1	1	2
2291	1	21	1	20.2	1	0	0	0	2	1	1
2293	1	18	1	22.5	1	0	0	0	2	1	2
2295	1	31	2	26.0	2	0	0	0	1	1	1
2296	1	24	1	20.3	1	1	0	0	3	1	1
2298	1	21	1	21.7	1	0	0	0	3	1	1
2300	1	18	1	23.1	1	0	0	1	3	1	2
2301	1	18	1	33.7	3	1	1	0	1	1	1
2302	1	23	1	25.6	2	1	1	1	3	1	3
2303	1	39	2	19.4	1	0	0	1	2	2	2
2304	1	19	1	19.1	1	0	0	0	1	1	1
2305	1	21	1	20.9	1	1	0	0	3	1	2
2306	1	18	1	23.0	1	0	1	0	2	1	1
2307	1	20	1	16.6	1	1	0	0	2	1	1
2308	1	20	1	18.0	1	0	0	1	3	2	1
2309	1	21	1	26.3	2	1	0	0	3	1	1
2310	1	22	1	18.4	1	0	1	0	3	1	2
2311	1	23	1	21.7	1	0	1	0	2	2	1
2312	2	21	1	27.3	2	1	1	0	2	2	1
2314	1	20	1	18.4	1	1	1	0	2	1	1
2315	2	18	1	27.8	2	1	1	0	2	1	2
2316	1	18	1	22.9	1	0	1	0	1	1	2
2317	1	21	1	27.3	2	0	0	0	3	1	2
2319	1	20	1	19.8	1	1	1	0	3	1	1
2320	2	19	1	25.9	2	1	1	1	3	1	3
2321	1	20	1	21.5	1	0	0	0	2	1	2
2322	2	24	1	22.6	1	1	1	1	2	2	3
2323	2	23	1	27.1	2	1	1	0	3	2	1
2325	1	22	1	24.2	1	0	1	0	3	1	3
2326	2	21	1	26.2	2	0	0	0	2	1	3
2327	1	25	1	18.9	1	0	0	0	2	1	1
2328	2	20	1	22.2	1	0	0	0	2	2	3
2329	2	22	1	34.3	3	0	0	1	3	2	2
2330	1	20	1	21.5	1	0	0	0	2	1	2
2331	1	20	1	22.7	1	1	1	0	2	1	1
2332	1	21	1	19.2	1	0	0	0	1	1	2
2334	1	21	1	22.4	1	0	0	0	2	1	1
2335	1	21	1	20.5	1	0	0	0	2	1	1
2337	1	23	1	28.4	2	0	0	0	3	1	1
2338	2	20	1	20.6	1	0	0	0	2	2	1
2339	2	20	1	31.0	3	1	1	0	3	2	3
2340	1	21	1	18.6	1	0	0	0	1	1	1
2342	1	27	2	17.7	1	0	0	0	1	1	2
2343	2	19	1	23.8	1	1	0	0	2	2	3
2344	1	20	1	27.7	2	0	0	0	1	1	1
2346	2	24	1	29.4	2	0	0	0	2	2	1
2347	1	20	1	19.1	1	1	0	0	3	1	2
2348	1	22	1	16.8	1	1	1	0	2	1	1
2349	1	20	1	22.0	1	0	0	0	1	1	1
2350	1	20	1	24.9	1	0	0	0	2	1	1
2351	1	32	2	24.2	1	0	0	0	3	1	1
2352	1	20	1	22.9	1	0	0	0	2	1	1
2354	1	19	1	22.8	1	1	1	0	3	1	1

2355	1	19	1	24.5	1	1	0	0	2	1	1
2357	1	23	1	20.9	1	1	1	0	3	1	2
2358	1	35	2	28.4	2	0	0	0	3	1	1
2359	1	24	1	27.7	2	0	0	0	1	1	2
2361	1	21	1	21.1	1	0	0	0	3	1	1
2362	1	18	1	20.9	1	0	0	0	3	1	1
2363	2	21	1	22.1	1	1	1	0	3	1	2
2365	2	20	1	24.8	1	0	1	1	2	2	3
2366	2	25	1	28.4	2	0	0	0	3	1	2
2367	2	19	1	17.3	1	1	1	0	2	1	1
2369	2	20	1	21.4	1	1	1	0	3	1	1
2370	2	22	1	22.6	1	0	0	0	3	1	1
2371	1	23	1	21.7	1	1	0	0	3	1	2
2373	2	22	1	22.1	1	1	1	0	2	1	3
2374	1	26	2	26.4	2	1	1	0	2	1	2
2376	1	19	1	20.0	1	0	0	0	3	1	1
2377	2	19	1	27.5	2	1	0	0	2	2	2
2378	2	20	1	23.3	1	0	0	0	2	1	1
2379	2	18	1	26.4	2	0	0	1	1	1	3
2381	2	19	1	25.7	2	0	0	0	2	1	2
2383	1	18	1	17.9	1	0	1	0	1	1	1
2384	2	20	1	21.9	1	0	0	0	3	2	2
2385	1	18	1	20.8	1	1	1	0	2	1	2
2386	2	20	1	19.0	1	0	0	0	3	1	2
2388	1	21	1	17.4	1	0	0	0	3	1	1
2391	1	18	1	19.1	1	0	0	0	2	1	1
2393	1	18	1	21.3	1	0	0	1	3	1	1
2394	1	22	1	21.5	1	0	0	1	2	2	2
2395	1	19	1	34.6	3	1	1	0	3	1	1
2397	2	22	1	24.5	1	0	0	0	3	1	1
2398	1	18	1	24.8	1	0	0	0	1	1	1
2399	1	22	1	22.4	1	1	0	0	1	1	3
2400	1	24	1	26.0	2	1	0	0	3	2	1
2401	1	18	1	20.9	1	0	0	0	2	1	1
2403	2	19	1	27.7	2	0	0	0	2	1	1
2406	1	21	1	30.5	3	1	0	0	3	1	2
2407	2	19	1	19.9	1	1	1	0	2	2	2
2408	2	22	1	20.7	1	0	0	0	2	2	3
2409	2	23	1	25.0	2	0	0	0	3	2	2
2410	1	26	2	17.2	1	0	0	1	3	2	1
2411	2	18	1	24.7	1	1	0	1	1	1	1
2414	2	20	1	23.4	1	0	0	0	1	2	2
2415	1	22	1	23.1	1	0	0	1	3	1	3
2416	1	19	1	25.0	1	1	0	1	3	1	2
2417	1	18	1	21.5	1	0	1	0	2	1	1
2418	1	20	1	20.3	1	1	0	0	3	2	1
2420	1	21	1	33.8	3	0	0	0	3	1	1
2421	2	21	1	19.3	1	0	0	0	3	2	1
2422	1	21	1	18.6	1	1	1	0	3	2	2
2423	1	22	1	29.6	2	0	0	0	2	1	1
2425	1	21	1	22.5	1	0	0	0	2	1	2
2426	1	23	1	20.7	1	0	0	1	2	1	3
2427	1	21	1	19.8	1	1	0	1	2	1	1
2429	1	18	1	22.9	1	0	0	0	1	1	2
2431	2	24	1	23.9	1	1	0	0	3	2	2
2433	1	21	1	21.1	1	0	0	1	3	2	2
2434	1	22	1	18.6	1	1	1	1	3	2	2
2435	1	18	1	19.7	1	0	0	1	3	2	2
2436	1	25	1	18.4	1	0	0	0	3	1	2
2437	1	21	1	19.1	1	1	0	1	3	1	2
2438	1	21	1	29.3	2	0	0	1	3	1	1
2439	1	21	1	24.1	1	0	0	1	2	2	2
2441	1	18	1	20.1	1	1	1	1	3	1	2
2442	2	18	1	23.6	1	1	1	0	3	1	2
2443	2	29	2	29.1	2	1	0	0	3	1	1
2445	1	19	1	21.3	1	0	1	0	2	1	1

2447	2	25	1	25.6	2	1	1	0	2	1	1
2449	1	20	1	27.5	2	1	1	0	2	1	1
2450	1	24	1	25.5	2	0	0	0	2	1	1
2452	1	19	1	20.0	1	0	0	0	3	1	1
2453	1	21	1	24.1	1	1	0	0	2	1	2
2454	1	21	1	25.4	2	0	0	0	1	1	1
2456	1	29	2	24.1	1	0	0	1	1	1	2
2458	1	19	1	26.4	2	0	1	0	3	2	2
2459	1	23	1	19.0	1	0	0	0	2	1	1
2460	1	19	1	22.2	1	1	1	0	3	1	2
2461	1	24	1	23.5	1	0	0	0	3	2	3
2463	1	22	1	21.9	1	1	0	0	2	1	2
2464	1	20	1	19.5	1	1	0	0	3	1	1
2465	1	20	1	20.7	1	0	0	0	2	1	2
2466	2	22	1	22.9	1	0	0	0	3	1	1
2467	1	22	1	18.7	1	1	1	0	2	1	1
2468	1	21	1	28.0	2	0	0	0	2	1	1
2469	2	22	1	18.5	1	0	1	0	2	1	1
2470	1	22	1	31.5	3	0	0	0	2	1	2
2471	2	22	1	26.0	2	0	0	0	3	1	1
2472	1	26	2	23.4	1	0	0	0	2	1	1
2473	1	24	1	23.7	1	1	0	0	2	1	1
2474	1	23	1	20.3	1	1	1	0	2	1	2
2475	1	22	1	24.9	1	0	0	0	3	1	2
2476	2	26	2	25.2	2	1	1	0	2	1	2
2477	1	18	1	22.1	1	0	0	0	1	1	1
2480	1	24	1	19.8	1	0	0	0	3	1	1
2481	2	18	1	22.5	1	0	0	1	3	2	2
2482	1	21	1	21.1	1	0	0	0	3	2	2
2483	1	18	1	20.0	1	0	0	0	3	1	2
2485	1	18	1	19.0	1	1	0	0	1	1	1
2488	1	39	2	36.4	3	0	0	0	3	1	2
2489	1	19	1	21.3	1	0	0	0	1	1	1
2490	1	18	1	22.0	1	0	1	1	3	2	1
2491	1	18	1	18.8	1	0	0	0	1	1	1
2492	1	20	1	26.8	2	0	0	0	2	1	1
2493	2	22	1	20.2	1	0	0	0	3	2	1
2494	1	19	1	20.2	1	0	0	0	1	1	1
2495	1	21	1	29.6	2	1	0	0	2	1	2
2496	1	20	1	21.4	1	1	1	0	2	1	2
2497	1	23	1	35.7	3	1	0	0	3	1	1
2498	1	19	1	24.9	1	0	1	0	1	1	1
2499	1	20	1	26.7	2	1	0	0	2	1	1
2500	2	26	2	35.9	3	0	0	0	1	1	1
2501	1	20	1	20.4	1	0	1	0	2	1	3
2502	1	18	1	20.7	1	0	0	0	2	2	1
2503	1	22	1	16.6	1	0	0	0	3	1	1
2507	1	19	1	23.3	1	1	1	0	2	1	1
2508	1	25	1	28.7	2	1	0	0	3	1	1
2509	1	21	1	20.6	1	0	0	0	2	2	1
2510	1	21	1	22.7	1	0	0	0	2	1	2
2511	1	20	1	22.5	1	1	1	0	3	1	2
2514	1	18	1	18.4	1	0	0	0	1	1	2
2516	2	20	1	27.2	2	0	0	0	2	2	1
2517	1	19	1	25.0	1	1	0	0	3	1	1
2519	1	21	1	27.4	2	1	0	0	2	1	1
2521	1	20	1	16.3	1	1	0	0	2	1	1
2523	2	19	1	19.6	1	0	0	0	3	2	1
2524	1	25	1	21.0	1	0	0	1	3	2	1
2526	1	19	1	17.1	1	1	0	0	3	1	1
2527	2	20	1	25.3	2	0	0	1	2	1	1
2529	1	24	1	20.0	1	0	0	0	1	2	1
2532	2	23	1	27.2	2	0	0	1	2	1	2
2533	1	20	1	21.5	1	0	0	0	3	1	1
2535	1	25	1	19.7	1	0	0	0	3	1	2
2537	1	19	1	18.9	1	0	0	1	2	1	3

2538	2	19	1	21.2	1	1	0	0	2	1	1
2539	1	21	1	23.7	1	1	0	0	3	1	1
2540	1	22	1	21.0	1	0	0	1	3	2	1
2541	2	18	1	20.8	1	0	0	1	3	1	2
2542	2	29	2	24.4	1	0	0	0	1	1	3
2546	1	21	1	19.4	1	0	0	0	3	1	1
2548	1	21	1	16.0	1	0	0	0	2	1	1
2549	1	18	1	19.3	1	1	0	0	2	1	1
2550	1	23	1	18.3	1	1	0	0	2	2	2
2551	1	22	1	27.2	2	0	0	0	3	1	3
2552	1	20	1	17.6	1	1	0	0	3	1	3
2553	1	20	1	21.5	1	0	0	0	3	1	1
2554	2	22	1	27.3	2	0	1	0	3	2	1
2555	2	19	1	21.4	1	0	0	0	2	1	1
2556	1	21	1	17.7	1	0	0	0	2	1	1
2557	1	22	1	22.2	1	0	0	0	2	1	2
2558	2	21	1	23.9	1	0	0	0	3	1	1
2559	1	19	1	22.4	1	0	0	1	2	1	1
2560	2	20	1	22.1	1	0	1	1	2	2	1
2561	1	19	1	25.4	2	1	1	0	2	1	1
2562	2	20	1	24.5	1	0	0	0	3	2	2
2564	2	22	1	29.3	2	1	0	0	2	2	1
2566	2	21	1	20.8	1	0	0	0	1	1	1
2568	2	20	1	22.6	1	0	1	0	3	2	2
2569	1	19	1	21.0	1	0	0	0	3	1	1
2570	1	20	1	19.1	1	0	0	0	3	1	1
2571	1	24	1	23.4	1	0	0	0	2	1	2
2572	1	20	1	23.8	1	0	0	1	2	1	2
2573	2	22	1	23.1	1	1	1	0	2	1	2
2574	2	22	1	24.8	1	1	1	1	2	1	3
2575	1	20	1	26.9	2	1	1	0	3	2	2
2579	1	23	1	33.5	3	0	0	0	2	1	1
2580	1	23	1	23.4	1	0	0	1	3	1	3
2581	1	20	1	19.2	1	1	0	0	3	1	1
2582	2	21	1	22.9	1	0	1	0	3	2	2
2583	1	20	1	23.1	1	0	0	0	2	1	1
2584	2	20	1	25.8	2	0	0	1	2	2	3
2586	2	23	1	22.5	1	0	0	0	3	2	1
2587	1	24	1	31.1	3	0	0	0	3	1	1
2588	1	19	1	22.5	1	0	0	1	3	1	1
2589	1	20	1	20.4	1	0	0	0	2	1	1
2590	1	19	1	24.1	1	0	0	0	2	1	1
2591	1	26	2	26.3	2	0	0	1	3	1	2
2592	1	34	2	26.2	2	0	0	0	3	1	1
2593	1	29	2	34.2	3	0	1	0	1	1	2
2594	1	19	1	21.6	1	0	0	0	2	1	1
2595	1	21	1	20.8	1	0	0	0	2	1	1
2596	2	19	1	18.3	1	0	0	0	3	1	1
2598	1	24	1	18.5	1	1	1	0	2	1	1
2599	1	21	1	18.7	1	1	0	1	3	2	2
2600	2	18	1	27.7	2	0	1	0	3	2	3
2601	1	19	1	23.9	1	0	0	1	2	2	3
2603	2	20	1	18.2	1	0	0	1	3	1	2
2606	1	22	1	23.3	1	0	0	1	2	2	2
2608	1	21	1	21.8	1	0	0	1	1	1	2
2609	1	29	2	27.2	2	0	0	1	3	2	2
2611	1	20	1	21.9	1	0	0	0	2	1	1
2612	1	22	1	21.5	1	1	1	0	3	2	2
2614	1	21	1	25.1	2	0	0	1	2	1	2
2615	1	21	1	24.8	1	0	0	0	2	2	1
2616	1	24	1	23.9	1	0	0	0	3	1	1
2617	2	20	1	19.3	1	0	0	1	2	2	2
2618	2	23	1	25.2	2	1	0	0	2	2	3
2619	1	24	1	21.2	1	0	0	0	3	1	1
2620	1	18	1	24.8	1	0	1	0	3	1	3
2621	1	19	1	22.3	1	1	1	0	3	1	1

2622	1	24	1	29.5	2	0	0	0	3	1	1
2624	2	20	1	25.9	2	1	0	0	2	2	2
2625	1	38	2	21.3	1	0	0	0	3	1	1
2626	1	21	1	30.8	3	1	0	0	1	1	1
2627	1	18	1	18.8	1	0	0	0	2	2	1
2628	1	22	1	25.9	2	0	0	1	3	1	1
2629	2	18	1	18.1	1	0	1	0	2	1	2
2631	2	32	2	25.0	2	0	0	1	3	2	2
2632	2	20	1	23.9	1	0	0	0	2	1	1
2633	2	21	1	24.4	1	0	1	1	2	1	2
2634	1	22	1	19.9	1	0	0	0	3	1	2
2635	2	21	1	17.2	1	0	0	0	2	2	2
2636	2	23	1	27.2	2	1	0	0	3	2	2
2638	2	22	1	17.7	1	1	0	0	2	2	2
2639	1	23	1	43.8	3	0	1	1	2	1	1
2640	1	19	1	24.8	1	0	0	0	3	1	1
2642	2	21	1	21.5	1	0	0	0	2	1	1
2643	1	20	1	21.5	1	0	0	0	3	1	1
2644	1	20	1	18.9	1	1	0	0	2	2	2
2645	1	19	1	20.6	1	0	0	0	3	1	1
2646	1	21	1	19.6	1	1	1	0	3	1	1
2648	1	20	1	23.7	1	0	0	0	2	1	3
2649	1	21	1	19.9	1	0	0	0	3	1	1
2650	1	25	1	21.3	1	0	0	0	2	1	1
2651	1	18	1	20.1	1	0	0	0	2	1	3
2654	1	24	1	23.1	1	0	0	0	2	1	3
2655	1	20	1	30.1	3	0	1	1	3	1	1
2656	1	21	1	16.7	1	1	1	0	2	1	1
2658	2	20	1	32.8	3	0	0	1	3	2	1
2659	2	19	1	21.5	1	1	0	0	2	2	2
2661	2	21	1	18.7	1	0	1	0	2	1	2
2667	2	25	1	23.7	1	1	1	0	2	1	2
2668	2	21	1	24.1	1	0	0	0	2	1	3
2669	2	20	1	26.2	2	1	1	0	3	1	1
2670	1	26	2	21.4	1	0	0	1	2	1	2
2671	1	21	1	21.6	1	0	0	0	3	1	1
2672	1	24	1	24.1	1	0	0	0	3	1	1
2673	1	18	1	17.9	1	1	0	0	3	1	2
2674	1	43	2	33.1	3	0	0	1	3	2	1
2676	1	20	1	18.5	1	1	0	0	3	1	2
2677	2	23	1	21.3	1	1	0	0	3	1	2
2678	1	22	1	23.2	1	1	1	0	3	1	1
2679	1	27	2	24.5	1	0	0	0	3	2	1
2680	2	23	1	24.8	1	0	0	0	2	1	1
2681	2	24	1	25.1	2	1	0	0	2	1	3
2682	2	18	1	22.9	1	0	0	0	2	1	1
2683	2	22	1	20.8	1	1	1	0	3	2	1
2684	2	20	1	21.8	1	0	0	0	2	1	1
2685	1	23	1	18.4	1	1	0	0	2	1	2
2687	2	37	2	23.3	1	0	0	0	2	2	3
2689	1	26	2	21.5	1	0	0	1	3	2	2
2691	2	23	1	27.8	2	1	1	0	3	1	2
2692	2	19	1	21.7	1	0	0	1	3	2	3
2694	2	41	2	24.2	1	0	0	0	2	1	3
2695	1	20	1	23.4	1	1	1	0	2	1	3
2696	1	20	1	23.9	1	1	1	0	2	1	1
2697	1	23	1	21.4	1	0	1	0	2	2	1
2698	2	20	1	22.5	1	1	1	0	3	2	2
2699	2	22	1	27.1	2	1	0	0	3	2	2
2700	1	21	1	20.7	1	1	1	0	2	1	1
2701	1	32	2	24.7	1	1	1	0	3	1	2
2702	1	19	1	36.0	3	0	0	0	2	1	1
2703	1	22	1	25.2	2	0	0	0	2	1	1
2707	1	54	2	29.4	2	1	0	0	2	2	2
2708	1	20	1	18.3	1	0	0	0	2	1	1
2710	1	24	1	34.3	3	1	1	0	3	1	1

2711	1	25	1	20.1	1	0	0	0	2	2	1
2715	1	19	1	22.5	1	1	0	0	2	1	2
2716	1	22	1	22.3	1	0	0	0	3	1	2
2717	1	24	1	21.4	1	1	0	1	2	1	3
2719	2	36	2	17.7	1	0	0	0	2	1	1
2720	1	22	1	23.3	1	1	1	0	3	1	1
2721	1	18	1	19.0	1	0	0	0	3	2	2
2722	1	18	1	26.9	2	0	1	1	2	1	2
2723	1	42	2	21.2	1	1	0	1	3	2	2
2724	1	23	1	22.3	1	1	0	0	3	2	1
2725	2	21	1	21.9	1	0	0	0	3	2	1
2726	1	35	2	27.7	2	0	0	0	2	1	1
2727	1	20	1	22.1	1	0	0	0	2	1	2
2728	2	20	1	16.7	1	0	0	0	2	1	1
2729	2	31	2	31.2	3	1	0	0	2	1	1
2730	2	23	1	25.9	2	1	1	0	2	1	1
2731	1	19	1	23.4	1	0	1	0	2	1	2
2733	1	21	1	22.0	1	1	0	0	3	1	1
2735	2	20	1	20.1	1	1	0	1	3	2	1
2736	2	20	1	23.3	1	1	1	0	2	1	3
2738	2	21	1	24.1	1	1	0	0	3	2	2
2739	1	20	1	22.6	1	0	0	0	2	2	2
2741	2	24	1	19.4	1	0	1	0	2	1	2
2742	1	21	1	26.6	2	0	0	0	1	1	2
2743	1	29	2	31.6	3	0	0	0	3	2	3
2744	1	22	1	23.9	1	0	0	0	2	1	2
2745	1	23	1	27.8	2	1	1	1	3	1	2
2749	1	27	2	20.3	1	0	0	0	2	2	2
2750	2	43	2	33.5	3	1	0	1	2	2	2
2751	1	20	1	16.9	1	1	0	0	3	1	1
2753	2	18	1	22.3	1	1	0	0	2	1	1
2754	1	24	1	29.1	2	0	0	0	3	1	1
2756	2	19	1	28.7	2	0	0	1	2	1	2
2757	2	21	1	22.9	1	1	1	0	3	2	1
2758	1	49	2	30.1	3	0	0	0	1	2	1
2759	1	21	1	20.5	1	1	1	0	1	1	1
2760	1	19	1	17.6	1	0	0	0	2	1	1
2761	2	31	2	24.3	1	0	1	0	3	1	2
2762	1	21	1	16.7	1	1	1	0	2	1	1
2763	1	23	1	27.4	2	0	0	1	3	2	1
2764	2	20	1	21.2	1	0	0	0	1	2	1
2765	1	23	1	30.8	3	0	0	0	3	1	2
2766	2	19	1	19.8	1	0	0	0	2	2	1
2767	1	19	1	22.0	1	0	0	1	2	1	3
2769	1	53	2	23.4	1	0	0	1	2	2	2
2770	2	30	2	26.2	2	0	0	0	1	2	1
2771	1	27	2	19.3	1	0	0	0	2	2	1
2772	1	24	1	22.0	1	1	0	1	2	1	2
2773	1	24	1	24.0	1	0	0	0	3	1	1
2776	2	21	1	25.2	2	1	1	0	2	1	1
2777	2	22	1	34.0	3	0	0	0	2	1	2
2782	1	36	2	27.6	2	0	0	0	2	1	1
2783	1	24	1	27.3	2	0	0	0	3	1	1
2784	2	23	1	20.5	1	1	0	0	2	1	1
2786	1	46	2	22.0	1	0	0	1	1	1	1
2787	1	18	1	22.6	1	0	0	0	3	1	1
2788	1	20	1	26.0	2	0	0	1	3	1	2
2789	1	21	1	21.9	1	1	1	1	3	2	1
2790	1	25	1	19.1	1	0	0	0	3	1	2
2792	1	24	1	22.0	1	0	0	0	3	2	1
2793	1	18	1	18.1	1	0	1	0	3	1	3
2794	1	36	2	20.1	1	1	1	0	3	1	1
2795	1	20	1	22.1	1	1	1	0	2	1	1
2797	2	25	1	25.8	2	1	0	0	1	2	2
2798	2	20	1	22.1	1	0	1	0	2	2	2
2799	1	20	1	20.5	1	0	0	0	3	2	1

2800	1	20	1	21.0	1	1	1	1	3	1	2
2801	1	23	1	27.1	2	0	0	0	3	1	1
2802	2	20	1	20.1	1	0	0	1	2	2	2
2803	1	24	1	22.7	1	0	0	1	2	1	3
2804	1	20	1	20.2	1	0	0	0	1	1	2
2806	1	19	1	19.3	1	0	0	0	3	1	2
2807	2	22	1	21.0	1	0	0	0	2	2	2
2810	2	19	1	23.9	1	1	1	1	2	2	2
2811	2	21	1	19.0	1	1	0	1	2	2	3
2812	1	22	1	21.8	1	0	0	0	2	1	1
2813	1	22	1	27.5	2	1	1	1	1	1	2
2814	2	32	2	25.4	2	0	1	0	3	2	2
2815	1	18	1	21.3	1	0	0	0	3	1	1
2817	1	19	1	29.1	2	0	1	0	3	1	2
2818	1	22	1	21.3	1	0	0	0	3	1	1
2819	1	23	1	26.0	2	1	1	0	2	1	1
2820	1	20	1	22.6	1	1	0	0	3	1	2
2821	2	29	2	19.6	1	0	1	0	3	1	1
2822	1	19	1	22.6	1	0	0	1	2	1	1
2823	1	22	1	19.3	1	0	0	0	3	1	1
2826	1	19	1	21.1	1	0	0	0	2	1	2
2827	1	20	1	24.6	1	1	1	0	2	1	1
2828	2	23	1	26.9	2	1	1	0	2	1	1
2830	1	21	1	17.5	1	1	0	0	2	1	1
2831	1	18	1	20.3	1	0	0	0	3	1	1
2833	1	29	2	23.1	1	0	0	0	2	1	1
2834	1	22	1	23.5	1	0	0	0	3	1	1
2835	2	20	1	29.3	2	1	1	0	3	2	1
2836	1	20	1	21.8	1	1	0	0	3	2	1
2837	1	18	1	20.2	1	1	1	0	3	1	1
2839	2	26	2	32.7	3	0	0	0	2	2	2
2840	1	19	1	26.8	2	0	0	0	2	1	1
2841	1	19	1	24.8	1	0	1	0	2	1	1
2842	2	21	1	22.5	1	1	1	0	2	1	2
2844	2	20	1	24.9	1	1	1	0	1	1	2
2845	1	22	1	27.1	2	1	0	0	2	2	1
2846	1	29	2	28.3	2	0	0	0	1	1	1
2847	1	33	2	18.8	1	0	0	0	1	1	1
2848	1	24	1	19.0	1	0	0	0	3	1	1
2849	1	25	1	19.3	1	0	0	0	2	1	1
2850	1	19	1	35.0	3	0	0	0	2	1	1
2851	2	18	1	18.3	1	0	0	0	3	1	3
2852	2	23	1	21.2	1	0	1	0	3	2	1
2854	2	21	1	26.2	2	0	0	0	3	1	2
2855	2	23	1	21.7	1	1	1	1	2	1	1
2856	2	21	1	27.2	2	1	1	0	3	1	2
2857	2	29	2	30.5	3	0	0	0	3	2	2
2859	1	28	2	27.7	2	0	1	0	2	1	1
2860	1	23	1	22.1	1	0	0	0	2	1	2
2861	1	21	1	23.7	1	0	0	0	3	1	1
2862	1	20	1	21.9	1	0	0	0	3	2	2
2863	1	23	1	19.1	1	0	0	0	2	1	2
2864	1	20	1	24.8	1	0	0	0	3	2	1
2869	2	23	1	28.7	2	1	0	0	3	2	1
2870	1	25	1	30.0	2	1	1	0	2	1	2
2871	1	32	2	25.5	2	0	0	0	2	1	1
2872	1	22	1	25.1	2	1	1	0	3	1	2
2873	1	23	1	30.5	3	1	1	0	3	1	1
2874	1	20	1	23.7	1	0	0	0	3	1	3
2876	2	20	1	24.6	1	0	0	0	3	1	2
2877	1	18	1	27.7	2	1	1	1	2	2	1
2878	1	19	1	18.7	1	1	1	0	2	1	2
2880	1	26	2	16.7	1	0	0	0	3	1	1
2881	1	23	1	27.5	2	0	1	0	2	1	1
2882	1	18	1	23.3	1	0	0	0	2	2	1
2883	1	20	1	26.0	2	0	0	0	3	2	2

2884	2	19	1	18.1	1	0	0	0	2	2	1
2886	1	18	1	23.8	1	0	0	0	3	1	1
2887	2	19	1	27.4	2	0	0	0	3	1	2
2888	1	18	1	34.0	3	0	1	0	2	1	1
2890	1	19	1	22.0	1	0	0	0	2	1	1
2891	1	19	1	30.4	3	0	0	0	3	1	1
2892	1	24	1	42.7	3	0	0	0	2	1	1
2893	1	32	2	20.7	1	0	0	0	2	1	2
2894	1	50	2	17.6	1	0	0	1	2	1	1
2895	1	19	1	19.5	1	1	1	0	3	1	3
2896	1	22	1	26.0	2	0	1	0	3	1	1
2898	1	18	1	29.4	2	0	0	0	3	1	1
2900	1	21	1	25.1	2	1	0	0	2	1	2
2901	2	39	2	25.0	2	0	0	0	3	2	1
2902	1	20	1	22.6	1	0	0	0	2	2	2
2904	2	22	1	21.6	1	0	0	0	3	2	1
2905	1	19	1	22.5	1	0	0	1	3	1	1
2906	2	20	1	22.5	1	0	0	1	3	1	2
2907	2	22	1	19.7	1	1	1	1	2	1	3
2908	1	18	1	18.5	1	0	0	0	3	2	1
2911	1	20	1	25.7	2	0	0	1	3	1	1
2912	2	21	1	24.0	1	1	1	0	3	1	1
2913	2	19	1	26.2	2	1	1	0	2	1	1
2914	1	45	2	21.8	1	0	0	0	3	2	2
2915	1	23	1	22.3	1	0	1	0	1	1	1
2916	1	20	1	25.4	2	0	0	0	2	1	1
2917	1	43	2	27.5	2	0	0	0	2	1	1
2918	1	24	1	32.6	3	0	1	0	2	1	1
2919	1	30	2	22.3	1	0	0	0	2	1	3
2920	1	19	1	18.7	1	0	0	1	3	1	1
2921	1	20	1	19.5	1	1	1	0	3	1	2
2922	2	21	1	38.7	3	0	0	1	1	1	3
2923	1	34	2	20.8	1	0	0	1	3	2	2
2924	1	20	1	20.3	1	1	0	0	3	1	2
2925	1	34	2	22.2	1	0	0	0	2	2	1
2926	1	25	1	16.4	1	0	0	0	2	1	1
2927	1	18	1	19.0	1	0	0	0	1	1	2
2928	2	43	2	28.4	2	0	0	1	2	2	1
2929	2	22	1	27.7	2	1	0	0	3	2	2
2930	1	23	1	32.6	3	1	1	0	3	1	2
2932	1	20	1	22.2	1	1	1	1	2	1	1
2933	2	20	1	27.0	2	1	0	0	3	2	1
2937	2	21	1	23.7	1	0	0	0	2	1	1
2939	1	26	2	28.7	2	0	0	1	3	2	1
2940	1	20	1	34.9	3	1	0	0	3	1	1
2941	2	36	2	24.2	1	1	1	0	2	2	2
2942	1	51	2	26.0	2	0	0	0	1	1	1
2944	1	20	1	24.1	1	1	0	0	2	1	1
2945	1	31	2	19.0	1	1	0	1	3	2	2
2946	1	21	1	19.0	1	1	0	0	3	1	2
2947	2	21	1	21.5	1	0	0	0	3	1	1
2948	2	20	1	30.9	3	1	0	0	2	2	3
2950	2	21	1	24.4	1	0	0	0	3	2	2
2951	1	19	1	21.8	1	0	0	0	2	2	1
2952	1	21	1	19.7	1	0	0	0	2	1	2
2953	1	18	1	14.9	1	0	0	0	3	2	1
2955	1	20	1	16.7	1	0	0	0	3	1	2
2956	1	22	1	24.1	1	0	0	0	3	2	1
2957	1	19	1	23.1	1	0	0	1	3	1	1
2958	1	20	1	20.0	1	1	0	0	2	1	1
2959	1	25	1	28.7	2	0	0	0	2	1	1
2960	1	21	1	28.0	2	1	0	0	2	1	3
2962	1	50	2	29.6	2	0	0	1	2	1	1
2963	2	18	1	20.8	1	1	1	1	3	2	3
2964	1	18	1	22.3	1	0	0	0	1	1	1
2965	1	21	1	21.3	1	0	0	0	3	2	1

2966	1	22	1	20.7	1	0	0	0	1	1	2
2967	2	18	1	22.1	1	0	0	0	2	2	1
2968	1	24	1	17.5	1	0	0	0	3	1	1
2969	1	21	1	22.7	1	1	1	0	2	1	1
2970	2	19	1	23.1	1	0	0	1	3	2	1
2971	1	21	1	26.6	2	0	0	0	1	1	2
2972	1	22	1	21.9	1	0	1	1	3	1	2
2973	1	23	1	22.9	1	0	0	1	3	1	2
2974	1	24	1	18.8	1	0	0	1	2	1	1
2977	1	21	1	20.6	1	0	0	0	3	1	2
2978	1	26	2	23.3	1	1	1	0	3	2	1
2979	2	18	1	20.1	1	0	1	0	3	2	3
2980	1	22	1	23.2	1	0	0	0	3	1	2
2981	1	22	1	23.8	1	0	0	1	2	2	2
2982	1	26	2	31.6	3	0	0	0	3	1	2
2983	1	19	1	21.2	1	0	0	0	3	1	2
2984	1	26	2	19.5	1	1	0	1	3	1	1
2985	1	39	2	27.7	2	1	0	0	3	1	1
2987	2	21	1	17.6	1	0	0	1	2	2	2
2988	1	23	1	19.5	1	0	0	0	2	1	2
2989	2	21	1	22.4	1	1	1	0	3	2	1
2990	1	21	1	23.0	1	0	0	1	1	1	1
2991	2	21	1	27.2	2	1	0	0	2	1	1
2993	1	23	1	22.3	1	0	0	0	3	1	1
2995	1	23	1	18.6	1	0	0	0	2	1	2
2996	1	22	1	22.2	1	1	1	0	3	1	1
2997	2	21	1	26.3	2	0	0	0	3	2	2
2998	1	24	1	24.7	1	0	0	0	3	2	1
2999	1	23	1	19.1	1	0	0	0	3	1	2
3000	2	22	1	27.0	2	1	0	0	2	1	2
3001	2	21	1	25.2	2	0	0	0	2	2	3
3002	1	19	1	18.3	1	1	1	0	2	1	2
3003	1	21	1	19.9	1	0	0	0	3	1	2
3004	1	19	1	22.9	1	0	0	0	3	2	2
3005	1	24	1	22.8	1	1	0	0	3	1	2
3006	1	22	1	28.4	2	0	0	0	2	2	2
3007	1	21	1	23.1	1	0	0	0	2	2	3
3008	1	21	1	22.7	1	0	0	0	3	1	1
3010	1	21	1	21.2	1	0	0	0	3	2	1
3011	2	21	1	21.1	1	0	0	0	3	1	3
3012	1	52	2	29.0	2	0	0	0	2	2	3
3013	2	21	1	31.4	3	0	0	0	2	1	1
3015	2	20	1	21.6	1	0	0	0	3	2	1
3016	1	21	1	30.1	3	0	0	0	3	1	1
3017	1	24	1	20.3	1	0	0	0	1	1	2
3019	2	19	1	31.2	3	1	1	0	2	1	1
3020	1	21	1	35.0	3	0	1	0	3	1	2
3021	1	22	1	21.6	1	1	0	0	2	1	2
3022	1	23	1	25.7	2	0	0	0	3	1	1
3023	1	20	1	24.0	1	0	0	0	1	1	1
3026	1	29	2	22.1	1	1	0	1	3	1	2
3028	2	19	1	24.2	1	0	0	0	2	1	1
3029	2	20	1	28.3	2	0	1	0	1	1	3
3030	2	20	1	26.9	2	1	0	0	2	1	2
3031	1	20	1	22.3	1	1	1	1	3	2	1
3032	1	18	1	27.3	2	0	0	0	3	1	2
3033	1	23	1	25.4	2	1	0	0	3	1	3
3034	1	19	1	31.2	3	0	0	0	3	1	1
3035	2	20	1	27.3	2	0	1	0	2	2	1
3036	2	22	1	18.4	1	1	1	1	2	1	2
3037	1	24	1	24.0	1	1	1	0	2	1	1
3038	1	21	1	20.1	1	0	0	0	2	1	2
3039	1	22	1	17.9	1	0	0	0	3	1	2
3041	1	21	1	28.0	2	1	0	0	3	1	1
3042	2	22	1	24.3	1	1	0	0	3	1	2
3043	1	23	1	24.4	1	0	0	0	2	1	2

3045	1	32	2	34.9	3	0	0	0	3	1	1
3046	2	26	2	21.6	1	1	0	0	3	1	1
3047	1	21	1	33.9	3	0	0	0	3	2	1
3048	2	22	1	22.2	1	0	0	0	3	2	2
3049	1	19	1	25.1	2	1	1	0	3	1	2
3050	1	24	1	24.2	1	0	1	1	2	2	2
3051	2	22	1	24.0	1	0	0	1	3	2	3
3052	1	20	1	27.0	2	1	1	1	1	1	3
3053	1	21	1	20.8	1	1	1	1	3	2	3
3054	2	20	1	19.0	1	1	1	0	2	1	1
3056	2	19	1	19.7	1	1	1	0	3	2	2
3058	2	18	1	26.8	2	0	0	0	2	1	2
3059	2	21	1	21.9	1	0	1	0	2	2	2
3060	1	26	2	21.9	1	0	0	0	2	1	1
3061	1	22	1	22.4	1	0	0	0	1	2	1
3062	1	19	1	26.6	2	1	1	0	3	2	2
3063	1	19	1	26.7	2	1	0	0	2	1	1
3065	1	25	1	23.5	1	0	0	0	1	1	1
3066	2	19	1	25.4	2	1	1	0	3	2	2
3067	1	25	1	34.5	3	0	0	0	2	1	1
3069	1	19	1	21.7	1	0	1	0	1	1	3
3070	2	20	1	21.1	1	0	0	0	3	2	2
3071	1	26	2	22.1	1	1	0	0	3	2	2
3073	1	20	1	23.9	1	1	1	0	3	1	2
3074	1	22	1	20.7	1	0	0	0	3	1	1
3076	1	19	1	25.5	2	0	0	0	2	1	2
3078	2	19	1	17.8	1	1	1	0	3	1	1
3081	2	23	1	23.0	1	1	0	0	2	2	2
3082	1	20	1	16.5	1	0	1	1	2	1	3
3083	1	22	1	20.8	1	0	0	0	1	1	1
3084	2	23	1	29.1	2	1	1	0	2	1	2
3085	1	18	1	23.9	1	0	0	0	2	1	1
3086	1	21	1	19.5	1	0	0	0	2	1	1
3087	1	21	1	20.2	1	0	0	1	2	1	2
3088	1	19	1	20.8	1	0	0	0	3	2	2
3089	1	19	1	23.8	1	0	0	0	3	1	3
3090	1	20	1	21.8	1	0	0	0	3	1	3
3091	1	18	1	23.5	1	1	1	0	2	1	2
3092	2	23	1	20.7	1	1	0	0	1	1	2
3093	1	25	1	25.1	2	0	1	0	3	1	2
3094	1	22	1	19.6	1	0	0	0	2	1	1
3095	2	23	1	22.5	1	0	0	0	3	2	3
3097	1	19	1	16.8	1	0	0	1	2	2	2
3099	1	33	2	27.6	2	0	0	1	2	1	2
3100	1	37	2	30.5	3	1	1	0	3	1	1
3104	1	20	1	24.8	1	0	0	0	3	1	2
3105	2	19	1	25.2	2	0	0	0	2	2	1
3106	1	23	1	29.1	2	1	1	0	2	1	1
3107	1	21	1	24.2	1	0	0	0	3	2	2
3108	1	21	1	30.4	3	1	1	0	2	1	1
3109	1	27	2	16.9	1	0	0	1	3	1	1
3110	2	28	2	25.4	2	0	0	0	1	1	2
3111	2	20	1	19.6	1	0	0	1	2	2	1
3113	1	24	1	34.2	3	0	1	0	2	1	1
3114	2	20	1	24.9	1	0	0	0	3	1	1
3116	2	22	1	23.9	1	0	0	0	3	1	2
3117	1	22	1	19.1	1	0	0	0	2	1	1
3120	1	18	1	21.0	1	0	0	1	2	1	1
3121	1	21	1	34.6	3	0	1	0	2	1	1
3122	1	20	1	19.8	1	0	0	1	2	1	3
3123	1	21	1	22.9	1	0	0	0	2	1	1
3124	2	22	1	22.5	1	0	1	0	2	2	2
3125	1	20	1	19.8	1	0	0	0	2	1	1
3126	1	22	1	19.8	1	0	0	0	3	2	1
3127	1	20	1	20.3	1	1	0	1	2	1	3
3128	1	53	2	22.0	1	0	0	1	1	1	1

3129	1	22	1	31.1	3	0	0	0	1	1	1
3130	2	18	1	16.8	1	0	1	0	3	1	2
3131	2	21	1	25.9	2	1	0	1	3	2	3
3132	1	25	1	25.2	2	1	1	1	3	2	2
3133	1	24	1	18.7	1	0	0	0	2	1	1
3134	1	20	1	27.8	2	1	0	0	1	1	1
3135	2	22	1	22.2	1	1	0	1	2	1	2
3136	1	22	1	33.3	3	0	0	1	2	2	2
3137	1	19	1	18.1	1	0	0	0	3	2	1
3138	1	21	1	24.5	1	0	0	0	3	1	1
3139	1	21	1	37.6	3	0	1	0	2	1	1
3140	1	20	1	21.5	1	0	1	0	3	1	2
3142	1	20	1	15.9	1	0	0	0	3	2	1
3143	1	21	1	17.0	1	1	0	1	2	1	2
3144	1	24	1	38.1	3	0	0	1	2	1	3
3145	2	21	1	20.6	1	0	0	1	2	2	2
3147	2	20	1	21.8	1	1	1	0	3	1	3
3148	1	21	1	24.2	1	0	0	0	1	1	2
3149	1	21	1	25.7	2	1	0	0	3	1	1
3150	2	22	1	28.0	2	0	0	0	3	1	1
3151	2	23	1	20.2	1	1	1	0	3	1	2
3153	1	23	1	23.7	1	0	0	0	3	1	1
3154	2	18	1	18.3	1	0	0	0	2	1	1
3155	2	20	1	26.3	2	0	0	0	3	2	1
3156	2	21	1	20.5	1	0	0	0	3	2	2
3157	2	19	1	33.1	3	0	1	0	3	1	1
3158	1	23	1	27.3	2	0	0	0	3	2	1
3160	1	18	1	19.8	1	0	0	1	3	1	1
3161	2	26	2	21.6	1	1	1	0	1	1	1
3162	1	18	1	21.1	1	0	0	0	3	2	1
3163	1	21	1	29.6	2	0	0	0	2	1	1
3164	1	20	1	23.2	1	1	0	0	3	1	3
3165	2	25	1	21.0	1	1	1	1	1	1	1
3166	1	18	1	27.7	2	0	0	1	1	1	2
3167	2	20	1	17.2	1	0	0	1	2	1	1
3168	1	29	2	21.2	1	0	0	1	1	2	3
3169	2	22	1	34.1	3	1	1	0	2	1	1
3170	1	20	1	30.0	3	0	1	1	1	2	1
3171	1	23	1	21.7	1	0	0	0	1	1	1
3172	1	20	1	25.9	2	0	0	0	3	2	1
3173	1	20	1	35.6	3	1	1	0	1	1	2
3174	1	20	1	28.1	2	0	0	0	2	1	1
3175	2	44	2	33.9	3	0	0	0	1	2	2
3176	1	22	1	16.0	1	0	0	0	2	1	1
3177	2	47	2	28.4	2	0	0	1	2	2	2
3178	1	21	1	22.9	1	0	1	0	2	1	2
3179	1	26	2	22.0	1	1	1	0	3	1	3
3180	2	22	1	22.8	1	0	1	1	3	1	1
3182	1	20	1	20.1	1	0	0	0	3	2	1
3183	1	24	1	22.9	1	1	0	0	3	2	2
3184	1	18	1	19.9	1	0	0	0	2	2	2
3186	1	19	1	26.4	2	0	0	1	3	1	2
3187	1	20	1	23.9	1	1	1	0	2	1	1
3188	2	22	1	21.3	1	1	0	0	2	1	1
3189	1	20	1	19.6	1	0	0	1	3	2	2
3190	1	18	1	24.4	1	0	1	0	3	1	1
3193	1	20	1	23.5	1	1	1	0	3	1	1
3194	2	23	1	22.8	1	0	0	0	2	1	2
3197	1	20	1	19.3	1	0	0	0	3	1	2
3198	2	21	1	28.4	2	1	1	0	1	1	1
3199	1	18	1	31.2	3	0	0	0	2	1	2
3200	1	32	2	25.6	2	0	0	0	2	1	2
3201	1	42	2	26.1	2	1	0	0	1	1	1
3202	1	18	1	21.8	1	0	0	0	2	1	3
3206	1	19	1	17.7	1	1	1	1	3	1	1
3207	1	37	2	23.7	1	0	0	0	3	1	1

3208	2	27	2	24.8	1	1	1	1	2	2	3
3209	2	20	1	27.8	2	0	0	1	3	2	1
3210	2	28	2	34.7	3	1	1	0	2	1	3
3212	1	20	1	26.2	2	1	0	0	3	1	1
3213	1	22	1	19.8	1	0	1	1	2	1	3
3214	1	22	1	26.6	2	1	1	0	3	1	1
3217	2	25	1	27.0	2	0	1	0	3	2	2
3218	1	19	1	29.1	2	0	0	0	3	2	1
3219	1	24	1	21.5	1	1	1	1	3	1	2
3224	1	40	2	23.0	1	1	0	0	2	1	2
3225	1	21	1	20.5	1	0	0	0	3	1	1
3226	1	19	1	18.6	1	0	0	0	3	2	2
3230	1	39	2	25.7	2	0	0	0	3	1	1
3231	1	19	1	20.8	1	0	0	0	2	1	2
3232	2	20	1	24.0	1	1	1	0	3	1	1
3233	2	21	1	36.2	3	1	1	0	3	1	1
3235	2	22	1	19.4	1	0	0	0	3	2	1
3236	1	21	1	20.2	1	0	0	0	3	2	1
3238	2	19	1	30.7	3	0	0	0	3	1	1
3239	1	20	1	21.3	1	0	0	1	1	1	1
3240	2	22	1	20.4	1	0	1	0	2	1	1
3241	1	18	1	17.9	1	1	0	1	2	1	2
3242	1	21	1	18.0	1	0	0	0	2	2	1
3243	1	18	1	21.8	1	1	0	0	2	2	2
3244	2	24	1	25.1	2	0	1	0	2	1	1
3245	1	21	1	27.3	2	0	0	0	3	1	1
3246	1	22	1	20.9	1	1	0	0	2	1	2
3248	2	22	1	21.6	1	1	1	0	3	2	1
3250	1	24	1	18.7	1	0	0	0	3	1	1
3251	1	22	1	17.7	1	1	0	0	1	2	1
3252	1	26	2	20.4	1	0	0	0	2	2	2
3254	1	22	1	26.3	2	1	0	1	3	1	1
3255	1	18	1	20.2	1	0	0	1	3	2	2
3256	1	44	2	34.4	3	0	0	0	1	1	1
3257	1	21	1	33.4	3	1	0	0	2	1	2
3258	2	19	1	18.6	1	1	1	0	2	1	1
3259	1	18	1	32.0	3	0	0	0	3	1	1
3260	1	18	1	16.6	1	0	0	0	3	1	1
3261	1	20	1	23.6	1	1	1	1	3	1	2
3262	1	20	1	32.9	3	1	1	1	2	1	2
3264	2	40	2	26.1	2	0	0	0	3	2	1
3265	1	20	1	19.7	1	0	0	1	2	1	3
3266	1	20	1	20.7	1	1	0	0	2	1	2
3267	1	20	1	24.0	1	0	0	0	1	1	1
3268	2	24	1	18.4	1	1	0	0	3	1	3
3269	1	19	1	20.8	1	1	0	0	2	1	1
3270	1	20	1	20.7	1	0	0	0	2	2	1
3271	1	19	1	25.1	2	0	1	0	2	1	1
3272	1	24	1	26.6	2	0	0	0	3	1	1
3273	2	19	1	24.2	1	1	1	1	3	1	2
3274	2	22	1	32.4	3	0	0	0	3	1	1
3275	2	20	1	19.9	1	0	1	0	2	1	2
3276	1	21	1	28.0	2	0	1	0	2	1	1
3277	2	20	1	21.3	1	0	0	0	2	2	1
3278	1	19	1	21.6	1	0	0	0	3	2	2
3279	2	19	1	23.1	1	1	1	0	3	1	1
3280	2	23	1	18.5	1	1	1	1	2	1	2
3283	2	21	1	22.0	1	1	0	0	3	2	1
3284	2	20	1	21.6	1	1	1	1	1	1	1
3285	2	18	1	17.7	1	0	0	0	3	1	2
3286	1	37	2	21.3	1	0	0	0	3	1	1
3287	2	22	1	21.6	1	1	0	0	3	2	1
3288	1	19	1	20.9	1	1	0	0	3	2	1
3290	1	25	1	23.3	1	0	1	0	2	1	1
3291	1	19	1	25.7	2	0	0	0	2	2	2
3292	1	19	1	21.2	1	1	1	0	3	1	1

3293	2	24	1	23.8	1	1	1	0	2	2	1
3294	1	21	1	19.8	1	0	0	0	2	2	1
3295	1	21	1	19.4	1	0	1	0	2	1	2
3297	1	22	1	29.4	2	1	1	0	2	1	2
3300	2	18	1	22.0	1	1	1	0	2	1	2
3301	2	20	1	20.8	1	1	1	0	3	1	1
3302	1	27	2	18.7	1	1	0	1	3	2	1
3305	2	18	1	26.8	2	1	1	0	1	1	2
3306	2	21	1	23.8	1	0	0	0	3	2	3
3309	1	19	1	27.0	2	1	1	0	2	1	1
3312	1	20	1	21.8	1	1	0	1	2	1	2
3313	1	24	1	19.1	1	0	1	0	2	2	2
3314	1	21	1	25.1	2	0	1	0	2	1	1
3315	1	18	1	21.0	1	0	0	0	3	1	2
3317	2	21	1	20.1	1	0	0	0	3	1	2
3318	1	19	1	17.9	1	0	0	0	3	1	1
3319	2	21	1	22.2	1	1	0	0	2	1	3
3320	1	21	1	19.5	1	1	1	0	2	1	1
3321	1	21	1	26.6	2	1	0	0	2	1	1
3322	1	24	1	26.0	2	0	0	0	3	1	1
3323	1	18	1	19.7	1	0	0	0	3	1	2
3324	2	25	1	23.7	1	1	0	0	3	2	1
3325	1	28	2	21.8	1	1	1	0	2	1	1
3326	2	19	1	22.3	1	1	1	0	1	2	3
3329	1	18	1	21.7	1	0	1	1	2	1	2
3330	1	25	1	34.1	3	1	0	0	1	1	1
3331	1	23	1	19.0	1	1	1	1	3	1	2
3333	1	20	1	21.8	1	0	0	0	3	1	1
3334	1	19	1	23.9	1	1	1	0	3	2	1
3335	1	20	1	20.1	1	0	0	0	2	2	2
3336	1	20	1	21.3	1	1	0	0	3	1	1
3338	1	20	1	17.7	1	0	0	0	2	1	2
3339	1	31	2	28.0	2	1	0	0	3	1	1
3340	2	30	2	43.9	3	1	0	0	1	1	1
3341	1	36	2	23.8	1	1	0	0	1	2	1
3342	1	20	1	17.4	1	0	0	1	3	1	2
3343	1	23	1	22.9	1	0	0	0	1	1	1
3344	1	21	1	19.7	1	0	0	0	3	2	3
3346	1	26	2	19.6	1	1	0	0	3	1	2
3347	1	20	1	29.4	2	0	0	0	2	1	1
3348	2	20	1	25.9	2	0	1	1	2	1	2
3349	2	20	1	25.5	2	0	0	0	2	1	1
3350	1	35	2	26.7	2	1	0	0	2	2	1
3354	1	26	2	31.0	3	0	1	0	2	1	2
3355	2	20	1	19.4	1	0	0	0	3	2	2
3356	1	18	1	21.5	1	0	0	1	2	1	2
3357	2	19	1	22.1	1	0	0	0	3	2	1
3358	1	19	1	18.7	1	0	0	0	3	1	1
3361	2	19	1	23.9	1	0	0	1	2	1	2
3362	2	39	2	22.5	1	1	1	0	3	2	2
3363	2	24	1	17.3	1	0	1	0	2	2	1
3364	1	38	2	39.3	3	0	0	0	2	2	1
3365	1	19	1	22.7	1	1	1	0	3	2	1
3366	1	26	2	25.1	2	0	1	0	1	1	2
3369	2	26	2	21.9	1	1	1	1	2	1	2
3370	2	23	1	24.7	1	1	0	0	2	2	1
3371	1	21	1	19.2	1	0	0	0	2	1	2
3374	1	24	1	36.1	3	1	0	0	1	1	2
3375	1	25	1	27.8	2	0	0	0	1	1	1
3376	1	18	1	30.0	3	0	0	0	2	2	2
3377	1	21	1	19.1	1	0	1	0	2	1	1
3378	1	21	1	19.9	1	0	0	0	2	1	1
3379	2	22	1	24.8	1	1	0	1	3	1	3
3380	1	19	1	20.6	1	1	1	1	2	1	3
3381	1	18	1	30.4	3	1	0	0	2	1	1
3382	1	21	1	23.1	1	1	0	0	2	1	1

3383	2	21	1	17.8	1	0	0	0	3	2	3
3384	1	29	2	34.1	3	0	1	0	3	1	2
3385	1	20	1	20.3	1	0	0	0	3	2	1
3386	1	23	1	21.9	1	1	1	1	3	2	2
3387	1	21	1	23.3	1	0	0	1	3	1	1
3388	1	26	2	19.1	1	0	0	0	1	1	1
3389	1	24	1	22.6	1	0	0	0	2	1	2
3390	1	27	2	24.3	1	0	0	1	2	1	3
3391	2	22	1	22.2	1	0	0	1	2	2	2
3392	1	20	1	19.4	1	0	0	0	3	2	2
3393	2	21	1	22.0	1	1	1	0	2	1	2
3395	2	21	1	21.4	1	1	1	0	2	1	1
3396	1	45	2	22.5	1	0	0	0	1	1	1
3397	1	25	1	18.1	1	0	0	1	2	1	2
3398	1	19	1	23.0	1	1	0	1	3	1	1
3399	2	23	1	27.2	2	0	0	0	3	1	2
3400	2	27	2	25.2	2	1	0	0	3	1	1
3402	2	37	2	26.6	2	1	1	0	1	2	2
3403	2	23	1	25.4	2	1	0	0	3	1	3
3404	2	18	1	22.7	1	1	1	0	3	1	2
3405	1	31	2	26.0	2	1	1	0	2	1	1
3406	2	27	2	22.5	1	0	0	0	1	1	1
3407	2	25	1	27.7	2	1	0	0	3	1	1
3409	2	22	1	23.4	1	0	0	0	2	1	1
3411	2	21	1	27.7	2	0	1	1	2	1	2
3412	1	29	2	28.7	2	0	0	0	2	1	1
3413	2	20	1	34.1	3	0	0	0	2	1	1
3414	2	21	1	15.4	1	0	0	0	2	1	1
3415	1	18	1	21.1	1	0	0	0	3	1	1
3417	1	20	1	19.8	1	0	0	0	2	1	1
3418	1	18	1	19.7	1	0	0	0	3	1	1
3419	1	21	1	18.7	1	0	0	1	2	1	2
3420	1	27	2	27.5	2	0	0	0	3	2	1
3422	1	21	1	22.5	1	1	1	0	2	1	1
3423	1	23	1	18.6	1	0	0	0	2	1	1
3424	1	23	1	20.3	1	0	0	0	1	2	2
3426	1	19	1	17.7	1	0	0	0	1	1	1
3427	2	19	1	21.8	1	0	0	0	3	1	1
3428	2	21	1	17.2	1	0	0	0	2	1	1
3429	1	18	1	20.6	1	0	0	0	2	2	1
3430	2	21	1	26.3	2	1	0	0	3	1	2
3431	1	22	1	21.5	1	1	1	0	2	1	2
3433	2	21	1	35.1	3	0	0	1	1	1	3
3434	2	21	1	29.7	2	0	0	1	2	1	3
3436	2	38	2	31.0	3	1	1	1	2	1	2
3437	1	20	1	23.9	1	1	1	0	1	1	2
3439	2	21	1	23.8	1	0	0	0	2	2	2
3441	1	27	2	26.6	2	1	0	0	2	1	1
3442	2	21	1	37.0	3	1	0	0	3	1	1
3444	1	22	1	18.8	1	1	1	1	1	1	2
3446	1	22	1	24.6	1	0	0	1	2	2	2
3447	2	28	2	23.5	1	0	0	1	3	2	3
3449	1	21	1	20.2	1	1	1	0	3	1	2

ID	POORSLEEP	PSQItotal	PSQICO1	PSQICO2	PSQICO3	PSQICO4	PSQICO5	PSQICO6	PSQICO7
1	0	4	1	0	1	0	1	0	1
2	1	10	2	3	2	0	1	0	2
5	1	6	1	1	1	1	1	0	1
6	1	12	2	2	2	2	2	0	2
7	1	6	1	0	1	0	2	0	2
8	1	15	2	3	2	3	2	1	2
9	0	5	1	1	1	0	1	0	1
10	1	11	2	2	1	3	1	0	2
11	1	9	2	2	1	0	1	0	3
12	1	10	2	3	1	0	3	0	1
13	1	6	2	1	1	0	0	0	2
14	0	3	0	0	1	0	1	0	1
15	0	5	1	0	0	0	2	0	2
16	0	1	0	0	1	0	0	0	0
17	0	5	1	1	1	0	1	0	1
18	1	9	2	2	1	0	1	0	3
19	0	5	1	1	1	0	1	0	1
20	1	12	2	2	3	1	2	1	1
21	1	14	1	3	1	3	2	2	2
24	1	7	2	0	1	0	1	0	3
25	0	4	0	1	0	1	1	0	1
26	0	5	1	1	1	0	1	0	1
27	1	10	2	0	2	2	1	0	3
28	1	7	2	0	2	0	1	0	2
30	1	15	3	3	3	0	2	1	3
31	1	6	1	2	0	0	1	1	1
33	0	5	1	1	1	0	1	1	0
34	0	5	1	1	1	0	1	0	1
35	1	15	2	1	3	0	3	3	3
37	1	8	2	1	2	0	1	0	2
38	1	6	1	1	1	0	1	0	2
39	1	6	1	1	1	1	1	0	1
41	1	9	2	2	2	0	1	0	2
43	1	10	1	2	1	1	1	1	3
44	1	9	2	0	2	0	1	1	3
45	1	9	1	2	1	0	2	1	2
46	1	9	2	2	2	0	2	0	1
47	1	10	2	2	1	0	1	3	1
48	0	5	1	1	0	0	1	0	2
49	1	8	2	2	1	0	2	0	1
50	1	6	1	0	1	0	2	0	2
52	1	9	2	2	1	0	1	2	1
53	1	7	1	1	1	1	1	0	2
54	1	8	1	3	1	1	1	0	1
55	1	14	2	3	2	0	2	2	3
58	1	11	1	2	1	2	2	1	2
59	0	5	1	1	1	0	1	0	1
60	0	5	1	0	1	0	1	1	1
61	0	3	0	0	1	0	1	0	1
62	1	9	2	1	1	0	2	0	3
64	0	5	1	2	1	0	1	0	0
65	1	13	2	3	2	2	3	0	1
66	1	6	1	1	0	0	1	0	3
67	1	13	1	3	2	0	2	3	2
69	0	5	1	1	0	0	2	0	1
71	1	10	1	2	0	0	2	3	2
72	1	11	2	1	1	0	2	2	3
73	0	5	1	0	1	0	1	0	2
75	1	7	2	0	1	0	1	0	3
76	0	5	1	1	1	0	1	0	1
77	1	11	3	1	2	0	2	0	3
78	0	5	1	1	1	0	1	0	1
79	1	11	2	2	2	2	1	0	2
80	1	15	3	3	1	1	2	2	3
81	1	6	1	2	1	0	1	0	1

82	1	11	1	2	2	2	1	0	3
83	1	11	3	2	2	0	1	0	3
84	1	7	1	2	1	0	1	0	2
85	1	6	1	1	0	0	2	0	2
89	1	6	1	1	1	0	1	0	2
90	1	9	1	2	1	0	2	0	3
91	1	9	2	2	1	0	1	1	2
92	1	13	1	1	1	2	2	3	3
93	1	10	2	2	1	0	2	0	3
94	1	12	2	2	2	1	3	0	2
96	0	2	0	0	0	0	1	0	1
97	1	12	2	2	1	0	1	3	3
98	1	14	3	3	2	2	2	0	2
99	0	4	1	1	0	0	1	0	1
102	0	4	1	0	2	0	1	0	0
103	1	8	2	1	1	0	1	0	3
104	1	7	2	1	1	0	1	0	2
105	1	9	2	0	2	1	1	0	3
106	1	7	1	2	1	0	2	0	1
107	1	10	2	3	1	0	1	1	2
108	1	11	3	1	2	1	1	1	2
109	0	4	1	1	0	0	1	0	1
110	1	7	1	1	1	0	1	0	3
111	1	6	1	1	0	0	2	0	2
112	1	9	2	1	2	0	1	0	3
114	1	9	1	1	1	0	2	2	2
115	0	4	1	0	1	0	1	0	1
117	0	5	1	1	0	0	2	0	1
119	0	3	0	1	0	0	1	0	1
120	1	11	2	3	1	0	2	0	3
121	1	10	2	3	2	0	1	0	2
123	1	8	2	2	1	0	1	0	2
124	1	11	1	3	1	1	2	0	3
125	1	7	1	1	1	0	1	0	3
126	1	7	2	1	2	0	1	0	1
127	1	11	2	3	1	1	2	1	1
129	1	16	3	2	2	0	3	3	3
131	0	5	1	1	1	0	1	0	1
132	1	10	1	3	1	2	1	0	2
133	1	10	2	1	3	0	2	0	2
134	1	6	1	1	1	0	1	0	2
135	1	8	1	1	1	2	1	0	2
136	1	12	1	2	1	1	2	3	2
138	0	5	1	1	1	0	1	0	1
140	1	13	2	2	3	3	2	0	1
141	1	9	1	3	1	2	1	0	1
142	1	6	2	1	0	0	1	0	2
143	1	8	1	1	1	1	2	1	1
145	1	9	1	1	2	0	2	0	3
146	1	7	1	1	1	0	2	0	2
148	1	6	1	0	2	0	1	0	2
149	1	11	2	3	1	0	2	1	2
150	0	2	0	0	0	0	1	0	1
151	0	5	0	0	1	0	2	0	2
152	1	7	2	0	1	0	2	0	2
153	1	11	2	3	1	1	2	0	2
154	0	5	1	1	1	0	1	0	1
155	1	7	1	2	1	0	2	0	1
156	1	12	3	3	3	0	1	0	2
157	1	6	2	0	1	0	1	0	2
159	0	4	1	1	1	0	1	0	0
161	1	6	1	1	0	0	2	0	2
163	0	5	1	1	1	0	1	0	1
165	1	10	1	2	2	0	1	3	1
166	1	11	2	2	1	0	2	1	3
167	1	9	2	3	0	0	2	1	1

168	1	13	3	2	3	0	2	0	3
169	1	12	2	2	2	1	2	0	3
170	1	9	2	3	1	1	1	0	1
174	1	9	2	2	2	1	1	0	1
176	0	2	0	0	1	0	1	0	0
177	0	5	1	1	1	0	1	0	1
178	1	11	2	1	1	0	3	2	2
180	1	9	2	2	1	0	2	0	2
183	1	8	1	2	0	0	1	3	1
184	1	8	1	2	1	0	2	0	2
185	1	10	1	1	1	0	2	2	3
186	1	16	3	3	2	2	1	3	2
187	0	4	1	0	1	0	1	0	1
188	1	8	1	2	0	0	2	0	3
189	1	14	2	1	3	0	2	3	3
190	1	8	2	3	1	0	1	0	1
193	1	8	1	3	1	0	1	0	2
195	1	7	1	1	0	0	2	0	3
196	0	3	1	0	0	0	1	0	1
197	1	7	1	0	1	0	2	0	3
198	1	14	3	3	2	1	2	0	3
199	1	9	1	3	1	0	2	1	1
200	0	3	0	0	1	1	1	0	0
202	1	10	2	1	1	0	1	3	2
203	0	5	1	0	1	0	1	0	2
204	0	5	1	0	1	0	1	0	2
205	1	8	1	2	1	1	1	0	2
206	1	8	1	2	1	0	2	0	2
208	0	5	1	1	1	0	1	0	1
209	1	7	2	0	2	0	1	0	2
210	1	7	1	1	3	0	1	0	1
213	0	3	0	1	0	0	1	0	1
214	1	8	2	1	1	0	1	0	3
215	1	17	3	2	3	1	2	3	3
216	1	7	1	0	1	0	2	2	1
217	1	12	2	3	2	2	1	0	2
218	1	10	2	2	2	2	1	0	1
220	1	7	1	1	1	0	1	0	3
221	1	16	3	3	3	0	2	2	3
222	1	15	3	3	1	2	3	0	3
223	1	11	1	3	1	0	1	2	3
224	0	3	0	1	0	0	1	0	1
225	0	4	1	1	0	0	1	0	1
227	1	6	2	0	1	0	1	0	2
228	1	11	2	3	1	0	1	3	1
229	1	10	2	2	0	0	2	2	2
230	1	16	3	3	1	3	2	3	1
231	1	7	1	1	1	0	2	0	2
232	1	12	3	3	1	1	2	0	2
233	1	6	1	2	1	0	1	0	1
235	1	7	1	2	0	0	1	0	3
236	1	10	2	2	1	0	2	0	3
238	1	9	2	2	1	0	2	0	2
239	1	7	1	0	1	0	2	0	3
240	0	5	1	0	1	0	1	0	2
241	1	7	1	1	1	0	2	0	2
242	1	11	2	3	1	0	2	0	3
243	1	9	2	2	1	0	1	0	3
244	1	10	2	2	1	0	2	0	3
245	1	9	1	2	2	0	1	0	3
246	1	11	2	3	2	0	1	0	3
247	1	10	2	1	3	1	1	0	2
248	1	6	2	0	0	0	1	0	3
249	0	5	1	1	1	0	1	0	1
251	0	4	1	0	1	0	1	0	1
252	1	9	2	2	2	2	1	0	0

254	1	9	2	1	2	0	1	0	3
255	1	9	2	2	1	0	3	0	1
256	1	6	1	2	1	0	1	0	1
258	1	12	1	2	1	2	3	1	2
259	1	15	3	2	3	2	2	0	3
260	1	12	1	3	2	1	2	0	3
264	1	15	3	2	2	0	2	3	3
265	1	15	3	3	1	3	2	0	3
267	1	6	1	1	1	0	1	0	2
268	1	13	1	3	0	2	2	3	2
269	1	12	2	2	2	2	1	0	3
270	0	5	1	1	1	0	1	0	1
271	1	8	1	1	1	0	1	3	1
272	0	4	1	1	1	0	0	0	1
273	1	7	1	2	1	0	1	0	2
274	0	5	1	1	1	0	1	0	1
275	1	7	1	1	2	0	1	0	2
277	1	7	1	1	1	0	1	0	3
278	1	11	2	3	1	1	1	0	3
279	1	13	2	3	0	1	3	1	3
280	1	13	2	3	2	1	2	0	3
281	1	8	2	2	2	0	1	0	1
282	1	9	3	0	3	0	1	0	2
284	0	4	1	0	1	0	1	0	1
285	1	6	1	2	1	0	1	0	1
286	0	5	1	0	1	0	1	0	2
288	1	19	3	2	3	3	2	3	3
289	1	6	1	1	0	0	2	0	2
291	0	4	1	0	0	0	1	0	2
293	1	6	1	1	1	0	1	0	2
294	1	8	2	2	1	0	1	0	2
295	1	6	1	0	1	0	1	1	2
296	1	7	2	1	1	0	1	0	2
297	1	6	1	2	1	0	1	0	1
298	1	10	2	1	1	0	2	1	3
299	1	10	1	2	1	0	1	3	2
301	0	4	0	1	0	0	2	0	1
302	1	10	2	2	1	0	1	2	2
303	1	9	2	2	2	0	1	0	2
304	1	8	1	2	1	0	2	0	2
305	1	13	2	3	3	0	2	2	1
306	1	15	2	3	2	0	2	3	3
307	1	9	1	3	2	0	1	0	2
308	1	6	1	1	1	1	1	0	1
311	1	9	1	1	2	0	2	0	3
312	0	5	2	0	1	0	0	0	2
313	1	11	3	3	1	0	1	0	3
314	0	5	1	1	0	0	1	0	2
315	1	6	1	1	1	0	2	0	1
316	1	10	2	2	2	0	2	0	2
317	1	6	1	2	0	1	1	0	1
319	0	4	1	0	1	0	1	0	1
321	1	13	3	2	3	0	2	0	3
323	1	8	2	2	0	0	2	0	2
327	1	7	1	2	1	0	1	0	2
331	1	7	1	2	1	0	1	0	2
332	1	7	2	1	2	0	1	0	1
333	1	11	2	3	1	1	2	1	1
334	1	9	2	2	1	0	2	0	2
335	1	11	2	2	1	0	2	1	3
336	1	7	2	1	1	0	1	0	2
337	0	5	0	1	0	1	1	0	2
338	1	13	3	3	1	0	2	1	3
339	0	2	0	0	0	0	0	0	2
342	1	11	2	2	1	2	1	0	3
345	1	6	1	1	0	0	1	0	3

346	1	10	2	1	3	0	1	0	3
347	1	9	2	2	1	0	1	0	3
350	1	12	1	2	2	1	1	3	2
351	0	4	1	1	0	0	1	0	1
354	1	6	1	2	1	0	1	0	1
358	1	9	2	3	1	0	1	0	2
359	1	7	1	2	1	1	1	0	1
360	1	10	2	2	2	1	2	0	1
361	1	9	2	1	1	1	1	0	3
362	1	7	2	1	1	0	2	0	1
363	1	8	3	1	2	0	1	0	1
364	0	5	1	1	1	1	1	0	0
365	1	10	2	2	2	0	2	0	2
366	0	4	1	0	1	0	1	0	1
367	1	13	2	3	2	0	3	0	3
370	1	7	2	2	1	0	1	0	1
371	1	7	2	1	0	0	2	0	2
372	1	6	1	1	1	0	1	0	2
373	1	9	2	0	2	1	2	0	2
374	1	9	2	2	1	0	2	1	1
376	1	6	0	2	0	1	2	0	1
377	0	4	1	0	1	0	1	0	1
378	1	9	2	2	1	0	1	1	2
380	1	12	2	2	1	1	2	2	2
381	1	7	1	2	1	0	1	0	2
382	1	6	1	1	2	0	1	0	1
383	0	4	1	0	1	0	1	0	1
385	1	6	1	1	1	0	1	0	2
386	1	11	3	1	2	0	2	0	3
387	0	3	1	0	0	0	1	0	1
388	1	10	2	3	1	0	2	0	2
389	1	9	2	2	2	0	1	0	2
390	1	15	3	3	0	0	3	3	3
392	1	11	2	2	3	0	2	1	1
393	1	10	1	3	1	0	2	0	3
394	1	12	0	3	1	1	1	3	3
395	1	9	2	1	3	0	1	0	2
396	0	5	1	1	1	0	1	0	1
398	1	7	2	0	2	0	1	0	2
399	1	9	2	1	0	0	3	0	3
400	0	5	1	1	1	0	1	0	1
401	1	9	2	1	1	0	2	0	3
402	1	13	2	2	0	0	3	3	3
403	0	2	0	0	0	0	0	0	2
404	0	4	1	0	0	0	1	0	2
405	1	9	2	2	0	0	2	0	3
406	1	7	0	1	0	0	1	3	2
408	0	4	1	1	0	0	1	0	1
409	1	11	3	1	1	1	2	0	3
411	1	9	2	2	2	1	1	0	1
415	0	5	1	0	2	0	1	0	1
417	0	4	1	0	1	0	1	0	1
418	1	9	1	1	3	1	1	0	2
419	1	6	1	0	2	0	1	0	2
421	1	7	1	1	1	0	2	0	2
422	0	5	1	1	1	0	1	0	1
423	1	6	1	3	0	0	1	0	1
424	1	10	2	1	2	0	1	1	3
425	1	7	1	2	1	1	1	0	1
426	1	9	1	1	2	1	1	0	3
428	1	9	2	1	3	0	2	0	1
429	0	4	1	0	1	0	1	0	1
431	1	8	1	2	1	0	1	1	2
433	1	12	2	1	3	0	1	2	3
434	1	7	2	2	1	0	1	0	1
436	1	9	2	2	1	1	2	0	1

438	0	5	1	1	0	0	1	0	2
439	1	12	2	2	2	1	2	1	2
440	1	12	2	2	2	3	2	0	1
441	1	10	2	2	1	0	2	0	3
442	0	5	1	1	1	0	1	0	1
443	0	5	1	0	1	0	2	0	1
444	1	7	1	0	2	0	1	0	3
445	1	8	2	3	1	0	1	0	1
446	0	5	1	0	1	0	1	0	2
448	0	5	1	1	1	0	1	0	1
450	1	9	2	2	1	0	3	0	1
452	1	9	2	1	1	0	1	1	3
453	1	11	2	3	1	1	2	0	2
454	1	15	2	3	1	1	3	2	3
456	1	7	2	0	1	0	1	0	3
457	1	9	2	1	2	0	1	1	2
458	1	11	1	0	1	1	2	3	3
460	1	9	2	1	3	0	1	0	2
461	1	11	2	2	1	1	2	0	3
464	1	6	1	1	1	1	1	0	1
465	1	7	1	1	1	1	2	0	1
466	1	8	2	2	1	0	1	0	2
467	1	11	2	1	1	0	1	3	3
469	1	7	2	1	1	0	1	0	2
470	1	6	1	2	1	0	1	1	0
471	1	10	2	2	1	0	2	0	3
472	1	13	2	3	1	0	2	2	3
473	1	11	2	2	3	0	1	0	3
475	0	5	1	1	1	0	1	0	1
478	1	9	3	1	1	0	1	0	3
479	0	4	1	0	1	0	1	0	1
481	1	6	2	0	1	0	1	0	2
482	1	11	2	2	1	0	1	3	2
484	1	6	1	1	1	0	1	0	2
485	1	6	1	1	1	1	1	0	1
486	1	14	3	2	2	2	3	0	2
487	1	8	2	1	1	0	2	0	2
489	0	5	1	1	0	0	1	0	2
490	1	17	3	3	3	1	2	2	3
491	1	10	2	1	2	0	2	0	3
492	1	16	2	3	2	1	2	3	3
493	1	8	1	2	1	0	2	0	2
494	0	4	1	1	0	0	2	0	0
495	1	10	2	2	1	0	2	0	3
496	0	5	1	1	1	0	1	0	1
497	0	3	1	0	0	0	1	0	1
498	1	10	3	2	1	0	2	0	2
499	1	16	3	3	3	2	2	0	3
500	1	9	1	3	1	0	2	0	2
501	1	11	3	2	1	0	2	0	3
502	1	9	2	2	1	0	2	0	2
504	0	5	2	0	1	0	1	0	1
505	0	4	1	0	1	0	1	0	1
506	0	4	1	1	0	0	1	0	1
508	1	8	2	2	1	0	1	0	2
509	1	11	2	2	2	2	1	0	2
510	1	15	3	3	2	1	1	3	2
511	1	9	2	2	1	0	1	0	3
512	1	12	2	3	2	2	2	0	1
513	1	7	1	0	1	0	2	0	3
515	1	7	1	1	1	0	1	0	3
516	1	6	2	0	1	0	1	0	2
517	1	6	1	0	3	1	0	0	1
518	0	3	1	1	0	0	1	0	0
519	1	7	2	2	1	0	1	0	1
522	0	5	1	1	0	0	1	0	2

524	0	4	1	1	1	0	1	0	0
525	1	15	3	2	2	0	2	3	3
526	1	10	2	3	2	0	1	0	2
527	1	6	1	1	0	1	1	0	2
528	1	12	2	3	1	1	2	0	3
529	1	10	3	2	2	0	1	0	2
530	1	10	2	2	1	0	2	1	2
531	0	3	0	0	1	0	1	0	1
532	0	3	1	0	1	0	0	0	1
536	1	9	1	3	1	2	1	0	1
537	1	10	2	2	2	1	1	0	2
538	1	7	1	2	1	0	1	0	2
540	1	10	2	1	1	0	3	0	3
541	0	4	1	1	1	0	1	0	0
543	1	10	2	3	1	0	1	0	3
545	1	12	3	3	2	0	2	0	2
547	1	7	1	0	3	0	1	0	2
548	1	6	2	1	1	0	1	0	1
549	1	9	2	1	1	1	2	0	2
551	0	5	1	0	2	0	1	0	1
553	0	4	1	1	1	0	1	0	0
554	0	4	1	0	0	0	2	0	1
555	1	11	2	2	1	0	2	1	3
557	0	3	0	1	0	0	1	0	1
558	1	12	1	2	1	0	2	3	3
560	1	6	1	2	1	0	1	0	1
561	0	5	1	1	1	0	1	0	1
562	1	11	2	3	2	0	2	0	2
563	1	7	2	2	1	1	0	0	1
564	1	9	2	1	1	0	1	2	2
565	0	4	1	1	0	0	1	0	1
567	1	8	1	1	1	0	2	0	3
568	1	7	1	1	2	1	2	0	0
569	1	6	1	1	1	0	1	0	2
571	1	10	1	2	0	0	1	3	3
573	1	10	3	2	1	0	2	0	2
576	1	6	1	1	1	0	2	0	1
578	0	5	1	1	1	0	1	0	1
579	0	5	0	1	0	0	1	0	3
580	1	7	1	2	1	0	1	1	1
581	1	7	2	1	0	0	2	0	2
582	1	6	1	1	1	0	1	0	2
583	1	7	1	1	1	0	2	0	2
586	1	7	2	1	0	0	2	0	2
589	1	9	2	3	0	0	2	0	2
590	1	11	3	0	3	0	2	0	3
591	0	2	1	0	1	0	0	0	0
592	1	10	2	1	3	0	1	0	3
593	0	5	1	1	1	0	1	0	1
594	0	5	1	2	0	0	1	0	1
595	1	12	2	3	2	0	1	1	3
597	1	8	2	2	2	0	1	0	1
598	1	7	3	2	0	0	1	0	1
599	0	5	1	0	1	0	1	0	2
600	1	10	2	2	1	0	2	0	3
602	1	12	3	2	1	0	2	1	3
603	1	12	2	3	2	0	2	0	3
604	1	7	1	1	1	0	1	0	3
605	1	8	1	2	1	0	1	1	2
608	1	7	1	1	1	1	1	0	2
609	0	5	1	1	1	0	1	0	1
610	1	10	2	3	1	0	2	0	2
611	0	5	1	1	1	0	1	0	1
612	0	4	1	0	1	0	0	0	2
613	0	5	1	1	1	0	1	0	1
614	1	11	2	2	0	0	2	3	2

615	1	11	2	1	1	3	1	0	3
616	0	5	1	1	0	0	1	0	2
617	0	5	1	0	1	0	2	0	1
618	1	11	2	3	2	0	2	0	2
619	1	10	2	2	2	1	1	0	2
620	1	9	2	2	1	0	2	0	2
622	0	2	1	0	1	0	0	0	0
623	1	9	1	3	2	2	1	0	0
624	0	5	1	1	0	0	1	0	2
625	1	14	2	3	1	0	2	3	3
626	1	9	2	3	1	0	2	0	1
627	1	10	2	3	2	0	1	0	2
628	1	8	2	2	1	1	1	0	1
629	1	7	1	2	1	0	2	0	1
630	1	6	1	0	1	0	1	0	3
631	1	8	2	3	1	0	1	0	1
632	1	10	2	3	0	0	1	3	1
633	1	8	2	3	0	0	1	0	2
635	1	8	1	3	1	0	2	0	1
636	1	9	2	2	1	0	1	0	3
639	1	7	1	1	1	0	2	0	2
640	1	11	2	1	3	0	2	0	3
641	0	5	2	0	1	0	0	0	2
642	1	8	1	1	1	0	2	0	3
643	1	11	2	1	1	0	1	3	3
644	0	3	0	0	1	0	0	0	2
645	1	6	1	1	1	0	1	0	2
646	1	11	1	3	2	2	1	0	2
647	0	5	1	1	1	0	1	0	1
648	1	8	2	1	1	1	1	0	2
649	0	5	1	1	0	0	1	0	2
650	1	10	2	2	1	0	2	1	2
652	1	6	1	1	1	0	1	0	2
653	1	8	2	1	2	0	1	0	2
654	0	5	1	1	1	0	1	0	1
655	1	11	2	2	3	0	1	0	3
657	1	14	3	3	1	1	2	1	3
658	1	7	1	0	2	0	2	0	2
659	0	5	1	1	0	1	1	0	1
660	1	15	2	2	2	1	3	2	3
662	1	9	2	1	1	0	2	0	3
663	1	8	1	2	2	0	1	0	2
664	1	7	1	1	1	0	1	0	3
665	0	5	1	1	1	0	1	0	1
666	0	4	1	0	1	0	1	0	1
667	0	4	1	0	1	0	1	0	1
668	1	12	2	2	3	1	1	0	3
669	1	10	2	2	1	0	2	0	3
670	0	4	1	0	1	0	1	0	1
671	1	16	2	3	1	1	3	3	3
672	0	5	1	0	1	0	1	0	2
673	0	4	1	1	0	0	1	0	1
674	1	7	1	2	0	0	2	0	2
675	1	10	3	1	2	0	2	0	2
676	1	7	1	2	1	0	1	0	2
677	0	5	1	1	1	0	1	0	1
678	1	10	2	2	1	1	2	0	2
679	1	14	3	2	2	0	2	3	2
681	1	11	2	1	1	0	2	2	3
682	1	6	1	2	0	0	1	0	2
683	1	7	1	1	1	0	1	0	3
684	1	8	2	2	1	0	1	0	2
685	0	5	1	1	1	0	1	0	1
686	1	6	1	3	0	0	1	0	1
687	1	6	1	0	1	0	1	0	3
688	1	14	3	3	1	0	2	3	2

689	1	11	3	2	2	0	2	0	2
692	1	10	2	2	2	0	2	1	1
693	1	6	1	2	0	0	2	0	1
694	1	9	2	2	0	0	2	2	1
696	0	4	0	0	1	0	1	0	2
697	1	9	2	2	1	0	2	0	2
698	1	6	1	1	1	0	1	0	2
699	1	8	1	3	1	0	1	0	2
700	1	7	2	1	1	0	1	0	2
701	1	11	2	3	2	0	1	0	3
703	0	1	0	0	0	0	1	0	0
704	1	7	2	0	2	0	1	0	2
705	1	10	3	2	0	0	3	0	2
706	1	10	1	3	1	0	1	2	2
708	1	6	2	1	2	0	0	0	1
709	1	9	2	3	0	1	2	0	1
710	0	5	1	1	0	0	2	0	1
711	1	6	2	1	1	0	1	0	1
712	0	5	1	1	1	1	0	0	1
714	1	6	2	1	0	0	1	0	2
715	1	10	1	2	1	1	1	2	2
716	0	4	1	1	1	0	0	0	1
717	1	8	2	2	1	0	2	0	1
718	1	11	1	3	2	1	1	0	3
719	1	10	3	2	1	1	1	0	2
721	1	13	2	2	2	3	2	0	2
722	1	11	2	2	1	1	2	0	3
723	1	8	1	1	1	1	1	0	3
726	1	8	2	2	1	0	2	0	1
727	1	12	2	3	2	1	2	1	1
728	1	6	1	1	1	0	1	0	2
730	1	11	2	1	1	1	1	3	2
733	0	5	1	0	1	0	1	0	2
735	1	6	1	1	1	0	1	0	2
736	1	6	2	1	1	0	1	0	1
738	1	8	2	2	1	0	1	0	2
739	1	15	3	2	2	0	2	3	3
740	1	6	0	2	0	0	1	0	3
741	1	13	2	3	2	2	1	0	3
743	1	7	2	1	1	0	1	0	2
744	1	14	2	3	3	2	2	0	2
746	0	1	0	0	1	0	0	0	0
747	1	7	1	0	1	1	2	0	2
749	1	8	1	1	1	0	2	0	3
750	0	1	0	0	0	0	1	0	0
752	1	9	1	2	1	2	1	0	2
753	0	4	1	1	0	0	1	0	1
754	1	6	2	1	1	0	1	0	1
755	0	3	1	0	0	0	1	0	1
757	0	4	1	1	0	0	1	0	1
758	0	5	1	1	1	0	1	0	1
759	1	6	2	1	0	0	1	0	2
760	1	7	1	2	1	0	1	0	2
764	1	7	1	0	2	0	1	0	3
765	1	11	1	2	2	1	2	1	2
766	1	9	1	2	1	2	1	0	2
767	1	6	1	1	1	0	1	0	2
768	0	5	1	1	1	0	1	0	1
769	1	12	2	2	0	0	2	3	3
770	0	4	1	0	1	1	1	0	0
771	0	5	1	1	1	0	0	0	2
772	0	4	1	0	1	0	1	0	1
773	1	8	2	1	1	0	1	0	3
774	0	5	1	0	1	0	1	0	2
775	1	7	1	1	1	1	1	0	2
776	1	12	1	3	3	0	2	0	3

777	1	8	1	2	1	1	2	0	1
778	0	5	1	1	0	0	1	0	2
779	1	6	1	2	0	0	1	0	2
780	0	2	1	0	0	0	0	0	1
781	1	7	2	1	0	0	1	0	3
783	1	6	1	1	1	0	1	0	2
784	1	6	1	1	1	0	1	0	2
785	1	7	2	1	2	0	1	0	1
786	1	8	1	2	1	0	1	1	2
787	1	13	2	2	2	3	2	0	2
790	1	8	2	1	0	0	2	0	3
791	1	12	2	2	2	3	1	0	2
792	1	7	1	2	1	0	1	0	2
793	0	3	0	0	0	0	1	0	2
797	1	7	1	1	1	0	2	0	2
798	1	10	2	3	0	1	2	0	2
800	0	5	1	1	1	0	1	0	1
801	0	3	1	1	0	0	1	0	0
803	0	5	1	1	0	0	1	1	1
804	1	8	1	2	1	0	1	0	3
805	1	9	2	3	1	0	1	0	2
806	1	7	2	2	1	0	1	0	1
808	1	6	1	0	0	0	1	1	3
809	1	10	2	2	2	0	1	0	3
810	1	10	2	1	2	1	2	0	2
811	0	5	1	0	1	0	1	0	2
813	1	11	2	2	2	1	1	0	3
814	0	2	1	0	0	0	1	0	0
815	1	8	2	2	1	0	1	0	2
817	1	6	1	1	1	1	0	0	2
818	1	12	2	2	3	0	2	1	2
820	0	2	0	0	0	0	1	0	1
821	1	6	1	1	1	1	1	0	1
824	1	7	2	1	1	0	1	0	2
825	1	8	2	2	1	0	1	0	2
826	0	2	0	0	0	0	1	0	1
827	1	8	2	3	1	0	1	0	1
829	1	7	2	1	1	0	2	0	1
830	1	9	2	2	1	0	2	0	2
831	1	7	1	1	1	0	2	0	2
833	1	6	1	2	0	0	1	0	2
834	1	9	3	2	1	0	1	1	1
835	0	3	0	1	0	0	1	0	1
837	1	9	2	2	1	0	2	0	2
838	0	5	1	2	1	0	0	0	1
839	1	10	1	3	0	0	1	3	2
840	1	11	2	1	2	1	2	0	3
841	0	4	1	0	1	0	1	0	1
842	1	11	3	3	0	1	2	0	2
844	0	5	1	1	1	0	1	0	1
845	1	7	2	2	0	0	1	0	2
846	1	11	1	2	1	1	2	1	3
847	1	8	2	2	1	0	1	0	2
848	1	7	1	1	1	0	2	0	2
850	0	5	1	0	1	0	1	0	2
851	1	6	1	1	1	0	1	0	2
852	1	8	1	3	0	0	1	0	3
853	1	8	2	1	2	0	1	0	2
854	1	6	1	2	1	0	1	0	1
856	1	11	2	2	3	0	2	1	1
857	1	10	2	0	0	0	2	3	3
858	1	6	1	0	2	0	1	0	2
861	0	5	1	0	1	0	1	0	2
862	1	6	2	1	1	0	1	0	1
863	1	13	2	3	1	1	3	0	3
864	0	4	1	1	0	0	1	0	1

865	1	9	2	1	1	0	1	1	3
866	1	8	2	2	1	0	1	0	2
867	1	15	3	3	3	2	2	0	2
868	1	9	2	1	2	0	1	0	3
869	0	3	1	0	0	0	1	0	1
870	1	13	3	3	0	0	2	2	3
872	1	13	2	3	2	0	3	0	3
873	0	4	0	0	1	0	1	0	2
874	1	8	1	3	2	0	1	0	1
875	0	4	1	1	1	0	0	0	1
876	1	6	1	2	0	0	1	0	2
877	0	5	1	1	0	0	2	0	1
878	1	9	2	2	1	0	1	0	3
879	0	4	1	1	0	0	1	0	1
880	1	7	2	1	1	0	1	0	2
881	1	10	2	1	2	0	2	0	3
882	0	5	1	0	1	0	1	0	2
884	1	10	2	1	2	0	2	0	3
885	1	11	3	1	2	0	2	0	3
886	1	6	1	0	1	0	1	0	3
887	1	6	1	1	1	0	1	0	2
889	1	7	1	0	3	0	1	0	2
890	1	9	1	2	1	0	2	0	3
891	1	14	2	2	1	1	2	3	3
892	1	6	1	2	1	0	1	0	1
894	1	8	2	2	1	0	2	0	1
895	0	4	0	1	1	0	1	0	1
896	1	8	1	1	2	0	2	0	2
898	1	9	2	2	1	0	2	0	2
900	0	0	0	0	0	0	0	0	0
902	1	8	2	2	2	0	1	0	1
903	1	8	1	2	1	0	2	1	1
904	1	13	2	2	1	0	2	3	3
905	1	8	2	1	1	0	1	0	3
906	1	8	1	1	2	0	1	0	3
907	0	5	2	0	1	0	1	0	1
908	0	3	1	0	1	0	0	0	1
909	1	15	2	3	1	2	1	3	3
910	1	6	1	0	1	1	0	0	3
912	1	9	2	3	1	0	1	0	2
914	1	6	1	0	1	0	2	0	2
915	1	13	3	2	3	0	2	0	3
916	1	6	1	0	2	0	1	0	2
919	1	9	2	3	1	0	1	0	2
920	1	7	3	1	1	0	0	0	2
921	1	6	1	0	3	0	1	0	1
922	1	8	2	1	2	0	2	0	1
923	0	4	0	1	0	0	1	0	2
925	1	6	1	1	1	0	1	0	2
926	1	10	1	2	1	1	2	0	3
927	1	6	1	2	1	0	1	0	1
928	1	13	2	2	1	3	2	1	2
930	1	6	1	0	1	0	1	0	3
931	1	9	2	2	1	0	1	1	2
932	0	2	0	0	0	0	1	0	1
934	1	11	2	3	1	0	3	0	2
935	1	9	2	3	2	0	1	0	1
936	1	6	1	2	0	0	2	0	1
938	0	4	0	1	0	1	1	0	1
939	1	9	1	3	1	0	1	0	3
940	1	8	2	2	1	0	1	0	2
944	1	8	1	1	0	0	1	3	2
945	0	4	0	1	0	0	1	0	2
946	1	9	2	2	2	0	1	0	2
947	1	8	2	1	1	0	2	0	2
948	1	6	1	2	0	0	2	0	1

949	1	6	1	2	0	0	1	0	2
950	1	10	3	2	1	0	2	0	2
951	0	5	1	1	1	0	1	0	1
952	1	6	1	1	1	0	1	1	1
953	1	9	1	1	1	1	2	0	3
954	0	5	1	1	1	0	1	0	1
955	1	9	1	2	2	2	1	0	1
957	1	7	1	1	1	1	1	0	2
959	1	14	2	2	2	0	2	3	3
960	1	8	2	2	1	0	1	0	2
961	1	8	1	2	1	0	1	0	3
964	1	7	1	1	1	0	2	1	1
965	0	5	1	1	1	0	1	0	1
966	1	13	2	3	2	1	2	0	3
967	1	9	3	0	2	0	1	0	3
968	1	9	3	2	0	0	2	0	2
969	1	9	2	2	1	0	2	1	1
970	0	3	0	1	0	0	1	0	1
971	1	6	1	0	1	0	1	1	2
974	1	6	1	2	1	0	1	0	1
976	0	3	1	1	0	0	1	0	0
977	1	10	2	3	1	1	1	0	2
978	1	11	2	1	1	0	2	3	2
981	0	5	1	2	0	0	1	0	1
983	0	5	0	0	2	0	1	0	2
984	1	7	0	1	2	1	1	0	2
985	0	4	1	0	1	0	1	0	1
986	1	10	2	2	2	0	1	0	3
987	1	6	1	0	1	1	2	0	1
992	1	8	2	2	1	0	1	0	2
995	1	9	2	1	2	1	1	0	2
997	0	5	1	1	1	0	1	0	1
998	1	6	1	1	1	0	2	0	1
999	1	7	1	2	1	0	1	0	2
1000	0	5	1	1	1	0	1	0	1
1001	1	6	1	1	1	0	1	0	2
1002	1	10	3	1	1	0	2	1	2
1003	1	9	2	2	1	0	2	0	2
1004	0	5	1	1	1	0	1	0	1
1005	1	6	1	1	1	0	1	0	2
1006	1	11	2	2	2	1	2	0	2
1007	1	6	2	0	1	0	1	0	2
1008	0	3	0	0	1	0	1	0	1
1009	0	5	1	2	0	0	1	0	1
1011	0	5	0	2	1	1	1	0	0
1012	1	9	1	2	1	0	1	2	2
1013	1	7	2	1	1	0	1	0	2
1014	0	4	1	1	1	0	1	0	0
1015	1	9	2	1	3	0	1	0	2
1016	0	4	1	0	0	0	1	0	2
1017	1	15	3	3	2	3	2	0	2
1019	1	6	2	0	1	0	1	0	2
1020	0	4	1	1	0	0	1	0	1
1021	0	4	1	0	0	0	2	0	1
1022	0	3	0	1	0	0	1	0	1
1023	0	4	1	0	1	0	1	0	1
1024	0	5	2	0	1	0	1	0	1
1026	0	5	1	1	1	0	1	0	1
1027	1	8	2	3	0	0	1	0	2
1028	1	6	1	2	0	1	1	0	1
1029	1	13	2	2	3	3	1	0	2
1030	0	2	0	0	1	0	1	0	0
1031	1	12	3	3	1	2	2	0	1
1032	0	5	1	1	1	0	1	0	1
1033	1	6	1	0	2	0	2	0	1
1034	1	6	0	1	1	1	2	0	1

1036	0	4	1	1	0	0	1	0	1
1037	1	9	2	1	2	0	1	0	3
1038	1	10	2	1	1	1	2	0	3
1039	0	5	1	1	0	0	2	0	1
1040	0	5	1	0	2	0	1	0	1
1041	1	6	1	1	1	0	1	0	2
1042	1	15	3	3	3	0	1	3	2
1043	0	5	1	2	1	1	0	0	0
1045	1	11	2	2	1	0	2	1	3
1046	1	6	1	1	0	0	3	0	1
1048	1	14	3	3	2	0	3	0	3
1049	0	5	1	1	1	0	1	0	1
1051	1	6	1	1	1	0	1	0	2
1052	1	8	2	1	1	0	1	0	3
1053	1	11	2	2	1	0	2	2	2
1054	1	9	2	1	2	0	1	0	3
1055	1	9	1	2	0	0	2	1	3
1056	1	10	3	2	1	0	1	0	3
1061	1	8	1	3	1	0	1	0	2
1062	1	7	1	1	1	0	1	0	3
1064	1	10	2	2	1	1	1	0	3
1065	0	5	1	0	1	0	1	0	2
1067	1	8	2	2	1	1	1	1	0
1068	1	6	1	1	1	0	1	0	2
1069	0	5	1	0	1	0	1	0	2
1070	1	10	2	1	0	0	2	3	2
1071	1	10	2	2	1	0	1	2	2
1072	1	8	1	2	1	0	2	0	2
1073	1	7	1	2	1	0	1	0	2
1074	0	5	1	0	1	0	1	0	2
1077	1	7	1	1	0	1	2	0	2
1078	1	6	1	1	1	0	1	0	2
1080	1	8	1	1	1	0	1	3	1
1081	0	4	1	0	0	0	1	0	2
1082	1	6	1	1	1	0	1	0	2
1083	1	6	2	1	0	0	2	0	1
1086	1	13	3	3	2	0	2	0	3
1087	1	6	1	1	1	0	1	0	2
1088	0	5	1	0	0	0	1	0	3
1089	1	7	2	2	1	0	1	0	1
1092	0	3	0	1	0	0	1	0	1
1093	1	8	1	1	1	0	2	0	3
1094	0	2	0	0	0	0	1	0	1
1095	1	7	1	1	1	0	2	0	2
1097	1	9	1	1	2	0	1	3	1
1098	0	4	0	1	0	0	1	1	1
1099	1	9	1	2	1	1	1	0	3
1100	1	6	1	1	1	0	1	0	2
1102	1	19	3	3	3	3	2	3	2
1103	0	5	1	1	1	1	0	0	1
1104	0	3	1	0	0	0	1	0	1
1105	1	10	2	0	2	0	1	2	3
1107	1	7	2	0	2	0	1	0	2
1108	1	10	2	3	1	0	1	0	3
1109	1	7	1	1	1	0	2	0	2
1110	1	8	2	1	1	1	1	0	2
1111	0	2	0	0	0	0	1	0	1
1112	0	5	1	1	1	0	1	0	1
1113	1	12	3	2	2	0	2	0	3
1114	1	15	2	3	2	1	2	2	3
1115	0	4	1	2	0	0	1	0	0
1116	1	8	1	1	2	0	1	0	3
1118	1	9	3	1	1	0	1	0	3
1120	1	11	2	3	0	0	1	2	3
1122	1	14	3	3	1	0	2	2	3
1123	1	17	3	3	3	3	2	0	3

1124	1	13	2	3	1	2	2	0	3
1125	1	7	1	2	1	0	2	0	1
1126	1	8	1	2	2	0	2	0	1
1128	1	14	2	3	3	0	3	0	3
1132	1	11	3	1	1	0	1	2	3
1133	1	9	3	2	1	0	2	0	1
1134	1	9	1	2	0	0	3	0	3
1135	1	7	2	1	1	0	1	0	2
1136	1	8	2	3	0	1	1	0	1
1138	1	11	2	2	2	0	2	0	3
1139	1	8	1	3	1	0	1	0	2
1141	0	5	1	1	0	0	1	0	2
1142	1	12	2	2	3	0	2	0	3
1143	1	10	2	3	1	0	2	0	2
1144	1	14	3	3	3	0	2	0	3
1145	0	5	1	1	0	1	1	0	1
1146	1	10	2	2	1	0	1	1	3
1147	1	10	2	3	1	0	2	0	2
1149	1	16	3	3	2	0	2	3	3
1150	1	9	1	2	1	0	2	1	2
1151	1	8	1	2	1	0	2	1	1
1152	0	4	1	0	1	0	1	0	1
1153	1	12	2	3	2	0	2	0	3
1154	0	5	2	0	1	0	1	0	1
1155	1	6	0	2	1	0	1	0	2
1156	1	8	2	1	1	0	2	0	2
1157	1	8	2	1	1	0	1	0	3
1158	0	5	1	1	1	0	1	0	1
1159	1	15	2	3	3	2	1	2	2
1161	1	8	1	1	1	0	2	0	3
1162	1	12	2	3	2	2	2	0	1
1165	1	12	2	2	3	0	1	1	3
1166	1	9	1	1	2	2	1	0	2
1169	1	10	2	2	1	0	2	0	3
1170	0	3	1	1	0	0	1	0	0
1171	1	9	2	2	1	0	1	0	3
1173	0	5	1	1	1	0	1	0	1
1174	1	7	1	0	1	0	2	0	3
1175	1	6	2	0	1	0	2	0	1
1177	1	12	2	3	0	1	1	3	2
1178	1	8	2	2	1	0	1	0	2
1179	0	5	0	1	2	1	1	0	0
1180	1	10	2	3	2	0	1	0	2
1181	1	9	2	2	1	0	2	1	1
1182	1	6	2	1	0	0	1	0	2
1183	1	14	2	2	1	0	3	3	3
1186	1	9	1	1	2	0	2	0	3
1187	1	12	3	3	1	1	2	0	2
1188	0	4	1	1	0	0	1	0	1
1189	1	12	2	3	2	0	1	1	3
1191	0	4	1	0	0	0	1	0	2
1193	1	9	2	3	1	0	1	0	2
1194	1	7	1	1	0	0	1	2	2
1195	1	9	2	3	1	0	1	0	2
1196	0	4	1	1	0	0	1	0	1
1197	1	10	1	2	1	0	1	3	2
1198	0	4	0	0	0	0	1	3	0
1199	1	9	2	2	2	0	1	0	2
1200	1	8	2	2	1	0	2	0	1
1201	1	8	1	1	1	0	2	0	3
1202	0	4	0	1	0	0	1	0	2
1203	1	12	3	3	3	0	1	0	2
1205	1	10	3	3	0	0	2	0	2
1206	1	6	2	0	1	0	2	0	1
1207	1	10	1	2	1	0	2	2	2
1208	1	7	1	2	1	0	2	0	1

1209	1	8	2	1	2	0	1	0	2
1210	1	8	2	1	0	0	2	0	3
1211	0	5	1	1	0	1	1	0	1
1212	1	11	3	1	1	0	2	1	3
1214	0	4	0	1	0	0	1	0	2
1215	0	5	0	1	1	0	1	0	2
1216	1	8	2	1	1	0	2	0	2
1217	0	4	1	1	1	0	1	0	0
1218	1	15	2	3	3	2	2	0	3
1220	1	9	2	3	0	0	2	0	2
1221	1	10	2	3	0	0	2	1	2
1222	1	13	2	3	2	0	2	1	3
1223	1	7	2	1	1	0	1	0	2
1225	0	4	1	0	1	0	1	0	1
1226	1	8	2	3	0	0	2	0	1
1227	1	6	1	1	1	0	1	0	2
1228	1	6	1	1	1	0	1	0	2
1229	1	13	3	3	0	1	3	0	3
1230	1	16	3	3	3	0	1	3	3
1231	1	13	3	3	1	0	3	0	3
1232	1	7	1	2	0	1	1	0	2
1233	1	7	1	3	0	0	1	0	2
1235	1	9	2	3	2	0	0	0	2
1236	0	5	1	0	1	1	1	0	1
1237	1	15	2	3	3	2	3	0	2
1239	1	10	2	1	3	0	1	0	3
1241	0	5	1	2	0	0	1	0	1
1242	0	1	1	0	0	0	0	0	0
1244	1	8	2	2	0	1	1	0	2
1245	1	9	2	1	3	0	1	0	2
1247	1	6	1	1	1	0	1	0	2
1248	0	5	1	1	1	0	0	0	2
1249	1	8	2	2	1	0	1	0	2
1250	1	17	3	2	3	3	2	2	2
1251	0	4	1	0	1	0	1	0	1
1252	1	8	1	3	0	0	1	0	3
1253	1	9	2	1	2	0	2	0	2
1254	1	10	2	0	2	0	2	1	3
1255	1	16	3	2	2	1	2	3	3
1256	1	11	2	3	1	1	2	1	1
1259	1	10	1	2	1	0	2	2	2
1262	0	4	1	1	0	0	1	0	1
1264	0	5	1	1	1	0	1	0	1
1265	1	10	2	2	1	0	2	1	2
1266	1	8	2	0	2	0	1	0	3
1268	1	6	1	1	1	0	1	0	2
1269	1	10	2	2	2	0	1	0	3
1270	1	6	2	1	1	0	1	0	1
1271	1	8	1	3	1	0	1	0	2
1272	1	7	1	1	1	1	1	0	2
1273	1	8	2	1	2	0	1	0	2
1275	1	10	2	3	1	2	1	0	1
1277	0	5	1	1	1	0	1	0	1
1278	1	7	3	0	1	0	0	0	3
1280	0	3	1	0	0	0	1	0	1
1281	1	10	2	1	1	0	2	1	3
1282	1	6	1	1	1	0	1	0	2
1283	1	10	1	2	1	2	1	0	3
1285	0	2	0	0	1	1	0	0	0
1287	1	11	2	2	2	0	2	0	3
1289	1	15	3	3	3	0	3	0	3
1290	1	17	3	3	3	3	2	0	3
1291	1	6	1	1	1	0	1	0	2
1292	1	9	2	1	3	0	1	1	1
1293	1	9	1	0	2	0	2	1	3
1294	1	8	2	1	1	0	2	0	2

1295	0	3	1	1	0	0	1	0	0
1296	1	6	2	0	0	0	1	0	3
1298	1	15	2	3	2	0	2	3	3
1299	0	4	1	0	1	0	1	0	1
1300	1	7	1	2	1	1	1	0	1
1302	0	5	1	1	1	0	1	0	1
1303	0	2	0	0	0	0	1	0	1
1304	1	8	2	2	1	0	2	0	1
1305	1	6	0	1	1	2	1	0	1
1308	1	7	1	0	0	0	1	3	2
1309	1	8	2	2	1	0	1	0	2
1314	0	4	0	1	1	0	1	0	1
1315	1	12	3	3	1	0	3	0	2
1317	1	6	1	1	1	0	2	0	1
1319	1	11	3	2	2	0	2	0	2
1320	1	15	3	3	1	2	3	1	2
1321	1	11	2	3	2	0	2	0	2
1322	1	9	2	0	2	1	1	0	3
1324	1	10	2	2	2	1	1	0	2
1325	1	11	2	1	2	2	1	0	3
1326	1	8	2	2	1	0	2	0	1
1327	1	6	1	1	1	0	1	0	2
1328	1	10	1	2	0	0	2	3	2
1330	1	6	2	1	1	0	1	0	1
1331	1	10	1	3	2	3	1	0	0
1333	1	8	1	2	2	1	0	0	2
1334	0	3	1	1	1	0	0	0	0
1335	1	8	1	1	1	1	2	0	2
1338	0	5	1	1	1	0	1	0	1
1339	1	10	2	3	2	0	1	0	2
1340	1	8	1	2	1	0	2	0	2
1341	1	6	1	2	0	0	1	0	2
1342	1	9	2	1	2	1	1	0	2
1345	1	7	1	2	1	0	1	0	2
1346	1	9	2	1	1	0	2	0	3
1347	1	14	3	3	3	3	1	0	1
1348	1	9	2	0	3	0	1	0	3
1349	1	13	2	3	1	2	2	0	3
1350	1	8	2	2	2	0	1	0	1
1351	0	5	1	1	0	0	1	0	2
1352	1	9	2	2	1	0	1	0	3
1354	1	8	2	0	2	0	1	0	3
1355	1	12	3	3	1	1	1	0	3
1356	1	7	1	1	1	1	1	0	2
1359	1	6	2	0	1	0	1	0	2
1361	1	7	2	0	1	0	1	0	3
1362	1	10	2	3	0	1	2	0	2
1363	1	10	1	1	0	0	2	3	3
1366	1	11	2	2	2	1	1	0	3
1367	1	6	2	1	1	0	1	0	1
1369	1	6	1	1	0	0	2	1	1
1370	1	17	3	3	3	3	2	0	3
1371	1	11	2	2	2	0	2	0	3
1372	1	8	2	1	2	0	1	0	2
1375	0	4	1	1	0	0	1	0	1
1376	1	9	1	2	1	0	2	1	2
1377	1	7	2	1	0	0	2	2	0
1378	1	7	1	1	1	0	1	0	3
1380	0	2	0	1	1	0	0	0	0
1381	0	5	1	1	1	0	1	0	1
1382	1	6	1	1	0	1	1	0	2
1383	1	9	2	0	1	1	2	0	3
1385	1	9	1	1	2	0	1	2	2
1387	1	9	2	2	1	0	2	0	2
1389	1	10	2	2	2	0	1	0	3
1390	1	8	2	0	1	1	1	0	3

1393	0	5	1	1	1	0	1	0	1
1396	1	8	2	1	0	0	2	0	3
1398	1	6	1	1	2	0	1	0	1
1399	1	8	2	1	1	0	1	0	3
1400	1	9	2	2	1	0	1	0	3
1401	0	4	2	1	1	0	0	0	0
1402	1	7	1	0	1	3	1	0	1
1404	1	9	2	3	1	0	1	0	2
1406	0	5	1	0	0	0	2	0	2
1407	0	5	1	0	0	1	2	0	1
1408	1	10	2	2	1	1	1	0	3
1409	1	9	1	3	1	0	1	0	3
1411	1	6	1	2	0	0	1	0	2
1412	1	7	1	1	2	0	1	0	2
1414	1	7	2	1	1	0	1	0	2
1416	1	6	1	1	1	0	2	0	1
1418	1	13	3	2	1	0	1	3	3
1419	1	8	2	1	1	0	2	0	2
1420	1	7	1	2	1	0	1	0	2
1421	1	10	2	2	2	0	1	2	1
1422	1	9	1	2	0	0	2	2	2
1423	0	1	0	0	0	0	0	0	1
1424	1	12	2	2	1	1	2	3	1
1427	1	10	2	1	3	0	1	1	2
1428	1	9	1	2	1	0	2	0	3
1429	1	9	1	2	2	1	1	0	2
1430	1	14	2	3	3	2	2	0	2
1431	1	8	2	2	1	0	1	0	2
1432	1	6	1	0	0	0	1	2	2
1433	1	12	2	2	2	1	2	0	3
1434	1	6	1	2	1	0	1	0	1
1436	1	9	2	3	1	0	1	0	2
1437	1	13	3	2	2	1	2	0	3
1438	0	5	1	0	1	0	1	0	2
1440	0	3	1	1	0	0	1	0	0
1441	1	11	2	2	2	1	1	1	2
1442	1	7	2	0	1	0	2	0	2
1445	0	2	0	1	0	0	1	0	0
1447	1	10	1	0	2	2	2	0	3
1448	1	7	1	1	0	0	2	0	3
1449	0	5	1	2	1	0	1	0	0
1452	1	9	2	2	1	0	2	0	2
1453	1	8	2	0	2	0	2	0	2
1456	1	10	1	2	1	0	1	3	2
1457	1	10	0	1	1	2	2	1	3
1458	1	14	2	3	1	0	2	3	3
1459	1	6	1	1	1	0	1	0	2
1460	0	5	1	0	1	0	1	0	2
1462	0	3	1	0	1	0	1	0	0
1465	0	4	1	0	1	0	1	0	1
1466	1	10	2	2	2	0	1	1	2
1467	1	10	2	2	1	0	2	0	3
1468	1	7	1	2	2	0	1	0	1
1469	1	10	1	3	2	1	1	0	2
1470	1	7	1	1	1	1	2	0	1
1471	1	9	1	3	1	0	1	0	3
1472	0	5	1	1	1	0	1	0	1
1474	0	4	1	1	0	0	1	0	1
1476	1	10	2	1	1	0	2	1	3
1477	0	3	1	0	0	0	1	0	1
1478	1	7	1	2	1	0	2	0	1
1479	1	11	2	3	1	0	2	1	2
1483	0	3	0	1	0	0	1	0	1
1484	1	8	2	1	1	1	1	0	2
1485	1	6	1	0	1	0	2	0	2
1486	1	7	1	1	1	0	1	0	3

1487	0	5	2	2	0	0	1	0	0
1488	1	7	1	2	0	0	1	0	3
1489	1	15	3	3	2	2	1	2	2
1491	1	6	1	1	1	0	1	1	1
1492	1	10	2	3	2	0	1	0	2
1493	1	6	1	1	2	0	1	0	1
1494	1	6	1	2	1	0	1	0	1
1495	0	5	1	1	1	0	1	0	1
1496	0	4	1	0	0	0	1	0	2
1497	1	8	2	1	1	0	2	0	2
1498	1	8	2	0	2	1	1	0	2
1499	1	13	2	3	1	0	2	2	3
1500	1	10	2	1	1	0	2	1	3
1502	0	5	0	1	1	1	1	0	1
1503	1	6	1	0	0	1	2	0	2
1505	1	8	1	1	1	0	1	2	2
1506	1	9	2	3	1	0	2	0	1
1507	1	11	3	3	1	0	1	1	2
1508	1	6	2	0	1	0	1	0	2
1509	1	6	1	1	0	0	1	0	3
1511	0	2	0	0	0	0	1	0	1
1512	0	1	0	0	0	0	1	0	0
1513	1	14	3	2	2	3	1	0	3
1514	1	10	3	2	2	0	1	1	1
1515	0	5	0	1	1	1	1	0	1
1516	1	6	1	2	1	0	1	0	1
1517	1	10	2	2	1	1	1	0	3
1518	1	8	2	0	2	1	1	0	2
1519	1	9	1	3	1	0	1	0	3
1520	0	4	0	1	0	0	1	0	2
1521	1	16	3	3	2	1	2	3	2
1523	1	18	2	3	3	3	1	3	3
1525	1	6	1	1	1	0	1	0	2
1526	0	4	0	1	0	0	1	0	2
1527	1	9	1	3	2	0	1	0	2
1528	1	8	2	0	2	0	2	0	2
1530	1	11	2	2	2	1	1	0	3
1532	1	6	1	1	1	0	1	0	2
1533	1	11	1	2	1	0	2	3	2
1535	1	9	2	2	1	0	1	0	3
1536	1	7	2	1	1	0	1	0	2
1537	1	8	2	2	1	0	1	1	1
1538	1	6	2	1	1	0	1	0	1
1539	1	11	2	3	1	1	1	1	2
1542	1	8	2	1	1	0	1	0	3
1544	1	8	1	2	1	0	1	0	3
1545	0	5	1	0	1	0	1	0	2
1549	1	8	1	2	1	1	1	0	2
1550	1	8	1	2	0	0	2	0	3
1552	1	8	1	1	1	1	2	0	2
1553	0	5	1	2	0	0	1	0	1
1554	1	8	2	0	1	1	2	0	2
1555	1	6	1	1	0	0	2	0	2
1556	0	5	1	1	1	0	1	0	1
1557	1	9	2	2	1	0	1	0	3
1559	0	4	1	1	0	0	1	0	1
1560	1	6	1	1	1	0	1	0	2
1561	1	7	1	3	1	0	1	0	1
1562	1	11	2	3	1	0	2	0	3
1564	1	14	2	3	1	2	3	0	3
1565	0	5	2	0	1	0	1	0	1
1566	1	9	3	1	1	0	1	0	3
1567	1	8	1	1	2	2	1	0	1
1568	1	7	2	1	0	0	2	0	2
1570	1	8	2	1	1	0	2	0	2
1571	1	7	2	1	0	0	1	0	3

1572	1	6	1	0	1	0	1	0	3
1575	1	6	1	1	1	0	1	0	2
1576	1	6	1	2	1	0	1	0	1
1578	1	12	2	3	1	1	3	0	2
1579	0	1	0	0	1	0	0	0	0
1580	0	3	1	1	0	0	1	0	0
1581	1	7	1	2	1	1	1	0	1
1582	1	7	1	1	2	1	1	0	1
1583	0	4	1	0	0	0	1	0	2
1584	0	3	0	0	0	0	1	0	2
1585	0	5	1	1	0	0	1	0	2
1586	1	10	2	3	2	0	1	0	2
1587	1	7	1	2	0	0	1	0	3
1589	1	6	1	1	1	0	1	0	2
1590	1	6	1	0	1	0	1	0	3
1591	1	10	2	2	1	0	2	0	3
1592	1	9	2	2	1	0	1	0	3
1593	1	11	3	2	2	0	1	0	3
1594	1	6	1	2	1	0	1	0	1
1595	1	10	2	1	2	0	2	0	3
1598	1	8	1	2	1	0	1	0	3
1599	1	8	1	2	1	0	2	0	2
1602	1	11	3	2	1	0	2	0	3
1603	1	11	2	1	2	1	2	0	3
1605	1	8	1	3	0	0	1	3	0
1606	1	7	1	1	2	0	1	0	2
1607	1	8	1	3	2	0	1	0	1
1608	1	6	1	2	1	0	1	0	1
1610	1	6	1	0	1	0	1	0	3
1611	1	7	1	3	0	0	2	0	1
1612	0	4	1	0	1	0	0	0	2
1613	1	9	3	1	1	0	2	0	2
1614	1	10	3	2	1	0	2	0	2
1616	1	8	1	3	2	0	0	0	2
1617	0	5	1	1	0	0	1	0	2
1618	1	10	2	2	1	0	2	0	3
1619	1	10	2	1	2	0	1	1	3
1621	0	5	1	1	0	0	1	0	2
1622	0	4	1	0	1	0	1	0	1
1624	0	5	1	1	1	0	1	0	1
1625	1	12	2	1	1	0	2	3	3
1626	0	5	1	1	1	0	1	0	1
1627	0	5	1	1	1	0	1	0	1
1628	0	4	1	2	0	0	1	0	0
1629	1	8	2	1	2	0	1	0	2
1632	1	6	1	1	1	0	2	0	1
1633	1	9	1	2	1	0	2	0	3
1634	1	8	3	0	2	0	0	0	3
1635	0	5	1	1	1	0	1	0	1
1636	1	8	1	3	0	1	1	0	2
1637	1	9	2	2	1	0	1	0	3
1638	0	5	1	0	1	0	1	0	2
1639	1	7	1	2	1	0	2	0	1
1640	1	6	0	1	0	0	1	3	1
1641	1	10	2	2	1	0	1	1	3
1643	1	7	2	1	1	0	1	0	2
1644	1	8	1	3	1	0	1	0	2
1645	1	7	1	1	1	0	2	0	2
1646	1	14	2	2	3	0	2	2	3
1649	0	3	0	0	0	0	1	0	2
1650	0	4	1	1	0	0	1	0	1
1652	1	8	1	2	1	1	1	0	2
1653	1	10	1	1	2	1	2	0	3
1654	0	5	1	1	1	0	1	0	1
1655	1	8	2	1	1	0	2	0	2
1656	1	10	1	3	1	0	2	0	3

1657	1	6	1	1	1	0	1	0	2
1658	0	1	0	0	0	0	1	0	0
1659	0	5	1	0	1	1	1	0	1
1662	1	6	1	1	1	0	1	0	2
1665	0	5	1	1	1	0	1	0	1
1667	1	6	1	1	1	0	2	0	1
1668	1	6	1	0	2	0	1	0	2
1669	0	5	1	1	1	0	1	0	1
1670	1	9	1	3	1	2	1	0	1
1671	1	7	1	1	1	0	1	0	3
1672	1	10	2	3	2	0	1	0	2
1674	1	10	1	2	1	0	1	3	2
1675	0	3	1	1	0	0	1	0	0
1676	0	4	1	1	0	0	1	0	1
1677	1	14	2	3	3	2	3	0	1
1680	1	10	1	2	2	0	1	3	1
1681	1	7	2	1	1	0	1	1	1
1682	1	7	1	1	0	0	1	1	3
1683	0	4	1	0	0	0	1	0	2
1684	1	6	1	1	1	0	1	0	2
1687	1	12	2	2	3	0	1	1	3
1688	1	7	0	0	2	0	3	0	2
1689	1	8	3	0	3	0	0	0	2
1690	1	7	1	3	1	0	1	0	1
1691	0	5	1	2	0	0	1	0	1
1693	1	8	3	0	2	0	1	0	2
1694	1	10	2	2	2	1	1	0	2
1695	1	9	1	3	0	0	1	3	1
1696	1	6	1	1	1	0	1	0	2
1697	0	3	1	0	0	0	1	0	1
1698	1	9	2	0	2	0	1	1	3
1700	1	7	1	1	1	0	1	2	1
1702	1	8	2	2	0	0	2	0	2
1703	1	6	1	1	1	1	1	0	1
1705	1	14	3	1	2	2	2	1	3
1706	1	7	1	1	1	0	1	0	3
1707	1	9	2	2	2	0	2	0	1
1708	1	11	2	2	1	0	2	2	2
1709	1	9	1	2	1	0	1	2	2
1710	0	5	1	1	1	0	1	0	1
1711	1	11	3	0	3	0	1	1	3
1712	1	6	1	2	0	1	1	0	1
1713	0	3	1	1	0	0	0	0	1
1714	1	8	1	2	1	1	1	0	2
1715	1	8	1	1	2	0	1	0	3
1717	1	9	2	2	1	2	1	0	1
1718	1	6	1	1	2	0	1	0	1
1720	0	4	1	0	1	0	1	0	1
1721	1	6	1	0	1	0	1	0	3
1722	1	18	2	3	3	2	2	3	3
1723	1	10	1	2	2	0	2	0	3
1725	1	9	2	2	1	0	1	0	3
1726	0	5	1	2	0	0	1	0	1
1729	1	7	1	1	1	0	2	0	2
1730	1	12	2	2	2	2	1	0	3
1731	0	4	1	0	0	0	1	0	2
1732	1	8	2	1	1	0	1	0	3
1733	1	10	2	1	2	1	1	0	3
1734	0	1	0	0	0	0	1	0	0
1735	0	4	1	0	0	0	1	0	2
1736	1	8	1	1	1	0	2	0	3
1737	1	11	2	1	1	0	2	3	2
1738	1	7	2	0	1	0	2	0	2
1739	0	3	1	0	1	0	1	0	0
1741	1	7	1	2	1	0	1	0	2
1742	0	3	0	0	1	0	0	0	2

1743	0	5	1	1	1	0	1	0	1
1744	1	9	2	1	2	0	1	0	3
1746	1	11	2	3	2	0	1	0	3
1748	1	7	2	2	1	0	1	0	1
1751	1	10	2	2	2	1	1	0	2
1752	1	11	3	3	2	0	1	0	2
1756	1	9	2	2	2	0	1	0	2
1757	1	8	2	2	1	0	2	0	1
1758	1	10	2	1	1	0	2	1	3
1759	1	7	1	2	0	0	1	0	3
1760	1	8	2	1	1	0	2	0	2
1761	1	7	1	2	1	0	1	0	2
1764	0	4	1	2	0	0	0	0	1
1765	1	9	2	2	2	1	1	0	1
1768	1	6	1	1	1	1	1	0	1
1770	1	9	2	2	1	1	1	0	2
1771	0	4	1	0	1	0	1	0	1
1773	0	5	0	1	1	0	2	0	1
1774	1	11	2	2	1	0	3	3	0
1775	1	11	2	3	1	0	2	1	2
1777	1	8	3	1	0	0	1	1	2
1779	1	11	3	0	3	2	1	0	2
1781	0	5	1	1	1	0	1	0	1
1782	1	12	2	2	1	0	2	2	3
1784	1	6	1	1	1	0	1	0	2
1785	1	13	2	3	1	0	2	2	3
1786	1	8	2	1	1	0	1	0	3
1787	1	14	3	3	1	0	2	3	2
1788	1	13	2	2	1	0	2	3	3
1789	1	13	2	3	1	0	2	2	3
1790	1	7	2	0	1	0	2	0	2
1791	1	6	2	1	1	0	1	0	1
1793	1	12	2	3	1	3	1	0	2
1795	1	7	1	2	1	0	1	0	2
1796	1	6	1	1	1	0	1	0	2
1798	1	6	1	0	1	0	1	0	3
1800	0	4	1	1	0	0	1	0	1
1801	1	6	1	1	1	1	1	0	1
1802	1	8	1	2	0	0	2	2	1
1804	1	9	2	3	1	0	1	0	2
1805	1	6	1	1	1	0	1	0	2
1806	1	6	1	1	1	0	1	0	2
1807	0	5	1	0	1	0	1	0	2
1808	1	8	1	1	0	0	1	3	2
1809	1	9	2	2	1	0	1	0	3
1811	0	3	0	1	0	0	1	0	1
1812	1	7	2	1	1	0	1	1	1
1813	1	9	1	3	1	0	2	0	2
1815	0	5	0	1	1	0	1	0	2
1816	1	12	2	2	2	1	1	2	2
1817	1	11	2	2	1	0	3	0	3
1818	1	7	2	0	1	0	2	0	2
1819	1	8	2	0	2	0	1	0	3
1820	0	5	1	1	1	0	1	0	1
1821	1	11	2	2	2	2	1	0	2
1822	1	11	1	2	3	1	1	0	3
1823	1	6	1	0	0	0	2	0	3
1825	1	10	3	1	2	1	1	0	2
1827	1	9	1	1	2	1	1	0	3
1828	0	5	1	1	1	0	1	0	1
1829	1	9	2	3	1	1	1	0	1
1830	0	3	1	0	1	0	0	0	1
1831	1	9	1	3	1	2	1	0	1
1832	1	11	2	2	1	0	2	2	2
1833	1	10	2	3	1	1	2	0	1
1834	1	8	1	2	1	1	1	0	2

1835	1	6	1	2	1	0	1	0	1
1837	1	6	1	0	1	1	1	0	2
1838	1	7	1	2	0	1	2	0	1
1841	1	9	2	2	1	0	1	0	3
1843	0	3	1	1	0	0	1	0	0
1844	1	7	1	1	1	0	2	1	1
1845	1	12	1	3	0	0	3	3	2
1846	1	8	2	1	1	0	2	1	1
1847	1	8	2	2	1	0	1	0	2
1848	1	10	3	0	2	0	2	0	3
1850	1	11	2	3	1	1	2	0	2
1851	1	14	3	2	1	0	3	3	2
1852	0	5	1	1	0	0	1	0	2
1854	1	11	2	2	2	0	2	0	3
1855	1	12	2	3	0	0	2	3	2
1856	1	6	1	0	1	0	1	0	3
1857	1	9	2	2	1	1	2	0	1
1858	1	8	2	2	0	0	1	0	3
1860	0	5	1	1	1	0	1	0	1
1861	1	7	1	2	2	0	2	0	0
1862	1	9	2	2	1	0	2	0	2
1863	1	11	3	2	3	1	1	0	1
1864	1	10	2	3	1	0	1	0	3
1865	1	8	2	2	1	0	1	0	2
1867	1	6	3	0	1	0	1	0	1
1868	1	6	1	2	0	0	1	0	2
1869	1	11	2	2	1	0	2	1	3
1870	1	6	2	1	1	0	1	0	1
1872	1	7	1	2	0	0	2	0	2
1876	1	8	2	1	0	0	2	0	3
1877	0	5	1	1	1	0	1	0	1
1878	1	10	2	3	0	1	1	1	2
1879	1	9	3	2	2	0	0	0	2
1880	0	5	1	2	0	0	1	0	1
1881	0	3	1	1	0	0	1	0	0
1883	1	8	1	1	1	0	2	0	3
1884	1	9	1	1	2	2	1	0	2
1885	0	3	0	0	0	0	1	0	2
1886	0	5	1	1	1	0	1	0	1
1890	1	12	3	2	3	0	1	0	3
1891	0	5	1	0	1	0	1	0	2
1893	1	11	3	2	2	0	2	1	1
1894	1	14	2	3	3	2	2	0	2
1896	1	13	2	0	3	2	1	3	2
1899	1	11	2	2	1	0	3	0	3
1900	1	7	1	2	0	0	1	0	3
1901	1	7	2	1	1	0	0	0	3
1902	1	9	2	1	2	0	2	0	2
1903	1	6	1	1	1	0	1	0	2
1904	1	8	2	2	0	0	2	0	2
1905	1	8	1	3	1	0	1	1	1
1908	0	5	1	1	0	0	2	0	1
1909	0	5	1	0	1	0	1	1	1
1910	1	16	3	3	3	3	1	0	3
1912	1	8	1	3	1	1	1	0	1
1913	1	7	1	1	1	2	1	0	1
1914	0	5	1	0	1	1	1	0	1
1915	0	2	0	0	0	0	1	0	1
1916	1	10	2	2	1	0	2	0	3
1917	0	4	1	0	1	0	1	0	1
1919	1	7	2	2	1	0	1	0	1
1920	1	16	3	3	2	1	2	3	2
1921	1	9	2	3	1	0	1	0	2
1922	0	5	1	1	1	0	1	0	1
1924	0	5	1	2	0	0	1	0	1
1925	0	5	2	0	1	0	0	0	2

1926	0	4	1	0	0	0	1	0	2
1929	0	5	1	0	1	1	1	0	1
1930	1	6	2	2	1	0	1	0	0
1931	1	11	3	0	2	1	1	1	3
1932	1	11	2	3	2	0	1	1	2
1933	1	10	2	3	1	0	1	0	3
1934	1	10	2	2	1	0	2	0	3
1936	1	8	2	2	1	0	1	0	2
1937	1	11	2	1	3	1	2	0	2
1938	1	8	2	2	1	0	1	0	2
1939	1	9	2	2	1	0	1	0	3
1940	1	9	2	1	3	0	1	0	2
1941	1	9	2	1	2	0	1	0	3
1942	1	8	1	1	1	0	2	0	3
1943	1	6	1	1	1	0	1	0	2
1944	1	9	1	2	1	1	1	0	3
1945	1	7	1	1	2	0	1	0	2
1946	1	7	2	1	1	0	1	0	2
1947	0	5	1	1	1	0	1	0	1
1950	0	4	0	0	1	0	1	0	2
1951	1	10	2	2	1	0	2	0	3
1952	1	8	2	1	2	0	1	0	2
1953	1	10	1	1	1	0	3	2	2
1954	0	5	1	0	1	0	1	0	2
1955	1	11	1	2	0	1	2	3	2
1956	1	8	1	3	1	1	1	0	1
1957	1	13	2	3	2	1	2	1	2
1958	1	6	2	2	1	0	0	0	1
1959	0	4	1	0	1	0	1	0	1
1961	0	5	1	0	1	0	1	0	2
1962	0	4	1	0	1	0	1	0	1
1963	1	9	1	3	1	0	1	0	3
1964	1	6	1	2	1	0	1	0	1
1965	1	9	2	1	1	0	2	0	3
1966	1	13	3	2	2	0	2	1	3
1968	0	5	1	1	1	0	1	0	1
1969	1	6	2	0	2	0	1	0	1
1970	0	3	0	1	0	0	1	0	1
1971	1	13	2	3	2	1	1	1	3
1972	1	8	2	2	0	0	2	0	2
1973	0	5	1	1	1	0	1	0	1
1974	0	5	1	0	1	1	1	0	1
1976	1	9	2	1	1	1	1	0	3
1977	1	7	2	2	0	1	1	0	1
1978	1	7	2	1	1	0	1	0	2
1979	1	8	2	3	1	0	1	0	1
1980	1	9	2	1	3	0	1	0	2
1981	1	6	2	1	1	0	1	0	1
1982	1	7	1	2	1	0	1	1	1
1984	1	9	3	0	2	0	1	0	3
1985	1	9	3	0	2	0	1	0	3
1986	0	5	1	1	1	0	1	0	1
1987	0	5	1	1	1	0	1	0	1
1988	0	3	1	0	1	0	1	0	0
1989	1	6	1	1	1	0	1	0	2
1990	0	5	2	1	0	0	1	0	1
1993	0	5	2	1	1	0	1	0	0
1994	0	5	1	1	1	0	1	0	1
1995	1	8	1	3	1	0	2	0	1
1996	1	11	2	0	3	2	2	0	2
1997	0	5	1	0	1	1	1	0	1
1998	1	7	1	2	1	0	2	0	1
2000	1	15	3	3	1	0	2	3	3
2001	1	16	3	2	3	2	2	1	3
2003	1	10	2	2	1	2	2	0	1
2004	1	9	1	2	1	1	1	2	1

2005	1	6	1	0	0	0	2	1	2
2006	0	3	0	1	0	0	1	0	1
2009	1	6	0	0	2	1	0	0	3
2010	1	10	2	2	1	0	2	0	3
2012	1	14	3	2	2	0	2	3	2
2014	1	7	1	1	1	0	1	1	2
2015	0	2	0	0	0	0	1	0	1
2016	0	3	0	1	0	0	1	0	1
2017	1	7	1	2	1	0	1	0	2
2019	1	9	2	1	1	1	2	0	2
2020	1	10	1	2	1	0	3	0	3
2022	1	7	1	3	0	0	1	1	1
2024	0	3	0	0	0	0	1	0	2
2026	1	10	2	2	2	0	1	0	3
2027	1	9	2	0	3	1	2	0	1
2028	1	17	2	3	3	2	2	2	3
2029	1	10	1	1	1	0	2	3	2
2030	1	9	2	1	1	1	1	1	2
2031	1	6	1	1	1	0	1	0	2
2032	1	10	1	2	1	0	1	3	2
2033	1	8	3	1	0	0	1	0	3
2034	1	9	2	2	1	1	1	0	2
2035	1	6	1	1	1	0	1	0	2
2037	1	7	1	1	2	0	2	0	1
2038	1	11	1	3	2	0	2	1	2
2039	1	15	3	3	1	0	2	3	3
2040	1	11	2	3	1	2	1	0	2
2041	1	7	1	1	1	0	2	0	2
2042	0	3	0	1	1	0	1	0	0
2043	0	5	1	1	1	0	2	0	0
2044	1	10	2	2	1	0	3	0	2
2045	1	10	2	1	1	0	1	2	3
2046	1	6	1	2	1	0	1	0	1
2047	1	15	2	3	2	3	2	1	2
2049	1	6	1	1	1	0	1	0	2
2050	0	2	0	0	0	0	1	0	1
2051	1	9	2	2	1	0	2	0	2
2052	1	9	2	2	1	1	2	0	1
2053	1	7	1	2	0	0	2	0	2
2056	0	4	1	0	1	0	1	0	1
2057	1	10	2	2	1	0	1	1	3
2058	1	11	2	3	1	0	2	1	2
2060	0	4	1	0	1	0	1	0	1
2061	1	6	1	1	1	0	2	0	1
2062	1	12	2	3	3	0	2	0	2
2065	1	8	2	2	1	0	1	0	2
2067	1	6	1	1	1	0	1	0	2
2070	1	8	1	2	1	0	2	0	2
2071	1	15	3	2	2	0	2	3	3
2073	0	5	1	0	1	0	1	0	2
2075	1	10	2	2	1	0	2	1	2
2076	1	9	2	2	0	1	2	0	2
2079	1	12	2	3	2	1	2	0	2
2080	1	7	1	1	1	0	1	0	3
2082	0	4	0	0	1	0	1	1	1
2083	1	7	1	2	1	0	1	0	2
2085	1	12	1	3	1	0	2	3	2
2087	1	11	2	3	2	1	1	0	2
2090	1	10	2	3	1	0	2	0	2
2091	1	6	1	1	0	0	2	0	2
2093	1	7	1	2	0	0	1	0	3
2094	0	4	0	1	1	0	1	0	1
2095	1	7	1	1	0	0	1	2	2
2096	1	10	1	3	1	0	1	3	1
2097	1	9	2	1	1	0	2	0	3
2098	1	14	2	2	1	0	3	3	3

2099	1	14	3	3	1	1	2	1	3
2100	1	8	2	2	1	0	1	0	2
2102	1	6	2	0	1	0	1	0	2
2103	1	10	2	2	1	0	2	1	2
2105	1	7	1	1	1	0	1	0	3
2106	1	10	2	1	3	0	1	1	2
2108	0	3	1	0	0	0	1	0	1
2109	1	7	1	3	1	0	0	0	2
2110	1	12	2	3	3	0	2	1	1
2112	1	7	1	2	0	0	1	0	3
2113	1	7	2	0	1	0	1	0	3
2114	1	9	2	2	1	1	1	0	2
2116	1	7	2	2	1	0	1	0	1
2117	1	6	1	1	1	0	1	0	2
2118	0	5	1	0	1	0	1	0	2
2120	1	12	1	2	1	0	2	3	3
2121	1	12	2	3	3	0	2	0	2
2122	1	9	2	1	2	0	2	0	2
2123	1	8	1	3	1	1	1	0	1
2124	0	3	0	0	1	0	1	0	1
2125	1	6	2	1	1	0	1	0	1
2126	1	13	1	3	1	1	2	3	2
2127	1	7	1	1	1	0	2	0	2
2128	1	6	1	1	1	0	2	0	1
2129	1	12	3	2	0	0	2	2	3
2132	1	6	1	2	1	0	1	0	1
2133	1	8	1	2	1	0	2	0	2
2136	1	8	2	1	2	0	1	0	2
2138	0	4	1	0	1	0	1	0	1
2139	1	9	2	1	2	1	1	0	2
2140	1	14	2	3	2	2	1	2	2
2141	0	5	1	1	0	0	1	0	2
2142	1	15	3	2	2	2	2	1	3
2143	1	6	1	1	0	0	1	0	3
2144	1	10	2	3	2	0	1	0	2
2145	1	6	0	2	0	0	1	0	3
2146	1	13	2	3	1	0	2	3	2
2147	1	13	1	2	2	3	1	3	1
2148	1	8	1	0	2	1	1	1	2
2149	1	9	1	3	1	0	1	0	3
2150	1	9	2	3	1	1	1	0	1
2151	1	10	2	1	1	1	1	2	2
2153	1	7	1	1	1	0	2	0	2
2154	1	10	1	1	3	1	1	0	3
2157	0	1	0	0	0	0	1	0	0
2158	1	11	2	2	1	1	2	0	3
2159	1	6	1	1	1	0	1	0	2
2160	1	6	0	1	1	1	1	0	2
2162	1	6	1	1	1	0	1	0	2
2163	1	9	1	3	1	0	2	0	2
2164	1	7	1	2	1	0	1	0	2
2165	0	5	1	1	1	0	1	0	1
2166	0	5	1	1	1	0	1	0	1
2169	1	6	1	2	0	0	1	0	2
2170	1	10	2	1	2	1	2	0	2
2171	0	4	1	0	0	0	2	0	1
2172	1	6	2	1	0	0	2	0	1
2173	0	5	1	1	1	0	1	0	1
2174	1	9	2	2	1	0	2	0	2
2175	1	8	3	0	2	0	1	0	2
2176	1	11	3	1	2	0	2	0	3
2177	1	8	1	1	3	0	1	0	2
2178	1	16	3	3	3	0	2	2	3
2179	1	12	2	3	1	1	2	1	2
2180	1	7	2	0	1	0	1	0	3
2181	1	12	2	1	2	3	2	0	2

2185	1	13	3	1	2	2	2	0	3
2186	1	9	1	2	1	0	1	3	1
2188	1	12	3	2	3	0	2	0	2
2190	1	8	1	3	1	0	1	0	2
2191	1	7	1	2	1	0	2	0	1
2192	0	4	1	0	1	0	1	0	1
2193	0	5	1	0	1	0	0	0	3
2194	1	7	1	0	2	0	1	0	3
2196	1	10	3	2	1	0	2	0	2
2197	1	9	2	1	1	1	2	0	2
2198	1	8	1	2	1	0	1	0	3
2199	1	13	2	3	2	2	1	0	3
2200	0	5	1	1	1	0	1	0	1
2201	1	10	2	2	1	0	2	0	3
2202	1	10	1	2	1	0	1	3	2
2205	0	5	1	1	1	0	1	0	1
2206	1	6	2	1	1	0	1	0	1
2207	1	9	2	1	2	0	1	0	3
2208	1	9	2	1	2	0	2	0	2
2210	1	7	1	2	1	0	1	0	2
2212	0	5	1	1	1	0	1	0	1
2214	1	13	2	1	2	0	2	3	3
2216	1	9	1	1	1	0	1	3	2
2217	1	8	1	1	2	1	1	0	2
2219	1	7	1	2	1	1	1	0	1
2220	0	2	0	0	0	0	1	0	1
2221	1	8	2	2	1	1	1	0	1
2222	1	16	3	3	2	3	1	2	2
2223	1	6	1	1	1	0	1	0	2
2224	0	4	0	1	1	0	1	0	1
2225	1	8	1	1	2	0	1	0	3
2226	1	8	1	1	1	2	1	0	2
2227	1	8	1	1	1	0	2	0	3
2229	0	4	0	1	1	0	1	0	1
2230	0	4	1	0	1	0	1	0	1
2231	1	10	2	2	1	0	2	1	2
2232	1	13	3	2	1	0	2	2	3
2234	1	8	2	1	1	0	1	0	3
2237	1	8	2	2	1	0	1	0	2
2238	1	9	2	1	1	0	2	0	3
2240	1	11	2	2	2	1	2	0	2
2241	1	6	1	0	1	0	1	0	3
2242	0	2	1	0	1	0	0	0	0
2243	0	4	1	0	2	0	1	0	0
2245	1	7	2	2	0	0	1	1	1
2247	1	6	1	2	1	0	1	0	1
2248	0	4	2	0	1	0	1	0	0
2249	1	13	3	2	2	0	2	1	3
2250	1	9	2	2	0	0	2	0	3
2252	0	5	1	1	1	0	1	0	1
2254	1	8	2	2	1	0	1	0	2
2255	1	9	2	2	1	0	1	0	3
2256	1	7	1	2	1	0	1	0	2
2257	1	8	1	2	2	0	1	0	2
2258	1	9	2	1	3	0	1	0	2
2259	1	9	1	2	1	1	1	0	3
2260	1	7	1	1	1	1	1	1	1
2261	0	4	0	0	1	0	1	0	2
2262	1	11	2	3	1	0	2	0	3
2263	1	12	2	2	2	0	2	2	2
2265	1	8	1	2	1	0	2	0	2
2267	1	10	2	0	2	0	3	0	3
2268	1	9	2	1	1	1	2	0	2
2270	1	8	2	1	1	0	1	1	2
2271	1	8	1	1	1	0	2	0	3
2272	1	11	1	3	1	1	2	1	2

2273	1	10	1	2	1	0	2	3	1
2274	0	5	1	1	1	0	1	0	1
2275	1	11	2	2	2	0	2	0	3
2276	1	12	3	2	2	0	2	0	3
2277	1	12	2	3	1	1	2	0	3
2279	0	5	1	1	0	0	2	0	1
2283	1	7	1	1	1	0	2	0	2
2284	1	10	2	2	1	0	1	2	2
2285	1	6	0	0	2	0	1	0	3
2286	1	9	2	0	2	1	1	0	3
2287	1	11	3	2	2	0	1	0	3
2288	0	4	1	1	1	0	0	0	1
2289	1	9	2	1	1	0	2	0	3
2290	1	8	1	2	1	0	1	1	2
2291	1	11	2	2	2	0	2	0	3
2293	1	9	1	3	1	0	1	2	1
2295	1	8	1	1	1	0	1	2	2
2296	1	7	2	1	1	0	2	0	1
2298	1	9	2	1	2	0	2	0	2
2300	0	5	1	2	0	0	1	0	1
2301	1	12	2	1	2	0	1	3	3
2302	1	6	1	1	1	0	1	0	2
2303	1	6	1	2	0	0	1	2	0
2304	1	6	1	2	0	0	1	0	2
2305	1	8	2	1	1	0	2	0	2
2306	1	11	1	3	2	2	1	1	1
2307	1	10	2	0	2	1	2	0	3
2308	0	3	0	1	0	0	1	0	1
2309	1	12	2	3	2	0	2	0	3
2310	1	8	2	1	1	0	1	1	2
2311	0	4	1	1	0	0	1	0	1
2312	0	4	1	1	0	0	1	0	1
2314	1	14	2	2	1	1	2	3	3
2315	1	6	1	1	1	0	1	0	2
2316	1	7	2	0	1	0	2	0	2
2317	0	3	1	0	0	0	1	0	1
2319	1	8	1	1	1	0	1	1	3
2320	0	5	1	0	1	0	1	0	2
2321	1	6	1	2	0	0	1	0	2
2322	1	7	1	1	1	1	1	0	2
2323	0	3	0	0	1	0	1	0	1
2325	1	10	1	1	1	0	2	3	2
2326	0	4	1	0	1	0	1	0	1
2327	1	12	2	2	2	1	2	0	3
2328	0	4	0	2	0	0	1	0	1
2329	0	4	1	1	1	0	0	0	1
2330	1	13	2	3	2	1	2	1	2
2331	1	9	2	2	1	0	1	1	2
2332	1	8	2	2	1	0	1	0	2
2334	1	7	2	0	1	1	1	0	2
2335	1	10	2	3	1	0	1	0	3
2337	1	6	1	2	1	0	1	0	1
2338	0	5	2	1	0	0	1	0	1
2339	1	9	1	2	1	0	1	3	1
2340	1	6	2	1	0	0	2	0	1
2342	1	9	2	2	1	0	1	0	3
2343	0	3	0	0	1	0	1	0	1
2344	1	13	3	3	2	0	2	0	3
2346	0	4	1	0	1	0	1	0	1
2347	1	14	3	2	1	0	2	3	3
2348	1	13	3	3	1	0	2	3	1
2349	1	10	3	3	0	0	1	2	1
2350	0	5	1	1	1	0	1	0	1
2351	1	9	2	1	1	0	2	2	1
2352	1	19	3	3	2	3	2	3	3
2354	1	9	1	3	1	0	2	0	2

2355	1	7	1	1	1	0	2	0	2
2357	0	5	1	1	0	0	2	0	1
2358	1	14	3	3	2	0	1	3	2
2359	1	12	3	2	2	0	2	1	2
2361	1	7	0	1	1	0	2	0	3
2362	1	7	3	1	0	0	1	0	2
2363	1	12	2	3	2	1	2	0	2
2365	1	6	2	0	1	0	1	0	2
2366	1	7	2	0	2	0	1	0	2
2367	1	12	2	2	1	1	3	1	2
2369	1	7	2	1	1	0	1	0	2
2370	0	5	1	0	1	0	1	0	2
2371	1	6	1	1	1	0	2	0	1
2373	1	8	2	2	2	0	1	0	1
2374	1	8	1	2	1	0	2	0	2
2376	1	7	1	1	1	0	1	0	3
2377	1	6	1	2	1	0	1	0	1
2378	1	6	1	1	1	0	2	0	1
2379	1	6	1	1	1	1	1	0	1
2381	0	5	1	1	1	0	1	0	1
2383	1	10	2	2	1	0	1	1	3
2384	0	5	1	0	1	0	1	0	2
2385	1	9	2	1	1	0	2	0	3
2386	0	5	2	0	1	0	1	0	1
2388	1	7	2	0	1	0	2	0	2
2391	1	10	2	1	2	0	2	0	3
2393	1	7	2	2	1	0	1	0	1
2394	0	4	0	1	0	0	1	1	1
2395	1	15	3	3	1	0	3	2	3
2397	0	5	1	0	2	0	1	0	1
2398	1	9	2	1	2	1	1	0	2
2399	1	7	1	1	0	0	2	1	2
2400	1	6	1	0	3	1	1	0	0
2401	1	7	1	1	1	0	2	0	2
2403	1	7	2	1	1	0	1	0	2
2406	1	7	1	2	1	0	1	0	2
2407	0	5	1	2	1	0	0	0	1
2408	0	5	2	0	1	0	1	0	1
2409	0	4	1	0	1	0	0	0	2
2410	0	5	0	0	1	1	1	0	2
2411	1	11	1	3	1	2	2	0	2
2414	0	2	1	0	0	0	0	0	1
2415	1	6	1	1	1	0	1	0	2
2416	0	3	1	0	0	0	1	0	1
2417	1	8	2	2	1	0	1	0	2
2418	0	4	1	0	1	0	1	0	1
2420	0	5	1	1	1	0	1	0	1
2421	1	6	1	1	1	0	1	0	2
2422	0	3	0	0	1	0	1	0	1
2423	1	8	1	1	0	0	1	3	2
2425	1	6	1	1	1	0	1	0	2
2426	1	9	2	2	1	0	2	0	2
2427	1	6	1	2	1	0	1	0	1
2429	1	7	1	2	0	0	1	0	3
2431	1	6	2	1	1	0	1	0	1
2433	0	5	1	1	0	0	2	0	1
2434	0	5	2	0	1	0	1	0	1
2435	1	6	1	1	0	0	2	0	2
2436	1	11	3	1	1	2	1	0	3
2437	1	10	1	3	2	1	2	0	1
2438	1	7	1	0	1	1	1	0	3
2439	0	4	1	1	0	0	1	0	1
2441	1	6	1	1	1	0	1	0	2
2442	1	11	3	3	1	0	1	0	3
2443	1	9	2	2	1	1	1	0	2
2445	1	9	2	1	1	0	2	0	3

2447	0	5	1	1	0	0	1	0	2
2449	1	7	1	1	1	0	2	0	2
2450	1	9	1	1	1	2	2	0	2
2452	0	5	2	1	0	0	1	0	1
2453	1	10	1	2	1	0	2	1	3
2454	1	11	3	1	2	0	2	0	3
2456	1	18	3	2	3	3	3	2	2
2458	1	8	1	1	1	1	2	0	2
2459	0	3	0	0	1	0	1	0	1
2460	1	8	2	1	1	0	2	0	2
2461	0	4	1	1	0	0	1	0	1
2463	1	8	1	2	1	0	2	0	2
2464	0	5	1	1	0	0	1	0	2
2465	0	5	1	0	1	0	1	0	2
2466	1	11	1	1	1	1	1	3	3
2467	1	16	2	3	2	1	2	3	3
2468	1	11	1	3	1	1	2	0	3
2469	1	9	1	2	1	0	2	0	3
2470	1	6	1	2	1	0	1	0	1
2471	1	9	2	2	1	0	1	1	2
2472	1	7	1	1	1	0	1	0	3
2473	1	8	2	1	2	0	1	1	1
2474	1	8	1	1	1	0	2	0	3
2475	1	8	2	2	2	0	1	0	1
2476	1	6	1	1	2	0	1	0	1
2477	1	8	2	2	0	0	1	0	3
2480	0	5	1	0	1	0	1	0	2
2481	1	6	0	2	1	1	1	0	1
2482	1	6	1	0	2	0	1	0	2
2483	0	4	0	0	1	0	1	0	2
2485	1	7	2	1	1	0	1	0	2
2488	1	14	2	2	3	0	2	3	2
2489	1	17	2	3	3	3	2	2	2
2490	1	8	1	1	2	1	1	0	2
2491	1	7	1	2	1	0	1	0	2
2492	1	6	1	1	1	0	1	0	2
2493	1	9	1	2	2	0	2	0	2
2494	1	12	2	2	1	0	2	3	2
2495	1	10	1	2	2	0	2	0	3
2496	1	11	1	3	1	0	1	3	2
2497	1	12	2	2	2	1	3	0	2
2498	1	20	3	3	3	2	3	3	3
2499	1	12	2	3	2	0	2	1	2
2500	1	8	2	1	1	0	2	0	2
2501	1	12	2	2	2	3	1	0	2
2502	0	4	1	1	0	0	1	0	1
2503	1	15	2	3	1	0	3	3	3
2507	1	11	2	1	1	0	2	3	2
2508	1	10	1	1	2	1	2	0	3
2509	0	5	1	1	1	0	1	0	1
2510	1	12	2	1	1	2	3	1	2
2511	1	8	1	1	1	1	1	0	3
2514	1	12	2	3	1	1	2	0	3
2516	1	6	1	1	1	0	1	0	2
2517	1	7	1	1	2	0	1	0	2
2519	1	13	2	3	1	1	2	2	2
2521	1	13	2	3	1	0	2	2	3
2523	0	5	1	0	1	0	1	0	2
2524	0	5	1	1	0	1	1	0	1
2526	1	11	2	2	1	0	3	0	3
2527	1	7	2	1	1	0	1	0	2
2529	0	2	0	0	1	0	0	0	1
2532	1	7	2	1	1	0	2	0	1
2533	1	9	2	2	1	0	2	0	2
2535	1	12	1	2	1	0	2	3	3
2537	1	6	1	1	1	1	1	0	1

2538	0	5	1	1	1	0	1	0	1
2539	1	9	1	2	1	0	1	2	2
2540	1	10	2	0	3	1	1	0	3
2541	1	7	2	1	0	0	2	0	2
2542	0	4	0	1	1	0	1	0	1
2546	1	7	1	2	1	0	1	0	2
2548	1	6	1	0	1	0	1	0	3
2549	1	9	1	1	1	0	2	1	3
2550	0	5	1	1	1	0	1	0	1
2551	0	5	1	1	0	0	1	0	2
2552	1	8	1	2	1	0	2	0	2
2553	1	16	2	3	2	1	2	3	3
2554	0	5	1	1	1	0	1	0	1
2555	1	9	2	1	1	0	2	0	3
2556	1	10	2	2	1	0	2	0	3
2557	1	12	2	2	1	0	3	1	3
2558	1	9	3	0	3	0	0	0	3
2559	0	4	1	0	0	0	2	0	1
2560	1	6	1	0	1	1	1	0	2
2561	1	8	1	2	1	0	1	0	3
2562	1	10	2	2	3	0	2	0	1
2564	0	4	1	1	0	0	1	0	1
2566	1	7	1	1	1	0	1	0	3
2568	0	4	1	0	1	0	0	0	2
2569	1	9	2	1	1	0	2	0	3
2570	1	12	3	3	1	0	2	1	2
2571	1	8	2	1	2	0	1	0	2
2572	1	8	1	2	1	0	1	0	3
2573	1	10	2	2	1	0	2	0	3
2574	1	11	1	2	1	0	1	3	3
2575	1	9	2	1	1	0	2	0	3
2579	1	9	2	1	1	0	2	0	3
2580	1	9	2	2	1	1	1	0	2
2581	1	6	1	0	1	0	1	0	3
2582	1	6	1	1	1	0	1	0	2
2583	1	8	2	1	2	0	1	0	2
2584	0	4	1	0	1	0	0	0	2
2586	0	5	2	0	1	0	1	0	1
2587	1	7	2	1	1	0	1	0	2
2588	1	7	1	1	0	0	2	0	3
2589	1	6	0	2	0	0	1	0	3
2590	1	6	0	0	1	1	1	0	3
2591	1	15	3	3	0	0	3	3	3
2592	1	6	2	0	1	0	2	0	1
2593	1	10	3	1	1	1	2	0	2
2594	0	5	1	1	0	0	1	0	2
2595	0	5	1	0	1	0	1	0	2
2596	1	8	2	0	1	0	2	0	3
2598	1	12	2	3	1	0	2	2	2
2599	1	7	1	0	1	0	1	3	1
2600	1	9	2	2	1	0	2	0	2
2601	0	5	0	1	1	0	1	0	2
2603	1	13	2	2	2	0	1	3	3
2606	1	6	1	1	1	0	1	0	2
2608	1	6	1	2	0	0	2	0	1
2609	0	1	0	0	0	0	1	0	0
2611	1	12	2	1	2	0	1	3	3
2612	1	7	1	1	1	1	1	0	2
2614	1	13	3	1	1	0	2	3	3
2615	1	7	1	2	1	0	1	0	2
2616	1	11	2	2	1	0	2	2	2
2617	0	3	0	0	1	0	1	0	1
2618	1	6	2	1	1	0	1	0	1
2619	1	7	1	0	2	0	2	0	2
2620	0	3	1	0	0	0	1	0	1
2621	1	6	1	1	1	0	1	0	2

2622	1	15	2	3	0	2	2	3	3
2624	1	7	2	1	1	0	1	0	2
2625	1	8	1	2	2	0	2	0	1
2626	0	5	1	1	1	0	1	0	1
2627	0	4	1	1	0	0	1	0	1
2628	1	11	2	2	0	0	3	1	3
2629	0	5	2	0	0	0	2	0	1
2631	1	9	2	1	2	0	1	3	0
2632	1	6	2	0	1	0	1	0	2
2633	1	8	1	1	1	0	2	0	3
2634	0	5	1	0	1	0	1	0	2
2635	1	6	1	3	0	0	1	0	1
2636	0	3	0	1	0	0	1	0	1
2638	1	7	2	1	1	0	2	0	1
2639	1	11	2	3	1	1	2	0	2
2640	1	7	1	2	0	0	2	0	2
2642	1	7	2	1	1	0	1	0	2
2643	1	10	2	2	2	0	2	0	2
2644	0	3	1	0	0	0	1	0	1
2645	1	7	1	1	1	0	2	0	2
2646	1	7	1	2	1	0	1	0	2
2648	1	12	2	2	1	2	2	0	3
2649	1	9	2	0	3	0	2	0	2
2650	1	11	2	3	0	0	1	2	3
2651	1	9	2	3	1	0	1	1	1
2654	1	7	1	2	1	0	1	0	2
2655	1	9	1	1	1	2	1	0	3
2656	1	14	3	3	3	0	1	2	2
2658	0	5	1	0	1	0	1	0	2
2659	0	2	0	1	0	0	1	0	0
2661	1	12	2	3	2	2	1	0	2
2667	1	6	2	1	2	0	1	0	0
2668	1	11	3	3	2	0	0	0	3
2669	1	6	1	1	1	0	1	0	2
2670	1	9	3	0	3	0	0	0	3
2671	1	6	0	0	0	0	1	3	2
2672	1	12	2	2	1	0	2	3	2
2673	0	3	1	0	0	0	1	0	1
2674	1	11	2	3	3	1	1	0	1
2676	1	8	2	0	2	1	1	0	2
2677	1	6	1	3	0	0	1	0	1
2678	1	7	1	0	1	2	1	0	2
2679	1	13	3	2	2	2	1	0	3
2680	1	9	2	3	1	0	1	0	2
2681	1	7	1	1	1	0	1	0	3
2682	1	6	1	1	1	0	1	0	2
2683	1	6	1	2	0	0	1	0	2
2684	0	3	0	0	0	0	1	0	2
2685	1	6	0	0	0	0	1	3	2
2687	0	5	1	0	1	0	2	0	1
2689	0	5	1	1	1	0	1	0	1
2691	1	8	2	1	1	0	2	0	2
2692	1	11	3	2	3	0	1	0	2
2694	1	10	2	1	2	2	1	0	2
2695	1	11	1	3	2	2	1	0	2
2696	1	6	1	2	1	0	1	0	1
2697	1	7	1	1	2	1	1	0	1
2698	1	6	1	1	1	0	1	0	2
2699	1	6	1	2	1	0	1	0	1
2700	1	11	2	3	1	1	2	0	2
2701	1	10	1	2	2	1	3	0	1
2702	1	11	2	2	1	1	2	1	2
2703	1	10	1	2	1	2	1	0	3
2707	0	4	1	1	0	0	1	0	1
2708	1	8	2	2	1	0	1	0	2
2710	1	6	1	1	1	0	1	0	2

2711	0	4	0	1	1	0	1	0	1
2715	0	3	1	0	0	0	1	0	1
2716	1	8	2	1	1	0	1	0	3
2717	1	9	2	1	1	0	2	0	3
2719	1	15	3	2	3	3	2	1	1
2720	1	9	1	2	1	0	1	1	3
2721	1	8	2	1	2	0	1	0	2
2722	1	15	2	2	3	3	1	1	3
2723	1	11	2	3	1	0	2	0	3
2724	0	3	0	0	1	0	1	0	1
2725	0	5	1	2	1	0	1	0	0
2726	1	9	2	2	2	0	1	0	2
2727	1	12	3	3	1	1	2	1	1
2728	1	10	2	2	1	2	1	0	2
2729	1	6	1	1	1	0	1	0	2
2730	1	8	2	2	2	0	1	0	1
2731	1	6	1	1	1	0	1	0	2
2733	1	7	1	2	0	0	2	0	2
2735	0	5	1	0	1	0	1	0	2
2736	0	4	1	0	0	0	1	0	2
2738	0	5	1	1	1	0	1	0	1
2739	1	7	1	0	2	0	2	0	2
2741	1	8	2	1	1	1	1	0	2
2742	1	10	2	3	1	0	1	1	2
2743	0	4	0	1	1	0	1	0	1
2744	1	10	2	2	0	0	2	2	2
2745	1	10	2	2	1	1	1	0	3
2749	1	7	1	1	1	0	1	1	2
2750	0	2	0	0	0	0	1	0	1
2751	1	8	2	1	1	0	2	0	2
2753	1	7	2	2	0	0	1	0	2
2754	1	9	2	1	2	0	2	0	2
2756	0	4	0	2	0	0	0	0	2
2757	1	8	1	2	1	0	1	3	0
2758	1	7	1	2	1	0	2	0	1
2759	1	13	3	2	2	0	2	1	3
2760	1	8	2	1	2	0	1	0	2
2761	1	6	1	0	2	0	1	0	2
2762	1	9	2	2	1	1	1	0	2
2763	1	6	1	1	1	0	1	0	2
2764	1	9	3	1	1	0	1	0	3
2765	1	7	1	2	1	0	1	0	2
2766	0	5	1	1	0	0	1	0	2
2767	1	7	1	3	0	0	1	0	2
2769	1	9	1	1	2	2	2	0	1
2770	0	1	0	0	1	0	0	0	0
2771	0	5	1	1	0	0	1	1	1
2772	1	11	2	1	1	0	2	3	2
2773	1	10	2	1	2	0	2	0	3
2776	1	6	2	0	1	0	1	0	2
2777	0	5	1	1	1	0	1	0	1
2782	1	9	2	2	1	0	2	0	2
2783	1	11	1	2	2	3	2	0	1
2784	0	5	1	1	1	0	1	0	1
2786	1	9	1	0	1	0	1	3	3
2787	1	8	1	1	1	0	2	0	3
2788	1	9	1	1	2	0	3	0	2
2789	1	7	2	1	1	0	1	1	1
2790	1	13	2	2	1	0	2	3	3
2792	0	3	1	0	0	0	1	0	1
2793	1	6	1	2	0	0	1	0	2
2794	1	7	2	1	0	0	2	0	2
2795	1	10	2	2	2	0	2	0	2
2797	0	3	0	1	0	0	1	0	1
2798	0	4	1	0	1	0	1	0	1
2799	0	5	1	1	0	0	1	0	2

2800	1	8	2	0	1	0	2	0	3
2801	1	12	2	3	2	1	2	0	2
2802	0	0	0	0	0	0	0	0	0
2803	1	10	2	2	1	0	2	0	3
2804	1	10	2	2	1	0	2	0	3
2806	1	8	2	3	1	0	1	0	1
2807	1	7	1	2	1	0	1	0	2
2810	1	6	1	0	0	0	1	3	1
2811	0	4	1	1	0	0	1	0	1
2812	1	8	2	2	0	0	2	0	2
2813	1	12	2	3	1	0	2	1	3
2814	0	5	1	1	1	0	2	0	0
2815	0	4	1	0	0	0	1	0	2
2817	1	8	1	3	1	0	2	0	1
2818	1	7	1	2	1	0	2	0	1
2819	1	8	1	1	1	0	2	1	2
2820	1	9	2	2	1	0	2	0	2
2821	1	9	1	2	1	0	1	2	2
2822	1	14	2	3	1	1	2	2	3
2823	1	6	1	1	1	0	1	0	2
2826	1	10	2	1	2	1	2	0	2
2827	1	12	3	2	1	1	2	0	3
2828	1	6	1	2	0	0	1	0	2
2830	1	7	1	1	1	0	2	0	2
2831	1	10	1	3	2	0	1	0	3
2833	0	5	1	0	1	0	1	0	2
2834	0	5	1	0	0	0	1	0	3
2835	1	8	1	2	1	2	1	0	1
2836	1	6	1	1	1	1	1	0	1
2837	1	14	2	2	2	1	2	2	3
2839	1	8	1	2	3	0	1	0	1
2840	1	13	1	3	3	2	2	0	2
2841	1	8	1	2	1	0	1	0	3
2842	1	10	2	2	1	0	1	1	3
2844	1	7	2	0	1	0	2	0	2
2845	1	9	1	2	2	1	1	0	2
2846	1	14	2	1	3	2	2	2	2
2847	0	4	1	0	0	0	1	0	2
2848	1	7	2	0	1	0	3	0	1
2849	1	12	2	2	1	0	2	2	3
2850	1	10	2	1	1	0	2	2	2
2851	1	10	2	3	1	0	1	0	3
2852	0	5	1	1	1	0	1	0	1
2854	1	10	3	0	2	0	2	0	3
2855	1	11	2	2	3	0	1	0	3
2856	1	11	2	1	1	0	2	3	2
2857	1	10	2	1	1	0	1	2	3
2859	1	12	3	3	2	0	2	0	2
2860	1	6	1	1	0	2	1	0	1
2861	1	8	1	2	2	0	1	0	2
2862	0	5	1	1	1	0	1	0	1
2863	1	6	1	1	1	0	1	0	2
2864	1	9	1	2	1	1	1	2	1
2869	1	6	1	1	1	0	1	0	2
2870	1	8	2	1	1	0	2	0	2
2871	1	12	2	1	2	0	2	3	2
2872	1	7	1	2	0	0	1	0	3
2873	1	7	2	1	1	0	1	0	2
2874	1	7	1	1	1	0	1	0	3
2876	0	5	1	0	1	0	1	0	2
2877	0	5	1	1	0	0	1	0	2
2878	1	7	2	2	0	0	1	0	2
2880	1	11	2	2	1	0	2	3	1
2881	1	8	1	1	1	1	1	0	3
2882	1	8	2	2	1	0	1	0	2
2883	0	4	0	1	1	0	1	0	1

2884	1	8	2	2	1	0	1	0	2
2886	1	9	2	2	0	0	2	1	2
2887	0	5	1	1	1	0	1	0	1
2888	1	12	2	2	2	1	1	2	2
2890	1	8	1	1	1	0	2	0	3
2891	1	11	1	3	0	0	2	2	3
2892	1	14	3	2	3	0	3	3	0
2893	1	11	2	3	1	0	2	2	1
2894	1	6	1	1	1	0	0	0	3
2895	0	3	0	0	0	0	1	0	2
2896	1	13	2	2	3	3	1	0	2
2898	1	8	1	2	1	0	1	0	3
2900	1	10	2	2	2	0	2	0	2
2901	0	4	1	0	1	0	1	0	1
2902	0	5	1	1	1	0	1	0	1
2904	1	6	1	2	1	0	1	0	1
2905	0	4	1	0	0	0	1	0	2
2906	1	6	1	0	1	0	2	0	2
2907	1	8	1	1	1	0	1	1	3
2908	1	6	1	1	1	0	1	0	2
2911	1	14	2	2	2	0	2	3	3
2912	1	11	2	1	3	3	1	0	1
2913	1	8	2	1	1	0	1	0	3
2914	1	7	1	1	0	0	2	2	1
2915	1	15	3	3	1	1	2	2	3
2916	1	10	2	3	0	0	2	1	2
2917	1	14	3	3	1	0	2	3	2
2918	1	8	2	2	1	0	1	0	2
2919	1	8	1	3	1	0	1	0	2
2920	1	6	1	0	1	0	1	0	3
2921	1	8	2	2	1	0	1	0	2
2922	1	9	1	3	1	0	1	0	3
2923	1	6	1	1	1	0	1	0	2
2924	1	10	2	2	1	0	2	1	2
2925	0	5	1	0	1	0	1	0	2
2926	0	5	1	1	1	0	1	0	1
2927	1	6	1	1	1	0	1	0	2
2928	0	4	1	0	1	0	1	0	1
2929	0	4	1	1	0	0	1	0	1
2930	1	12	1	2	2	0	3	1	3
2932	1	9	1	3	1	0	1	0	3
2933	0	3	1	0	1	0	1	0	0
2937	1	6	1	1	1	0	1	0	2
2939	0	3	0	0	2	0	0	0	1
2940	1	7	1	1	1	0	2	0	2
2941	1	6	1	2	0	0	2	0	1
2942	1	18	2	2	3	3	2	3	3
2944	1	14	2	3	2	1	2	1	3
2945	0	4	1	0	1	0	1	0	1
2946	1	8	2	2	1	1	1	0	1
2947	1	15	3	3	3	2	1	1	2
2948	1	8	2	2	1	0	1	0	2
2950	0	3	1	0	0	0	1	0	1
2951	0	4	1	1	1	0	1	0	0
2952	1	9	2	1	1	0	2	1	2
2953	0	1	0	0	0	0	0	0	1
2955	1	6	1	2	1	0	1	0	1
2956	0	2	0	0	0	0	1	0	1
2957	1	8	1	1	1	0	1	2	2
2958	1	6	1	1	1	0	1	0	2
2959	1	9	1	2	1	0	2	1	2
2960	1	6	1	1	1	0	1	0	2
2962	1	12	2	3	2	2	2	0	1
2963	1	7	2	1	1	0	1	0	2
2964	1	18	3	3	3	1	2	3	3
2965	1	6	1	2	1	0	1	0	1

2966	1	8	1	2	0	0	2	1	2
2967	0	4	1	1	1	0	1	0	0
2968	1	11	2	1	1	0	1	3	3
2969	1	6	1	2	1	0	1	0	1
2970	1	6	1	2	1	0	1	0	1
2971	1	11	2	3	1	1	2	0	2
2972	1	9	2	3	1	0	1	0	2
2973	1	9	2	1	1	1	1	0	3
2974	1	11	3	2	1	0	1	2	2
2977	1	9	1	3	1	0	2	0	2
2978	1	8	1	1	2	2	1	0	1
2979	0	3	1	1	0	0	0	0	1
2980	0	5	1	0	1	0	1	0	2
2981	1	6	1	1	2	0	0	0	2
2982	1	10	1	3	0	0	2	2	2
2983	1	8	1	2	1	1	1	0	2
2984	1	13	2	3	1	0	2	2	3
2985	1	8	2	0	3	0	1	0	2
2987	0	3	1	0	1	0	0	0	1
2988	1	11	1	3	1	0	1	3	2
2989	1	8	1	1	3	0	1	0	2
2990	1	8	2	0	1	0	2	0	3
2991	1	10	2	3	1	0	2	0	2
2993	1	6	1	1	1	0	1	0	2
2995	1	8	2	1	0	0	2	0	3
2996	1	10	2	3	1	0	1	1	2
2997	1	7	2	1	2	0	0	0	2
2998	1	6	1	1	2	0	1	0	1
2999	1	10	2	2	2	0	2	0	2
3000	1	9	1	0	3	0	2	0	3
3001	1	7	2	1	0	0	2	0	2
3002	0	5	1	1	0	0	1	0	2
3003	1	11	2	1	2	0	1	3	2
3004	0	3	0	0	1	0	1	0	1
3005	1	13	2	3	1	1	2	2	2
3006	1	8	1	3	1	1	1	0	1
3007	0	5	1	1	1	0	1	0	1
3008	1	9	1	3	1	0	2	1	1
3010	1	6	1	1	1	1	1	0	1
3011	1	7	3	0	1	0	1	0	2
3012	0	3	0	2	0	0	1	0	0
3013	1	7	1	0	1	0	1	2	2
3015	0	4	1	0	1	0	1	0	1
3016	1	8	1	3	0	1	1	0	2
3017	1	12	2	3	1	0	1	3	2
3019	1	15	3	3	1	0	2	3	3
3020	1	12	2	3	2	2	2	0	1
3021	1	6	1	1	0	0	2	0	2
3022	1	11	2	2	1	0	2	2	2
3023	1	14	2	2	2	1	2	3	2
3026	1	8	1	3	0	1	1	0	2
3028	1	9	2	2	3	0	1	0	1
3029	1	9	1	2	0	0	1	3	2
3030	1	16	3	3	3	3	1	0	3
3031	1	8	1	2	1	2	1	0	1
3032	1	11	2	2	2	0	2	0	3
3033	1	10	1	2	1	0	2	1	3
3034	1	6	1	2	1	0	1	0	1
3035	0	5	1	1	1	0	1	0	1
3036	1	10	1	2	1	3	1	0	2
3037	1	9	2	2	2	0	2	0	1
3038	1	9	2	3	1	0	1	0	2
3039	1	6	1	1	1	0	1	0	2
3041	1	7	2	0	1	0	2	0	2
3042	0	5	1	1	1	0	1	0	1
3043	0	4	1	0	1	0	1	0	1

3045	0	4	1	1	0	0	1	0	1
3046	1	10	2	2	1	1	1	1	2
3047	1	8	2	0	2	1	1	0	2
3048	0	5	1	0	2	0	1	0	1
3049	1	11	2	3	2	0	1	0	3
3050	1	7	1	2	0	1	1	1	1
3051	1	7	1	0	1	0	1	3	1
3052	1	6	1	1	0	0	2	0	2
3053	0	4	0	1	1	0	1	0	1
3054	1	6	1	1	1	0	1	0	2
3056	0	3	0	1	1	0	0	0	1
3058	0	4	1	1	0	0	1	0	1
3059	0	4	1	0	1	0	1	0	1
3060	1	7	1	1	1	0	2	0	2
3061	1	6	1	1	2	1	1	0	0
3062	1	7	2	0	2	0	1	0	2
3063	1	6	2	0	1	0	1	0	2
3065	1	11	3	1	1	0	1	3	2
3066	0	4	1	0	0	0	1	0	2
3067	1	6	1	1	1	0	1	0	2
3069	1	15	3	3	3	1	2	0	3
3070	0	4	1	0	0	0	1	0	2
3071	0	2	0	0	1	0	0	0	1
3073	1	13	2	3	0	1	2	2	3
3074	1	7	2	1	1	0	1	0	2
3076	1	12	3	3	1	0	2	1	2
3078	1	6	1	1	1	0	1	0	2
3081	1	8	1	2	2	1	1	0	1
3082	1	9	2	3	1	0	1	1	1
3083	1	10	2	2	2	0	2	0	2
3084	0	5	1	1	0	1	1	0	1
3085	1	7	2	0	1	0	1	0	3
3086	0	5	1	0	1	0	1	0	2
3087	1	6	2	1	1	0	0	0	2
3088	0	5	1	1	1	0	1	0	1
3089	1	9	2	2	1	0	2	0	2
3090	1	10	3	1	2	1	1	0	2
3091	1	11	2	3	1	0	1	2	2
3092	1	11	3	3	1	0	2	0	2
3093	1	8	2	2	1	0	1	0	2
3094	0	5	1	0	2	0	1	0	1
3095	1	7	1	1	3	0	1	0	1
3097	0	5	1	0	1	0	1	0	2
3099	1	6	1	1	1	0	1	0	2
3100	1	10	1	0	2	0	1	3	3
3104	0	3	1	0	0	0	1	0	1
3105	0	4	1	1	1	0	0	0	1
3106	1	7	1	2	1	1	1	0	1
3107	0	4	0	1	1	0	1	0	1
3108	1	10	1	1	1	0	2	3	2
3109	1	10	2	1	3	0	1	0	3
3110	1	8	2	1	1	0	1	0	3
3111	0	4	1	1	0	0	1	0	1
3113	1	9	2	2	0	0	2	2	1
3114	1	8	1	2	0	0	1	2	2
3116	1	10	1	2	0	1	2	3	1
3117	1	11	2	3	1	0	1	1	3
3120	1	6	1	1	1	1	1	0	1
3121	1	6	1	0	1	0	2	0	2
3122	0	5	1	1	0	0	1	0	2
3123	1	14	2	3	2	2	2	1	2
3124	0	4	1	1	0	0	1	0	1
3125	1	7	1	0	3	0	1	0	2
3126	1	8	2	3	1	0	1	0	1
3127	0	4	1	0	1	0	1	0	1
3128	1	10	2	2	2	0	1	0	3

3129	1	12	3	1	1	0	3	1	3
3130	0	4	1	0	1	0	0	0	2
3131	1	9	2	1	2	0	2	0	2
3132	0	4	1	1	0	0	1	0	1
3133	1	7	1	1	1	0	1	1	2
3134	1	7	2	2	0	0	2	0	1
3135	0	3	0	0	0	0	1	0	2
3136	1	9	2	2	2	0	1	0	2
3137	1	6	2	0	1	0	1	0	2
3138	1	10	1	3	1	1	1	0	3
3139	1	16	2	3	2	2	1	3	3
3140	1	10	2	2	1	0	1	2	2
3142	1	7	2	1	2	0	1	0	1
3143	1	7	2	1	0	0	3	0	1
3144	0	4	1	1	0	0	1	0	1
3145	1	6	1	2	1	0	1	0	1
3147	1	6	1	1	1	0	1	0	2
3148	1	11	2	3	2	1	1	0	2
3149	1	15	3	3	2	1	2	2	2
3150	1	8	1	1	2	0	2	0	2
3151	0	5	1	0	1	0	2	0	1
3153	1	16	2	3	2	1	2	3	3
3154	1	12	2	3	1	0	2	1	3
3155	0	5	1	1	0	0	2	0	1
3156	1	9	1	1	1	0	1	3	2
3157	1	8	1	1	1	0	2	2	1
3158	0	3	1	0	0	0	1	0	1
3160	1	11	2	2	1	1	2	0	3
3161	1	10	2	3	1	1	1	0	2
3162	0	4	0	1	1	0	0	0	2
3163	1	10	1	3	1	0	2	0	3
3164	1	12	3	3	3	0	1	0	2
3165	1	8	2	1	1	0	1	1	2
3166	1	19	2	3	3	3	2	3	3
3167	1	9	2	2	1	0	2	0	2
3168	0	5	1	1	1	0	1	0	1
3169	1	8	2	1	1	1	2	0	1
3170	0	3	1	0	0	0	1	0	1
3171	1	13	2	2	0	0	3	3	3
3172	1	6	2	0	1	0	1	0	2
3173	1	8	1	2	1	0	2	0	2
3174	1	9	1	3	1	0	2	0	2
3175	1	6	1	1	2	2	0	0	0
3176	1	12	2	2	2	0	3	0	3
3177	0	4	1	1	0	0	2	0	0
3178	1	11	2	3	1	0	2	1	2
3179	1	6	2	2	0	0	1	0	1
3180	1	7	2	1	1	1	0	0	2
3182	1	6	2	1	1	0	1	0	1
3183	1	10	2	2	2	0	2	0	2
3184	0	5	2	0	1	0	1	0	1
3186	0	5	1	0	1	0	1	0	2
3187	1	13	2	2	2	0	2	2	3
3188	1	7	1	2	1	0	1	0	2
3189	0	4	1	1	1	0	1	0	0
3190	1	10	2	1	2	0	2	0	3
3193	1	7	2	1	1	0	1	0	2
3194	1	7	2	0	1	0	2	0	2
3197	1	11	2	3	1	1	2	0	2
3198	1	7	1	3	0	0	2	0	1
3199	1	6	1	2	0	0	1	0	2
3200	0	5	0	0	1	0	1	0	3
3201	1	8	2	1	1	1	1	0	2
3202	1	8	1	2	0	0	2	1	2
3206	1	17	3	3	3	3	3	0	2
3207	1	14	3	2	2	0	2	3	2

3208	0	2	1	0	0	0	1	0	0
3209	1	6	2	0	1	0	1	0	2
3210	1	9	1	1	1	0	2	2	2
3212	1	7	1	2	1	0	1	0	2
3213	1	8	2	2	1	0	1	0	2
3214	1	9	2	2	1	0	2	0	2
3217	1	6	1	2	1	0	1	0	1
3218	0	5	1	0	1	0	2	0	1
3219	1	6	1	2	1	0	1	0	1
3224	0	4	1	0	0	0	2	0	1
3225	1	9	1	3	1	0	2	0	2
3226	0	5	1	0	1	0	1	0	2
3230	1	10	2	2	1	0	1	2	2
3231	1	7	2	1	1	0	1	0	2
3232	1	8	2	2	1	0	2	0	1
3233	1	6	1	2	1	0	1	0	1
3235	0	5	1	1	1	0	1	0	1
3236	0	2	0	1	0	0	0	0	1
3238	0	3	0	0	1	0	0	0	2
3239	1	11	2	3	1	0	2	1	2
3240	1	17	3	3	3	0	2	3	3
3241	1	7	1	2	1	0	1	0	2
3242	0	4	0	1	1	0	1	0	1
3243	0	4	2	1	0	0	1	0	0
3244	1	11	2	3	1	1	2	0	2
3245	1	14	3	3	1	0	2	3	2
3246	1	7	1	1	1	0	1	0	3
3248	0	3	1	0	0	0	2	0	0
3250	1	6	1	2	0	0	1	0	2
3251	0	5	1	2	0	0	1	0	1
3252	1	8	1	2	1	1	1	0	2
3254	1	11	1	2	1	0	2	3	2
3255	1	6	1	1	1	0	1	0	2
3256	1	8	2	2	2	0	1	0	1
3257	1	8	2	2	0	0	2	0	2
3258	1	13	3	2	2	1	1	1	3
3259	0	3	0	0	0	0	1	0	2
3260	1	6	1	2	0	0	1	0	2
3261	1	11	2	1	0	0	3	2	3
3262	1	9	2	1	1	0	1	1	3
3264	0	4	1	0	1	0	1	0	1
3265	1	13	2	2	1	0	2	3	3
3266	1	7	1	1	1	0	2	0	2
3267	1	8	2	0	2	0	1	0	3
3268	1	9	2	1	1	0	1	3	1
3269	1	10	1	2	0	0	1	3	3
3270	1	8	1	0	2	2	1	0	2
3271	1	7	1	1	1	0	2	0	2
3272	0	4	1	0	1	0	1	0	1
3273	1	6	1	1	1	0	1	0	2
3274	1	12	3	3	1	0	2	0	3
3275	1	10	2	1	2	0	1	1	3
3276	1	18	2	3	3	2	3	3	2
3277	0	4	1	0	1	0	1	0	1
3278	1	6	1	1	1	0	2	0	1
3279	1	9	2	2	1	2	1	0	1
3280	1	10	1	3	2	0	1	1	2
3283	0	4	1	0	1	0	1	0	1
3284	1	11	2	2	2	0	2	0	3
3285	1	6	1	1	1	0	1	1	1
3286	1	9	1	3	3	0	1	0	1
3287	0	5	1	1	1	0	1	0	1
3288	0	5	1	1	0	0	1	0	2
3290	1	6	1	1	1	0	1	0	2
3291	1	8	2	2	1	0	2	0	1
3292	0	5	0	1	0	1	2	0	1

3293	0	4	1	0	1	0	1	0	1
3294	0	4	1	1	0	0	1	0	1
3295	1	10	1	3	1	0	2	0	3
3297	1	7	2	2	1	0	1	0	1
3300	1	6	1	1	1	0	1	0	2
3301	1	6	1	1	1	0	1	0	2
3302	0	2	0	1	0	0	1	0	0
3305	1	9	2	3	0	1	1	0	2
3306	1	6	2	0	2	0	1	0	1
3309	1	8	1	1	0	0	1	3	2
3312	1	11	1	1	2	2	2	0	3
3313	1	10	2	1	2	1	2	0	2
3314	1	10	2	3	1	0	2	0	2
3315	1	11	3	3	0	0	3	0	2
3317	1	13	3	3	3	0	2	0	2
3318	1	9	2	1	2	0	1	0	3
3319	1	7	1	0	2	1	1	0	2
3320	1	8	1	2	0	0	2	1	2
3321	0	5	1	1	1	0	1	0	1
3322	0	3	1	0	0	0	1	0	1
3323	1	6	1	1	0	0	2	0	2
3324	0	1	0	0	0	0	0	0	1
3325	1	7	1	2	0	0	2	0	2
3326	0	3	1	0	1	0	1	0	0
3329	0	5	2	0	1	0	1	0	1
3330	1	14	3	3	3	0	2	0	3
3331	1	10	2	0	1	0	2	2	3
3333	1	12	2	1	2	0	2	2	3
3334	0	2	1	0	0	0	0	0	1
3335	1	6	1	2	1	0	1	0	1
3336	1	12	3	2	2	1	1	0	3
3338	1	9	1	3	1	1	1	0	2
3339	1	10	1	2	1	1	2	0	3
3340	1	18	3	3	2	1	3	3	3
3341	0	4	0	1	1	0	1	0	1
3342	1	8	1	3	1	0	2	0	1
3343	1	8	2	1	2	1	1	0	1
3344	0	3	1	0	0	0	1	0	1
3346	1	7	1	1	1	0	1	0	3
3347	1	8	2	1	2	0	1	0	2
3348	0	3	1	0	1	0	1	0	0
3349	1	10	2	2	1	1	1	0	3
3350	1	8	2	1	3	0	1	0	1
3354	1	8	2	1	1	0	2	0	2
3355	0	5	1	1	2	0	0	0	1
3356	1	10	2	1	2	1	1	0	3
3357	0	1	0	0	0	0	1	0	0
3358	1	6	1	1	0	0	2	1	1
3361	1	7	1	2	1	0	2	0	1
3362	0	5	1	2	1	0	0	0	1
3363	0	5	1	2	1	0	1	0	0
3364	1	11	2	2	1	1	2	0	3
3365	1	7	1	1	1	1	1	0	2
3366	1	8	2	2	1	0	1	0	2
3369	1	6	1	1	1	0	2	0	1
3370	0	5	1	1	1	0	1	0	1
3371	1	6	1	0	1	0	1	0	3
3374	1	13	2	2	1	1	2	3	2
3375	1	11	2	1	2	0	2	1	3
3376	0	4	1	1	0	0	1	0	1
3377	1	8	1	1	2	0	1	0	3
3378	1	8	1	3	1	0	1	0	2
3379	1	13	3	3	3	0	2	0	2
3380	0	5	1	1	0	0	1	0	2
3381	1	7	2	1	1	0	1	0	2
3382	0	3	0	0	1	0	0	0	2

3383	1	7	2	0	1	0	1	0	3
3384	1	7	2	1	1	0	1	0	2
3385	0	5	1	1	1	0	1	0	1
3386	0	2	0	1	0	0	1	0	0
3387	1	7	1	1	2	0	1	0	2
3388	0	4	0	0	2	0	0	0	2
3389	1	7	2	2	1	0	1	0	1
3390	1	7	2	2	0	0	2	0	1
3391	1	6	1	0	2	2	1	0	0
3392	0	4	1	0	1	0	1	0	1
3393	1	8	2	1	1	0	1	0	3
3395	1	12	2	1	2	1	2	1	3
3396	1	8	2	1	2	0	1	0	2
3397	0	5	1	2	0	0	1	0	1
3398	1	11	2	3	0	1	1	1	3
3399	1	9	3	1	1	1	1	0	2
3400	1	8	1	2	1	0	2	0	2
3402	1	8	2	1	2	1	1	0	1
3403	0	5	1	1	1	0	1	0	1
3404	1	8	2	2	1	1	1	0	1
3405	1	11	2	3	1	0	2	1	2
3406	0	4	1	1	0	0	1	0	1
3407	0	5	1	1	1	0	1	0	1
3409	1	11	2	3	3	0	1	0	2
3411	1	6	1	2	0	0	1	0	2
3412	0	5	1	1	0	0	2	0	1
3413	1	9	2	1	3	0	1	0	2
3414	1	6	1	0	1	0	1	1	2
3415	1	6	1	0	1	0	1	0	3
3417	1	11	3	1	2	0	2	0	3
3418	1	10	2	2	1	1	2	0	2
3419	1	7	1	2	1	0	1	0	2
3420	1	10	1	2	2	0	2	0	3
3422	1	14	2	3	3	2	1	0	3
3423	0	4	1	0	0	0	1	0	2
3424	0	5	1	0	1	0	1	0	2
3426	1	7	2	0	1	0	1	1	2
3427	0	5	2	1	0	0	1	0	1
3428	1	11	3	2	3	0	1	0	2
3429	0	4	1	0	1	0	1	0	1
3430	0	4	0	1	0	0	1	0	2
3431	1	15	2	2	3	3	1	2	2
3433	0	5	1	1	0	0	2	0	1
3434	0	2	0	0	1	0	0	0	1
3436	1	11	1	2	1	0	2	3	2
3437	1	14	2	2	1	1	2	3	3
3439	0	5	1	0	1	0	1	0	2
3441	1	10	1	2	1	1	2	2	1
3442	0	5	0	1	1	0	1	0	2
3444	1	14	2	3	2	2	2	0	3
3446	1	7	1	3	0	0	2	0	1
3447	0	5	1	1	0	0	2	0	1
3449	0	5	1	0	1	0	2	0	1
